# Circulating MicroRNAs in Lung Cancer and Pulmonary Tuberculosis: Diagnostic Potential, Shared Signatures, and Challenges in Differential Diagnosis

**DOI:** 10.3390/diagnostics16213619

**Published:** 2026-11-02

**Authors:** Nazgul Khamitova, Aliya Nurmukhanbetova, Rifat Hamoudi, Madina Kamalieva, Nurlan Jainakbayev, Nurlan Baisynov, Kamalidin Sharipov

**Affiliations:** 1 Aitkhozhin Institute of Molecular Biology and Biochemistry, Almaty 050012, Kazakhstan; nazgul2608@mail.ru; 2 Department of Biophysics, Biomedicine and Neuroscience, Faculty of Biology and Biotechnology, Al-Farabi Kazakh National University, Almaty 050040, Kazakhstan; 3 Department of General Immunology, S.D. Asfendiyarov Kazakh National Medical University, Almaty 050060, Kazakhstan; 4 Research Institute of Medical and Health Sciences, University of Sharjah, Sharjah P.O. Box 27272, United Arab Emirates; rhamoudi@sharjah.ac.ae; 5 Center of Excellence for Precision Medicine, University of Sharjah, Sharjah P.O. Box 27272, United Arab Emirates; 6 Department of Clinical Sciences, College of Medicine, University of Sharjah, Sharjah P.O. Box 27272, United Arab Emirates; 7 Surgical Biotechnology Department, Division of Surgery and Interventional Science, University College London, London WC1E 6BT, UK; 8 Department of Public Health, NEI “ Kazakhstan-Russian Medical University”, Almaty 050000, Kazakhstan; nauka@medkrmu.kz (M.K.); rector@medkrmu.kz (N.J.); 9 Department of Scientific Work, NEI “ Kazakhstan-Russian Medical University”, Almaty 050000, Kazakhstan; baysynov.n@medkrmu.kz

**Keywords:** circulating microRNAs, lung cancer, pulmonary tuberculosis, molecular diagnostics, biomarker panels, differential diagnosis, qRT-PCR, respiratory diseases, non-invasive biomarkers, translational medicine

## Abstract

The differential diagnosis of lung cancer and pulmonary tuberculosis (PTB) remains challenging because the diseases share clinical manifestations and radiological features, particularly in regions with a high tuberculosis burden. This narrative review evaluates circulating microRNAs (miRNAs), primarily measured in serum, plasma and extracellular-vesicle-enriched fractions, as potential adjunctive biomarkers for this differential diagnosis. The literature search was updated through 3 October 2026. Evidence was synthesized by comparator group, biological matrix, analytical methodology and validation design, distinguishing direct lung cancer–PTB comparisons from disease–control studies and prognostic or monitoring investigations. Numerous miRNAs have been associated with either disease, but shared candidates and opposite expression directions across separate studies do not establish differential diagnostic accuracy. Findings vary with patient selection, sample processing, detection platforms, normalization and reference standards. Direct blood-based comparisons remain limited and report variable discrimination. A plasma panel comprising miR-342-3p, miR-199a-3p and miR-199b-3p differentiated PTB from lung cancer with areas under the curve of 0.911 in training and 0.886 in internal split-sample validation; validation sensitivity and specificity were 87.9% and 75.9%, respectively. Its case–control design, incomplete microbiological confirmation and absence of concomitant lung cancer–PTB assessment limit clinical interpretation. Additional pleural-fluid findings concern a separate matrix and cannot be extrapolated to blood testing. Prognostic and treatment-associated findings remain exploratory and do not establish treatment-guiding or validated monitoring utility. Circulating miRNAs should therefore be considered potential adjuncts to clinical assessment, imaging, microbiological testing and histopathology. Clinical translation requires standardized assays, independent prospective validation in clinically relevant populations, and demonstration of incremental value and clinical utility within existing diagnostic pathways. Future studies should prespecify intended use, assay procedures and diagnostic thresholds, include patients with coexisting disease, and evaluate whether testing improves clinical decisions without replacing established microbiological or tissue confirmation methods.

## 1. Introduction

Lung cancer and pulmonary tuberculosis (PTB) represent two major and overlapping global health challenges. According to GLOBOCAN 2022 estimates, lung cancer accounted for approximately 2.48 million new cases and 1.82 million deaths worldwide. It was therefore the most commonly diagnosed malignancy and the leading cause of cancer-related mortality across both sexes [1,2]. Its global burden is expected to rise over the coming decades. Sustained tobacco exposure and ongoing epidemiological changes in low- and middle-income countries are major contributors to this projected increase [2]. PTB, caused by *Mycobacterium tuberculosis*, also remains one of the world’s deadliest infectious diseases. The World Health Organization (WHO) estimated that 10.8 million people developed tuberculosis and approximately 1.25 million died from the disease in 2023 [3]. Importantly, PTB and lung cancer can occur in the same patient. Previous *M. tuberculosis* infection has also been identified as an independent risk factor for the subsequent development of lung cancer [4,5]. Both diseases are particularly prevalent in high-burden regions of South and East Asia, sub-Saharan Africa, and Latin America [4,5]. Their relationship may complicate clinical presentation and delay appropriate treatment, highlighting the need for accurate and timely differential diagnosis [5].

Differentiating lung cancer from PTB at the initial clinical presentation remains a major diagnostic challenge, particularly in resource-limited settings where both diseases are common. They share several nonspecific respiratory and systemic symptoms, including persistent productive cough, hemoptysis, progressive weight loss, low-grade fever, night sweats, and fatigue. No individual symptom can reliably distinguish one condition from the other [6,7]. Their radiological findings also frequently overlap. Chest radiography and CT may reveal pulmonary nodules, parenchymal masses, cavitary lesions, lobar consolidation, pleural effusion, and mediastinal or hilar lymphadenopathy in either disease [6,8]. Imaging findings may therefore be inconclusive during the initial evaluation. Clinical studies have reported cases in which active PTB was initially diagnosed as primary lung cancer. Conversely, early-stage lung cancer may be misclassified as an infectious or granulomatous disease [7,9]. Such errors can delay appropriate treatment and adversely affect patient outcomes. Diagnosis becomes even more difficult when both diseases occur in the same patient. Coexisting PTB and lung cancer further complicate diagnosis and treatment decisions in TB-endemic settings [8,10].

Established diagnostic methods provide essential, complementary information, although overlapping presentations can complicate their interpretation. PTB is primarily diagnosed using sputum smear microscopy, mycobacterial culture, and nucleic acid amplification tests. Xpert MTB/RIF provides high specificity for detecting *M. tuberculosis*, but its sensitivity varies in paucibacillary disease and smear-negative samples [11,12]. Bronchoscopy with bronchoalveolar lavage may improve the diagnostic yield of Xpert MTB/RIF Ultra in patients unable to produce adequate sputum. However, this procedure is invasive, resource-intensive, and not universally available in high-burden regions [13]. Chest radiography and CT are essential for characterizing pulmonary lesions, but they cannot consistently distinguish malignant lesions from infectious processes [6,14]. CT-based radiomics may improve diagnostic differentiation, although prospective studies and external validation are still required [14]. A definitive diagnosis of lung cancer generally requires tissue obtained through bronchoscopy, CT-guided percutaneous biopsy, or surgery. These procedures are invasive, carry procedural risks, and remain vulnerable to sampling error [6,7]. These limitations provide a rationale for evaluating blood-based biomarkers as adjuncts to established diagnostic pathways. Circulating miRNAs are considered here as potential complementary tools; their proposed role does not imply replacement of imaging, microbiological testing, or tissue-based diagnosis.

MicroRNAs (miRNAs) are short, endogenous, single-stranded noncoding RNA molecules that are approximately 18–25 nucleotides long. They regulate gene expression at the post-transcriptional level by binding to complementary sequences in target messenger RNAs. This interaction can suppress translation or promote mRNA degradation [15]. Some miRNAs are released into extracellular biological fluids. The term circulating miRNAs refers to miRNAs detected in peripheral blood, plasma, or serum [15,16]. Extracellular miRNAs can be protected from degradation through association with extracellular vesicles, RNA-binding proteins, or lipoprotein complexes [16,17]. Their stability under different storage conditions and repeated freeze–thaw cycles supports their potential use in clinical testing [17,18]. Circulating miRNAs profiles may reflect contributions from pulmonary tissues, immune cells, and other blood or tissue sources; they cannot be assumed to represent lung-specific processes [18]. Since blood collection is minimally invasive, miRNAs can also be measured repeatedly during diagnosis, treatment, and follow-up [15,16].

Differences and overlaps in the molecular responses associated with malignancy and mycobacterial infection provide a biological rationale for investigating circulating miRNAs in differential diagnosis [19,20,21,22]. In lung cancer, oncogenic transformation and tumor progression disrupt miRNA expression and function. The resulting circulating miRNA profiles may reflect changes in cell proliferation, apoptosis, epithelial-to-mesenchymal transition, angiogenesis, and metastatic dissemination [19,20]. In PTB, the host response to *M. tuberculosis* activates different immunological pathways. These include macrophage activation, granuloma formation, and type I and type II interferon signaling. Changes in peripheral-blood miRNA expression have been associated with these responses, but do not necessarily constitute a PTB-specific signature [19,22]. Some miRNAs involved in inflammation and immune regulation are altered in both tuberculosis and cancer. Other miRNAs appear to be preferentially associated with one condition [20,21]. These shared and condition-associated patterns motivate the evaluation of multi-miRNA panels, although their ability to distinguish lung cancer from PTB must be demonstrated in direct comparative studies. Early comparative studies have identified different serum and plasma miRNA profiles in patients with lung cancer and PTB [20,21].

Studies of circulating miRNAs have reported disease-associated signatures in lung cancer and PTB, but evidence obtained separately for each disease does not establish discrimination between them [23,24]. Direct comparative investigations have also reported preliminary discriminatory performance, including in patients with similar radiological presentations [25]. However, the available evidence remains methodologically heterogeneous. Many studies have included small samples, inconsistent patient selection criteria, and limited information about disease stage, histological subtype, treatment status, and comorbidities [24,26]. Preanalytical and analytical variation includes biological matrix, blood-processing methods, time to processing, RNA extraction platforms, and normalization strategies [23,25,26]. Most studies have evaluated one disease against healthy controls rather than directly comparing lung cancer with PTB. Such studies support biomarker discovery but provide indirect evidence for the differential-diagnosis question. External validation in geographically, demographically, and clinically distinct populations also remains limited [23,25]. These weaknesses create a substantial gap between promising discovery findings and clinical implementation.

A critical synthesis of the available evidence is therefore needed. This review evaluates circulating miRNAs as potential adjunctive blood-based biomarkers for differentiating lung cancer from PTB. It examines their biological basis, disease-associated expression patterns, comparative diagnostic performance, and potential clinical utility. Direct LC–PTB comparisons form the central evidence base for this question, while disease-versus-healthy-control studies and biological findings provide supporting context. Particular attention is given to methodological heterogeneity and translational barriers that limit clinical implementation. The review integrates these findings to assess individual miRNAs and multi-miRNA panels, explain inconsistencies between studies, and identify priorities for prospective evaluation within established diagnostic pathways.

## 2. Materials and Methods

This narrative review was conducted to provide a critical and integrative synthesis of evidence concerning circulating miRNAs in lung cancer, pulmonary tuberculosis (PTB), and their differential diagnosis. A structured literature search was used to support the narrative synthesis; the review was not conducted as a systematic review of diagnostic accuracy. The quality and reporting of the review were assessed using the Scale for the Assessment of Narrative Review Articles (SANRA), as presented in Table S1 [27]. SANRA assessed the quality of the narrative review itself and was not used to assess the methodological quality or risk of bias of the included primary studies.

**Figure 1:**
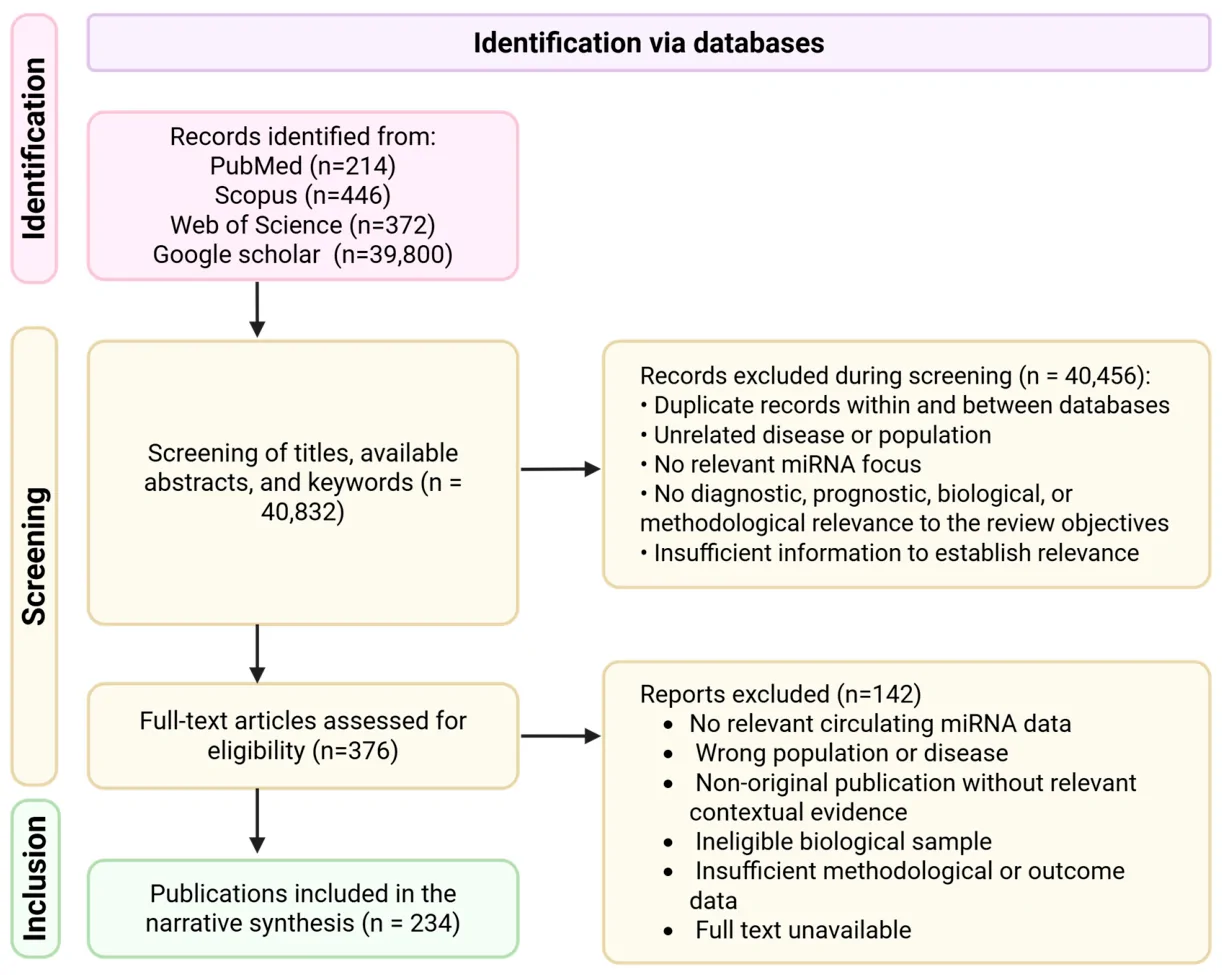
PRISMA flow diagram of the literature search and study selection process (File S1).

The initial literature search and record identification were conducted by Nazgul Khamitova and Aliya Nurmukhanbetova using PubMed, Scopus, Web of Science, and Google Scholar (Figure 1). The initial searches were conducted from 7 August to 21 August 2026. Searches were repeated and expanded, and potentially relevant publications were checked during the update conducted from 27 September to 3 October 2026. The last search was performed on 3 October 2026. No language or publication-date restrictions were applied to the overall search.

Three complementary search categories were applied: circulating miRNAs in lung cancer, circulating miRNAs in PTB, and miRNAs directly investigated for distinguishing lung cancer from PTB. During the update, the direct-comparison strategy was expanded by replacing the required phrase “differential diagnosis” with a broader group of alternative terms—diagnos*, discriminat*, differenti*, distinguish*, and biomarker*—together with additional lung cancer terms. Equivalent word forms were used in Google Scholar. The database-specific search strategies and numbers of records retrieved are provided in Table S2. Initial and updated searches are reported separately. Particular attention was given to publications from 2025 and 2026, without excluding relevant earlier studies.

Records from each database were examined for relevance to the review objectives. Screening tasks were distributed among Nazgul Khamitova, Aliya Nurmukhanbetova, Madina Kamalieva, Nurlan Jainakbayev, and Nurlan Baysynov; Scopus and Web of Science screening was conducted by Nazgul Khamitova and Aliya Nurmukhanbetova. Screening was collaborative rather than independently duplicated by two reviewers. Titles, available abstracts, and keywords where available were assessed in relation to the population, biological matrix, miRNA measurements, comparator groups, and relevance to the review topics. Duplicates within individual database results were identified manually during screening. Potentially relevant records from the four databases were subsequently combined and compared to remove duplicates between databases. Records were also excluded during screening when they concerned unrelated diseases or populations, lacked a relevant miRNA focus, provided no relevant diagnostic, prognostic, biological, or methodological information, or contained insufficient information to establish relevance. Publications already cited in the manuscript were identified separately to avoid counting them as newly added references.

Google Scholar searches were additionally examined using individual publication-year filters. The authors inspected all available results pages within each year-specific search and assessed titles, available abstracts, and keywords where available; potentially relevant records were retained for further assessment. The counts displayed by Google Scholar were approximate search-result estimates and could include overlapping records across queries. These estimates are reported in Table S2 and should be distinguished from unique publications retained after screening and manual deduplication. Detailed record-level logs of excluded Google Scholar results were not retained, although the available results pages were screened. Database-specific screening tables for PubMed, Scopus, and Web of Science are provided in Tables S2.1.1–S2.1.12. Selected Google Scholar records are presented in Table S2.1, and the consolidated publication list, including relevance assessments and identification of references already present in the manuscript, is provided in Table S2.2.

Original human studies were considered eligible if they evaluated circulating or blood-derived miRNAs in patients with lung cancer, PTB, or both conditions and reported diagnostically, prognostically, biologically, or methodologically relevant findings. Studies examining serum, plasma, extracellular vesicles, exosomes, whole blood, or defined blood-cell fractions were considered, provided that the biological compartment was clearly identified. Evidence from other biological matrices, mechanistic studies, reviews, consensus statements, and study protocols was considered separately where relevant to the contextual discussion and was not treated as equivalent to primary circulating-miRNA diagnostic evidence. Study protocols were used to describe planned research rather than completed diagnostic or clinical outcomes.

For the primary-study evidence set, full-text reports were excluded if they contained no relevant circulating-miRNA data, evaluated an ineligible population or disease, were not original research articles, used an ineligible biological sample, or provided insufficient methodological or outcome data. Publications outside this primary-study category were retained only when they contributed relevant contextual evidence. Reports that could not be obtained in full text were not subjected to full-text eligibility assessment. Full-text eligibility was assessed collaboratively by Nazgul Khamitova, Aliya Nurmukhanbetova, Nurlan Baysynov, and Kamalidin Sharipov, rather than independently by two reviewers. Differences in interpretation were discussed with reference to the eligibility criteria and the relevance of the reported findings to the review objectives. Decisions were reached through discussion and consensus. The updated search resulted in the inclusion of 45 additional publications not previously cited in the manuscript, including publications from 2025 and 2026. These supplemented the review with direct comparative, disease-specific, methodological, and mechanistic evidence.

Data were narratively organized according to disease, biological matrix, miRNA or miRNA panel, study population, analytical method, expression direction, diagnostic or prognostic performance, biological relevance, and methodological limitations. Relevant information was extracted from the full-text reports into evidence tables. During collaborative review, extracted findings were checked against the source reports, and uncertainties concerning comparator populations, biological matrices, or reported outcomes were discussed before incorporation into the synthesis. Particular attention was given to consistency across serum, plasma, extracellular-vesicle, and cellular blood fractions; reproducibility across independent cohorts; and evidence directly comparing lung cancer with PTB. Direct LC–PTB comparisons were distinguished from disease-versus-healthy-control studies, comparisons with other pulmonary diseases, and mechanistic or methodological evidence. Owing to substantial heterogeneity in study populations, sample processing, analytical platforms, normalization procedures, and reported outcomes, no quantitative meta-analysis was performed.

## 3. Diagnostic Overlap Between Lung Cancer and PTB

### 3.1. Radiological Overlap and Imaging Limitations

Computed tomography (CT) is central to the radiological evaluation of lung cancer and PTB. However, several morphological features overlap between the two diseases. These include nodule margins, cavity wall characteristics, contrast enhancement patterns, and mediastinal or hilar lymphadenopathy [6]. Spiculation, lobulation, and pleural retraction may suggest malignancy, but none of these findings is specific to lung cancer. Pulmonary tuberculomas may present with the same features. Spiculated margins can result from a desmoplastic reaction surrounding lung adenocarcinoma or from inflammatory fibrosis surrounding a tuberculoma [28,29,30]. Several forms of lung cancer may therefore resemble specific manifestations of PTB, including tuberculoma, cavitary disease, consolidative PTB, endobronchial tuberculosis, and miliary tuberculosis (Table S3). These overlapping presentations share clinical and radiological features, while findings favoring either diagnosis remain suggestive rather than confirmatory (Table 1). Diagnostic evaluation therefore requires presentation-specific microbiological testing and cytological or histopathological assessment where appropriate (Table 2).

Fluorine-18 fluorodeoxyglucose positron emission tomography combined with computed tomography (18F-FDG-PET/CT) provides metabolic information but has limited specificity in regions where PTB is prevalent. Tuberculomas, cavitary lesions, and affected lymph nodes may demonstrate maximum standardized uptake values (SUVmax) that overlap with those of malignant lesions [31,32,33]. Conversely, lepidic-predominant adenocarcinomas and ground-glass nodules may show low metabolic activity and produce false-negative findings in early lung cancer [34]. CT-based radiomics can identify complex imaging features beyond conventional visual interpretation. Reported areas under the receiver operating characteristic curve (AUCs) have ranged from 0.80 to 0.92 [29,35,36]. However, these estimates should be interpreted in relation to study design and validation, because single-center development does not establish performance in independent populations. Radiomics therefore remains an adjunctive approach requiring further validation. CT and 18F-FDG-PET/CT characterize morphology and metabolic activity but do not establish a definitive etiological diagnosis.

### 3.2. Major Diagnostic Difficulty Presentations

Three clinical and radiological presentations illustrate important diagnostic difficulties (Table 1). The first is upper lobe cavitary disease in smokers older than 50 years. This population is at increased risk of both cavitating squamous cell carcinoma and cavitary post-primary PTB. Cavity wall thickness, inner wall contour, and satellite nodules may support diagnostic assessment, but substantial overlap exists between the two diseases. These features should therefore be interpreted together rather than used as independent diagnostic criteria. Tissue and microbiological sampling are therefore often required [37,38,39].

The second presentation involves diffuse bilateral miliary micronodules. Both miliary PTB and hematogenous metastatic lung cancer may cause acute respiratory deterioration and systemic illness. Accurate differentiation is essential because the two conditions require fundamentally different treatments [40,41]. The small size and diffuse distribution of the nodules can complicate selection of a suitable sampling site (Table 2).

The third presentation is mediastinal or hilar lymphadenopathy with central necrosis. Peripheral rim enhancement is commonly associated with PTB, but it may also occur in necrotic lung cancer metastases and other granulomatous diseases. Its diagnostic value therefore depends on the prevalence of these diseases in the population being examined [7,32,42]. Vascularized tissue surrounding caseous necrosis may produce an enhancement pattern similar to that surrounding necrotic primary tumors or metastatic lymph nodes [6,42]. Endobronchial ultrasound-guided transbronchial needle aspiration (EBUS-TBNA) allows material from accessible lymph nodes to be obtained for both cytological and microbiological assessment [43].

### 3.3. Biological Basis of the Diagnostic Overlap

The overlap between lung cancer and PTB may partly reflect biological responses that can produce similar metabolic and molecular patterns. In active tuberculosis, granuloma formation involves the recruitment of macrophages, dendritic cells, and cluster of differentiation 4-positive (CD4+) T lymphocytes. Activation of these immune cells may involve increased glycolytic activity as part of the response to *M. tuberculosis*. Their increased glucose metabolism may contribute to 18F-FDG uptake that may resemble the metabolic activity of malignant tissue [32,33].

Granulomatous and malignant lesions may also activate pathways involving hypoxia-inducible factor 1-alpha (HIF-1α), mechanistic target of rapamycin (mTOR), and vascular endothelial growth factor (VEGF) [5,6]. These pathways operate in different cellular and pathological contexts. However, their activation may contribute to overlapping inflammatory, vascular, fibrotic, and metabolic changes. Such overlap does not imply identical mechanisms but helps explain why morphology and metabolic activity alone may not reliably distinguish PTB from lung cancer.

### 3.4. Limitations of Sputum-Based PTB Testing

A negative sputum test alone does not exclude PTB and should not be used to establish an alternative diagnosis of lung cancer without further investigation. Conventional acid-fast bacillus (AFB) sputum smear microscopy has variable sensitivity, depending on bacillary load, specimen quality, collection technique, and laboratory proficiency. Consequently, some patients with culture-confirmed PTB may have negative sputum smears during the initial assessment [5,6].

Negative or low-grade smear results are particularly common in paucibacillary disease. This pattern may occur in endobronchial PTB, consolidative or pseudotumoral PTB, and immunocompromised patients. Relevant conditions include human immunodeficiency virus (HIV) infection, diabetes, malnutrition, and chronic corticosteroid use. In these settings, the release of *M. tuberculosis* into expectorated sputum may be limited or intermittent [7]. Xpert MTB/RIF Ultra generally provides greater sensitivity than conventional smear microscopy, although its performance varies according to specimen type, bacillary burden, and patient population [11,12,13]. Previous PTB treatment may reduce diagnostic specificity. Access may also be restricted by reagent costs and laboratory infrastructure in high-burden regions.

Mycobacterial culture on Löwenstein–Jensen medium remains an important reference method for confirming PTB. However, results may require 6–8 weeks, and cultures remain susceptible to contamination. When sputum testing is inconclusive, ronchoscopic sampling may improve diagnostic yield, particularly in endobronchial and consolidative disease, where organisms may be absent from expectorated sputum [7,43,44]. The choice of additional respiratory or tissue sampling should follow the lesion’s location and clinical presentation (Table 2).

### 3.5. Integrated Histopathological and Microbiological Confirmation

Differentiating lung cancer from PTB may require complementary histopathological and microbiological assessment. Histopathology alone may not confirm PTB because granulomatous inflammation is not specific to *M. tuberculosis*. Similar tissue responses may occur in fungal infections caused by *Histoplasma* or *Cryptococcus*, as well as in sarcoidosis, beryllium exposure, and foreign-body reactions. Ziehl–Neelsen staining may also fail to identify acid-fast bacilli in granulomatous specimens [6]. Microbiological confirmation should therefore be obtained whenever possible through mycobacterial culture or a validated molecular assay such as Xpert MTB/RIF [11,12,13].

Cytological findings must also be interpreted carefully. Reactive atypia in inflammatory samples should be distinguished from unequivocal malignant findings, and microbiological assessment remains relevant when PTB is suspected. An additional challenge is that lung cancer and PTB may occur simultaneously in the same patient or anatomical region. Coexisting disease has been reported in resected pulmonary specimens and selected autopsy series from TB-endemic settings [7,8,14,45]. A published case report further illustrates how conflicting clinical, imaging, pathological, and microbiological findings can complicate differentiation between PTB and lung cancer [46].

For suspected lung cancer, pathological assessment also establishes tumor type; the 2015 WHO classification emphasizes the integration of morphology and immunohistochemistry in small biopsies and cytological samples [47]. When mediastinal or hilar lymph nodes are accessible, EBUS-TBNA allows cytological assessment, mycobacterial culture, and molecular testing to be performed using material obtained during the same procedure [43]. Inconclusive or discordant results warrant multidisciplinary review involving pulmonology, oncology, radiology, pathology, and clinical microbiology.

Additional approaches have examined immunocyte counts combined with CT features to distinguish non-calcified tuberculomas from malignant solitary pulmonary nodules [48], and dual-energy spectral CT to distinguish solitary PTB from lung adenocarcinoma [49]. These address specific comparator populations and should not be extrapolated to all overlapping presentations. A systematic review comparing PTB with concomitant lung cancer against PTB alone addresses a separate diagnostic question: recognizing coexisting malignancy rather than distinguishing mutually exclusive diseases [50].

### 3.6. Rationale for Circulating miRNA-Based Diagnostics

The limitations of current diagnostic methods provide a rationale for evaluating circulating miRNAs as potential adjunctive blood-based biomarkers. Their proposed role is to add molecular information to established diagnostic pathways, rather than replace CT, microbiological testing, or tissue examination. Tissue sampling carries procedural risks and may be difficult in patients with diffuse disease or anatomically inaccessible lesions [5,6].

Malignant transformation and the immune response to *M. tuberculosis* may be associated with partly overlapping patterns of miRNA regulation. Lung cancer-associated miRNAs have been linked to tumor-related processes, while PTB-associated miRNAs have been associated with immune and inflammatory responses [19,20,21,22,23,24,25]. However, circulating profiles may arise from several cellular and tissue sources, and associations with either disease do not establish LC–PTB discrimination. Shared and condition-associated changes provide a rationale for evaluating multi-miRNA panels, but their discriminatory value requires direct comparative testing.

Circulating miRNAs can be measured in plasma or serum using reverse-transcription quantitative polymerase chain reaction (RT-qPCR). A prospectively validated miRNA panel could complement CT, microbiological testing, and tissue examination, including in TB-endemic regions. Whether its addition reduces unnecessary invasive procedures or shortens time to diagnosis remains a clinical question requiring prospective evaluation.

## 4. Circulating miRNAs Associated with Lung Cancer

### 4.1. General Evidence and Diagnostic Concept of Circulating miRNAs

Circulating miRNAs are being explored as biomarkers across the clinical continuum of lung cancer. An editorial commentary on early screening studies described serum and plasma miRNA signatures with potential relevance to lung cancer detection [51]. A protocol addressing serum miR-21 outlined planned evidence synthesis but provided no diagnostic results [52]. Their potential adjunctive use alongside low-dose computed tomography has been discussed for risk stratification and assessment of suspicious pulmonary nodules [53]. Other reviews have examined associations with histological subtype, tumour behaviour and treatment-related changes [54], as well as individual plasma miRNAs and panels for NSCLC detection [55]. Systematic reviews of Western [56] and East Asian populations [57] summarized diagnostic findings while identifying variation in panel composition and validation. Their proposed role as supplements to low-dose computed tomography remains under evaluation [58]. Changes observed after surgery or during treatment may be associated with tumour burden and disease activity, although circulating profiles may also be influenced by inflammation and blood-cell contributions [59]. Collectively, these findings support further evaluation of circulating miRNAs as adjunctive biomarkers for lung cancer detection, classification and longitudinal assessment (Table 3). This literature provides contextual evidence rather than establishes discrimination between lung cancer and PTB.

The reviewed studies reported higher median sensitivity and specificity for multi-miRNA signatures than for individual miRNAs, although these descriptive comparisons do not establish superiority in matched head-to-head evaluations [56]. Some panels achieved high diagnostic performance, but their composition and validation strategies varied considerably across studies [56,57]. Earlier screening studies also reported promising results for serum and plasma miRNA classifiers; however, these estimates were summarized in secondary literature rather than independently established by the reviews themselves [51]. Panels may capture complementary molecular changes and reduce reliance on individual markers, while miRNA ratios may provide internal normalization and reflect coordinated expression patterns [56]. Nevertheless, no common diagnostic signature has emerged. Panels differ in the number and expression direction of their constituent miRNAs and may incorporate ratios or weighted risk scores, complicating direct comparisons across studies [56,57]. Consequently, high accuracy within a single cohort does not establish reproducibility across populations. Discovery and internal-validation findings must be distinguished from independent external validation, while prospective evaluation addresses performance under predefined clinical testing conditions. Overall, multi-miRNA signatures warrant further investigation, but their clinical utility depends on standardized panel composition, reproducible analytical procedures, and independent validation in clinically relevant populations.

Circulating miRNA findings depend on the biological matrix and biomarker model. Serum and plasma are not interchangeable, as coagulation, platelet activation and pre-analytical processing can alter miRNA profiles [55,59]. Whole-blood measurements may reflect blood-cell composition, while extracellular-vesicle-associated miRNAs should be distinguished from total circulating miRNAs [59]. Interpretation also depends on whether the study evaluates an individual miRNA, a multi-miRNA panel, a combined model or an analytical platform [55,58]. Diagnostic estimates are influenced by cancer type, stage, comparator population and validation design. Comparisons with healthy controls differ from those involving benign pulmonary disease or indeterminate nodules, and neither comparison directly establishes LC–PTB discrimination. Internal validation assesses performance within the source population, whereas independent external validation evaluates transferability to a separate population. Prospective evaluation addresses a different design feature and does not, by itself, constitute external validation [56,57].

### 4.2. Serum-Based Circulating miRNAs

Serum-based miRNA research has progressed from exploratory profiling to the development of multivariable diagnostic signatures (Table 4 and Table S4). In an early proof-of-principle study, Keller et al. analyzed 29 samples, comprising 23 serial samples from eight patients and six samples from healthy controls [60]. Serum miRNA profiles changed as diagnosis approached and largely separated prediagnostic, postdiagnostic, and control samples. However, no predefined classifier, ROC analysis, RT-qPCR confirmation, or independent validation was provided. Serum miR-23a distinguished lung adenocarcinoma from COPD with an AUC of 0.896, 75.0% sensitivity, and 92.7% specificity [61]. However, the analysis included small cohorts and focused on one histological subtype. The 34-miRNA classifier reported by Bianchi et al. achieved an AUC of 0.890 in the testing cohort, but its sensitivity for stage I disease was only 59% [62]. A miR-15b/miR-27b classifier reached 100% sensitivity and 84% specificity in an independent test set, although healthy individuals were used as controls [63]. A ten-miRNA panel developed by Chen et al. achieved AUCs of 0.966 and 0.972 in the training and validation sets, respectively [64]. The separate validation cohort should not be classified as external solely because it was distinct from training, and the healthy-control comparison did not establish discrimination from benign pulmonary disease. Rani et al. evaluated eight individual candidates, retaining legacy designations such as miR-30c-1 and miR-616, but provided no independent diagnostic validation [65]. Huang et al. reported discriminatory performance for let-7i-3p and miR-154-5p in male participants, although smoking-associated changes complicated attribution to lung cancer [66]. These early studies illustrate differences in comparator selection, marker definition and validation rather than a consistent improvement in diagnostic performance.

Subsequent studies included clinically relevant disease controls and models combining different biomarker classes. Wang et al. evaluated a five-miRNA panel in multicenter and multiethnic cohorts. Their five-miRNA panel distinguished NSCLC from benign pulmonary nodules with an AUC of 0.979 and achieved 95% sensitivity and 84% specificity in a blinded American cohort [67]. This independent validation strengthens evidence for transferability, although prospective screening utility remained unestablished. Adding CYFRA21-1 to miR-652 and miR-660 increased the testing AUC from 0.858 to 0.943, with 85.1% sensitivity and 93.4% specificity [68]. These estimates apply to the protein–miRNA combination. Halvorsen et al. reported independent external validation of a six-miRNA panel in high-risk participants, with AUCs of 0.890 overall and 0.880 for stages I–II [69]. Peng et al. evaluated a different composite model containing three miRNAs and lncRNA MALAT1; its validation AUC of 0.844 should therefore not be attributed to a miRNA-only panel [70]. Other reported markers were less directly diagnostic. Reduced miR-147 was associated with advanced stage, lymph-node metastasis, tumor size, and poorer survival, but no ROC analysis or diagnostic cutoff was reported [71]. This study contributes association and prognostic evidence rather than diagnostic-accuracy evidence. A small study comparing serum and exhaled breath condensate reported serum miR-21 discrimination against healthy controls, without independent validation [72]. Studies of miR-22, miR-125b and miR-15b included benign lung disease controls but incompletely specified comparator-specific performance and validation [73]. A four-miRNA signature was also evaluated against benign pulmonary disease and healthy controls, although its small discovery cohort and predominantly advanced cases restricted clinical interpretation [74]. These findings support separating disease–control comparisons from healthy-control comparisons and distinguishing diagnostic models from prognostic associations.

Serum extracellular-vesicle (EV)-derived miRNAs represent a distinct biomarker category and should not be combined with total-serum measurements. The study of miR-126 identified differences between total serum, EV-associated and EV-depleted fractions, together with stage-associated and functional findings; these results did not establish independently validated diagnostic performance [75]. EV enrichment also does not establish an exclusively tumor-derived signal. Among total-serum models, Fan et al. evaluated five miRNA ratios against benign nodules and inflammatory pulmonary disease, reporting different performance for the two comparator groups [76]. The reported direction of a ratio should not be interpreted as the expression direction of every component. Wang et al. evaluated a miR-21/let-7a model against cancer-free controls, but the absence of independent validation and incomplete characterization of control pulmonary status limited interpretation [77]. A four-miRNA panel evaluated against healthy controls and COPD achieved a test AUC of 0.93, although the separate test set did not by itself establish external validation [78]. Using an independent blinded validation set, miR-223 achieved an AUC of 0.808, with 74.3% sensitivity and 78.2% specificity for stage I–II NSCLC [79]. The blinded assessment reduces some evaluation bias, but external independence of the recruitment setting was not established and benign pulmonary nodules were not included. A miR-182/miR-200b/miR-205 panel was examined in a small healthy-control study without independent validation [80]. Another model combined miR-20a and miR-210 with CEA and NSE for early SCLC assessment; its findings concern the complete four-marker model rather than the miRNAs alone [81]. Thus, comparator population, biological fraction and model composition must accompany any reported performance estimate.

Further studies evaluated EV-associated markers, specialized platforms and larger total-serum classifiers. Serum EV-associated miR-1290 achieved an AUC of 0.937, with 80.0% sensitivity and 96.7% specificity, and decreased after tumor resection [82]. The postoperative change represents a monitoring association and does not independently establish clinical monitoring utility. A specialized EV-capture biochip achieved an AUC of 1.000 for early-stage NSCLC [83]. However, this result was obtained in a small proof-of-concept cohort and cannot be considered confirmed clinical performance. Prospective recruitment in a sequencing-based study of advanced NSCLC supported expression and stage-association analyses, but did not establish external validation of a diagnostic classifier [84]. A panel of serum EV-associated miR-20b-5p and miR-3187-5p produced an AUC of 0.838 for stage 0–I NSCLC. Its AUC increased to 0.930 when combined with CEA [85]. The miRNA-only and protein–miRNA estimates must remain separate. Computational reanalysis of an existing serum dataset used internal cross-validation and synthetic oversampling, providing model-development evidence rather than new clinical validation [86]. Ying et al. evaluated a five-miRNA panel in 744 patients and 944 controls across Asian and Caucasian populations, obtaining validation AUCs of 0.916–0.973 [87]. The large, multiethnic sample does not remove healthy-control spectrum bias or establish prospective utility. Serum miR-21 also showed diagnostic potential, with an AUC of 0.84 and 97% specificity [88]. Nevertheless, it was evaluated in only 30 cases and 30 controls, and most patients had advanced disease. A miR-942/miR-601 model included benign pulmonary disease controls but lacked independent external validation [89]. Asakura et al. subsequently applied comprehensive profiling to more than 3500 participants. Their two-miRNA model achieved a validation AUC of 0.996, although the retrospective biobank design and different source populations introduced potential selection and sample-source bias [90].

Later studies further illustrate why apparently similar accuracy estimates represent different clinical and analytical questions. A pilot model combined serum miR-21 and miR-223 with a urinary metabolite, but incomplete sample overlap and very small modeling subsets created substantial overfitting concerns [91]. Its composite performance cannot be attributed solely to serum miRNAs. By contrast, EV-associated miR-23a and let-7i showed AUCs of 0.744 and 0.733, and their combination did not improve discrimination [92]. A serum miR-145/miR-21/miR-197 panel reported an AUC of 0.939 against healthy controls without independent validation [93]. Smoking-stratified analyses in women with adenocarcinoma identified different performance for miR-532-5p, miR-25-3p and miR-133a-3p, emphasizing population and exposure dependence [94]. Prospective case–control recruitment was also used to evaluate individual markers in early lung cancer, but it did not constitute independent validation of a predefined classifier [95]. Serum miR-4429 distinguished NSCLC from healthy controls with an AUC of 0.918, while its EGFR-related and survival associations addressed separate questions [96]. A longitudinal study involving lung, colon and breast cancers reported overall cancer-classification performance; those estimates should not be presented as lung-cancer-specific diagnostic accuracy [97]. A sequencing-derived four-miRNA panel underwent separate training and validation in a regional NSCLC population, but remained untested in an independent external population [98]. Collectively, these studies support comparator-specific interpretation rather than a general claim that panels outperform individual markers.

Methodological heterogeneity further limits comparisons among serum-miRNA studies. Sequence-level annotation is one source of this heterogeneity. Aiso and Ueda showed that the apparent increase in total miR-1246 was driven mainly by a shorter 5′-isomiR, whereas canonical miR-1246 was decreased [99]. Thus, reporting only “miR-1246 upregulation” would conceal biologically opposite expression patterns and could impair assay reproducibility. Sequencing and automated machine learning subsequently produced a validation AUC of 0.98, although a separate validation cohort did not establish external independence [100]. A 15-miRNA model evaluated against nonmalignant pulmonary disease achieved an AUC of 0.96 ± 0.04 through internal cross-validation, leaving external performance unresolved [101]. By contrast, a prospective population-based study evaluated a prediagnostic miRNA score originally developed for colorectal cancer; its association with subsequent lung cancer risk was not a contemporaneous diagnostic-accuracy estimate [102]. The report attributed to Yang et al. contains cohort sizes and performance estimates matching those in the earlier multiethnic panel report, suggesting possible overlapping evidence that requires confirmation before the publications are counted as independent corroboration [103]. Similarly, miR-155-5p was evaluated mainly as an adjunct to low-dose computed tomography rather than as an independent marker [104]. Any improvement therefore concerns the combined testing strategy. Compartment-specific let-7a-5p and miR-21-3p findings in total serum and EVs concerned stage and histological associations, without a diagnostic ROC model [105]. A multiplex biosensor demonstrated analytical detection in synthetic and spiked serum, but its detection limit does not establish clinical sensitivity, specificity or patient discrimination [106]. Analytical platforms range from microarrays and RT-qPCR to ddPCR, next-generation sequencing, and biosensors. Together with differences in sample processing and normalization, these platform and endpoint differences limit direct comparisons of reported results (Table S4).

Recent publications extend the evidence without resolving these limitations. A serum miR-21/miR-210/miR-942 model for early adenocarcinoma achieved a test AUC of 0.890 using an internal training/test split, but its healthy-control comparison did not represent the full benign-nodule spectrum [107]. Serum miR-107 produced an AUC of 0.952, with 100% sensitivity and 87.9% specificity, in a small retrospective NSCLC–healthy-control cohort without independent validation [108]. A prospectively recruited case–control study reported an AUC of 0.825 for miR-221-3p, but predominantly advanced disease and healthy controls restricted its relevance to early detection [109]. Overall, serum miRNAs remain potential adjunctive biomarkers whose clinical utility has not been established. Most studies used retrospective case–control designs and compared cancer cases with healthy individuals. Such comparisons may produce high AUCs but do not establish discrimination from benign nodules, COPD, tuberculosis, or inflammatory lung disease. Even comparisons with nonmalignant pulmonary disease do not establish LC–PTB discrimination unless PTB is specifically included and analyzed. Internal cross-validation or random data splitting cannot substitute for testing in an independent population. Prospective evaluation and external validation address different design features and should be reported separately. Major interpretive concerns include patient-selection and spectrum bias, the adequacy of disease reference standards, model overfitting and the independence of validation; the limitations summarized here do not constitute a formal risk-of-bias assessment. Translation will require standardized pre-analytical procedures, sequence-specific assays, predefined classifiers and cutoffs, and direct comparison with current diagnostic pathways.

### 4.3. Plasma-Based Circulating miRNAs

Plasma miRNAs have been evaluated across a broader clinical pathway than simple cancer–control discrimination (Table 5 and Table S5). Early studies examined their use for primary diagnosis and treatment assessment. Wei et al. reported that plasma miR-21 distinguished NSCLC from healthy controls with an AUC of 0.775, 76.2% sensitivity, and 70.0% specificity [110]. Higher levels were also observed in advanced disease and in patients with stable or progressive disease after chemotherapy. These associations do not establish treatment-predictive utility. Aushev et al. subsequently reported postoperative reductions in five plasma miRNAs in patients with SCC, without establishing diagnostic accuracy or an exclusively tumour-derived origin [111]. Fortunato et al. examined miRNA-ratio classifiers and pre-analytical interference; the reported sensitivity of 88% and specificity of 93% concerned haemolysis detection, not lung cancer diagnosis [112]. Sestini et al. investigated prognostic risk classification and longitudinal changes within LDCT screening programmes, which represent endpoints distinct from initial diagnostic discrimination [113]. Yan et al. evaluated plasma miR-31 for diagnosis and prognosis, although an inconsistency between the reported AUC and confidence interval limits interpretation of the diagnostic estimate [114]. Powrózek et al. examined miR-944 and miR-3662 for early-stage and histological discrimination, but without independent diagnostic validation [115]. These studies illustrate the need to distinguish diagnostic accuracy from expression associations, prognosis and monitoring.

Plasma signatures have also been investigated for histological classification, longitudinal monitoring, and integration with LDCT. Lu et al. evaluated 1132 participants from five centers [129]. Their six-miRNA panel distinguished lung cancer from high-risk controls with a validation AUC of 0.868. A second panel differentiated SCLC from NSCLC with an AUC of 0.869. Performance was lower for stage I–II cancer, with an AUC of 0.824, 74% sensitivity, and 73% specificity. These results concern cancer detection and histological classification rather than direct LC–PTB discrimination. Postoperative studies showed that plasma miRNA profiles may decrease after resection or change with recurrence [111,113,145]. Nevertheless, monitoring findings cannot be interpreted as evidence of initial diagnostic accuracy or validated detection of residual disease or recurrence. The prospective, single-arm BioMILD study evaluated LDCT together with a 24-miRNA classifier in 4119 high-risk participants and identified 119 lung cancers, including 72 stage I cases [147]. The combined strategy was evaluated for lung cancer risk stratification and risk-adapted screening intervals. However, the study was not a randomized mortality trial. Its findings therefore concern the combined screening strategy and should not be attributed to miRNAs alone. Thus, diagnostic discrimination, histological classification, treatment monitoring, and screening stratification represent distinct clinical endpoints and provide different levels of evidence.

Interpretation of plasma miRNAs also requires attention to the biological and pre-analytical composition of plasma. Measured profiles may include tumor-derived miRNAs together with miRNAs released from platelets, erythrocytes, and leukocytes or other tissues affected by inflammation and injury. Anticoagulant selection, centrifugation, residual platelets, and hemolysis can therefore alter the apparent signal. The haemolysis-related classifier described by Fortunato et al. illustrates the importance of separating sample-quality assessment from cancer detection [112]. This is particularly relevant to studies using miR-16 as a reference or including miR-16-5p and miR-451a as diagnostic markers [119,122,142]. Plasma expression also does not necessarily reproduce tumor-tissue expression. For example, miR-30a was elevated in plasma but reduced in tumor tissue [117]. This discrepancy may reflect selective secretion, release from blood cells, or differences in extracellular clearance rather than analytical error. Ratio-based models were introduced partly to reduce dependence on a single normalization control [112,132]. However, ratios remain sensitive to changes in either component. A ratio classifier or multivariable risk score should therefore not be assigned a single upregulated or downregulated expression direction. Gargiuli et al. further demonstrated acceptable reproducibility within individual platforms but variable concordance across platforms [148]. Plasma-miRNA measurements should therefore be interpreted as composite circulating signals, not as direct surrogates of tumor expression. Without rigorous control of hemolysis and plasma processing, their tumor specificity remains uncertain.

The credibility of multivariable plasma signatures depends strongly on how they were validated. A miR-448/miR-4478 model achieved an AUC of 0.896 for operable NSCLC, but it was evaluated without an independent cohort [116]. Similarly, the miR-19b/miR-183 model produced an AUC of 0.990, with 94.7% sensitivity and 95.2% specificity [122]. However, it included no stage I cancers and was fitted to a case–control population using healthy controls. The phased study by Wang et al. included blinded testing, but separate test sets should not automatically be described as independent external validation without confirming recruitment and sample independence [120]. Leng et al. reported 91.2% sensitivity and 96.7% specificity in a separate validation set; its external independence requires distinction from separation within the same study framework [124]. The multicentre study by Lu et al. likewise included distinct development and validation phases, whose interpretation depends on participant and centre independence [129]. Jiang et al. reported a stage I AUC of 0.942 and included benign lung diseases, although training and testing remained within one study center [145]. Ratio-based models also showed high fitted performance, but bootstrap validation or internal data splitting does not substitute for external testing [132]. Differences in accuracy across studies cannot be attributed to validation design alone because populations, comparators, assays and models also differ. Discovery findings, fitted performance, bootstrap or cross-validation, internal splits, same-centre temporal validation and independent external validation should be reported separately. Prospective evaluation concerns the timing and clinical application of testing and does not, by itself, constitute external validation. Numerical inconsistencies in [114,126], possible cohort overlap among [115,116,118], and the conference-abstract status of [118] are identified in Table 5 and Table S5; these reports should not be treated as interchangeable or necessarily independent evidence.

Pulmonary-nodule classification provides a more clinically relevant test of malignancy discrimination than discrimination between cancer and healthy controls. Lin et al. combined miR-205-5p and miR-210 with nodule diameter [125]. The model achieved an AUC of 0.95 in development and 0.94 in two validation cohorts. However, the contribution of the miRNAs cannot be separated from that of the radiological variable. Cheng et al. demonstrated the effect of comparator selection particularly clearly [131]. The AUC of miR-544a was 0.841 against healthy controls but only 0.638 against benign nodules. A model combining three miRNAs with CT features achieved an AUC of 0.929 in training, but this fell to 0.781 in a temporally separated validation set [138]. This represents same-centre temporal validation rather than independent external validation. The small-EV CirsEV-miR model was evaluated in training, internal-test and external cohorts, with the corresponding results reported separately in Table 5 [149]. Neither healthy-control nor broadly defined benign-nodule comparisons establish LC–PTB discrimination.

Studies explicitly including PTB comparators are therefore particularly relevant to the review’s purpose. Pu et al. included only five PTB patients in a pilot expression study and reported no ROC analysis [121]. Ashirbekov et al. examined direct LC–PTB expression differences, but without independent external validation [159]. Liu et al. developed a three-miRNA classifier for direct PTB–LC discrimination [160]. Of 308 recruited participants, 272 had eligible samples: 110 PTB, 97 LC and 65 healthy controls. The classifier used 77 PTB and 68 LC patients for training and 33 PTB and 29 LC patients for internal validation. Training AUC was 0.911; validation AUC was 0.886, with 87.9% sensitivity and 75.9% specificity for identifying PTB. LC confirmation was primarily histological or cytological, whereas 42 recruited PTB patients lacked microbiological confirmation and were diagnosed using clinical and radiological findings with treatment response. The study did not evaluate concomitant LC–PTB. These limitations restrict generalisation to diagnostically uncertain clinical populations.

Plasma EV-derived miRNAs require separate interpretation from unenriched plasma measurements, although EV-associated miRNAs are themselves extracellular, cell-free components. In operable SCC, five miRNAs were enriched in the exosomal fraction and decreased after tumor removal, supporting a possible tumor association [111]. Neither finding confirms an exclusively tumour-derived source. Exosomal miR-342-5p and miR-574-5p achieved an AUC of 0.813, with 80.0% sensitivity and 73.2% specificity [139]. More complex EV models did not show consistently better performance. A model combining EV let-7b-5p and miR-184 with circulating miR-22-3p achieved an AUC of 0.924, but validation was limited to leave-one-out analysis [150]. An EV ratio algorithm reported 100% sensitivity and specificity, yet it included only 17 cases and 17 controls [152]. The small cohort and limited validation create a substantial risk of overfitting; the reported performance does not establish clinical utility. Moreover, EV-associated markers were not retained in the final SCLC classifier reported by Zaporozhchenko et al. [153]. EV enrichment therefore should not be assumed to improve tumor specificity. ExoQuick precipitation, ultracentrifugation, and small-EV isolation recover different particle populations. Models combining EV-enriched and unenriched plasma measurements also require separate analytical workflows, increasing implementation complexity. Consequently, fraction-specific signatures require clearly defined isolation procedures, consistent RT-qPCR reporting where applicable, and validation in independent clinical cohorts.

Longitudinal changes in plasma miRNAs may provide information beyond initial diagnosis, but current evidence remains inconsistent. Treatment-response associations and postoperative changes are summarised separately from diagnostic accuracy in Table 5 and Table S5 [110,111,113,139,145]. However, miR-544a unexpectedly increased after surgery or chemotherapy [131]. This finding illustrates that treatment-related changes are not uniformly interpretable. Surgery can alter inflammation, platelet activation, tissue injury, and miRNA clearance. A single postoperative measurement therefore cannot establish reliable disease monitoring. Clinical use will require predefined sampling intervals, repeated measurements, and prospective correlation with residual disease and recurrence. Analytical transferability is another barrier. Cross-platform variability and limited cross-cohort reproducibility remain concerns [148,155]. A rapid sequencing workflow was completed within three days, but its overall NSCLC panel achieved only 66.7% sensitivity and 68.2% specificity [154]. Thus, analytical speed does not guarantee clinical accuracy. Public-data analyses must also be distinguished from clinical plasma validation when the biological matrix or cohort independence is uncertain [158]. In the prospective multicentre study by Samejima et al., validation AUC was 0.779 for the miRNA-only model and 0.849 after adding CEA; the combined result cannot be attributed solely to miRNAs [162]. Similarly, the attomolar detection limit of the experimental biosensor reported by Zhang et al. describes analytical sensitivity, whereas its training and test classification results represent separate clinical-performance assessments [163]. Overall, plasma miRNAs warrant further evaluation as potential adjunctive biomarkers within established diagnostic pathways. Their clinical utility for nodule assessment, risk stratification, monitoring and especially direct LC–PTB discrimination remains dependent on the comparator population, reference standard, assay and validation design.

### 4.4. Whole-Blood and Other Circulating miRNA Sources

Whole-blood miRNA profiles capture a different biological signal from cell-free serum or plasma. They include miRNAs from leukocytes, erythrocytes, platelets, and other circulating cells. This may increase analytical yield but complicates attribution to the tumor. Patnaik et al. developed machine-learning classifiers in 22 patients with lung adenocarcinoma and 23 high-risk or benign-nodule controls [164]. The top-scoring-pair model, based mainly on miR-630 and miR-1284, achieved 91% sensitivity and 100% specificity during leave-one-out cross-validation. Monte Carlo cross-validation yielded 86% sensitivity and 97% specificity. Both procedures constitute internal validation. The small cohort and model-development setting create a risk of overfitting, and external validation was not performed. Abdollahi et al. evaluated miR-21, miR-638, miR-148a, and miR-152 in 43 patients with NSCLC and 43 benign-lung-disease controls [165]. The combined panel achieved 96.4% sensitivity and 86.7% specificity. However, miR-152 was only borderline significant, and the panel was not tested independently. These studies reported preliminary discriminatory performance against their selected noncancer comparator groups. Their apparent performance may partly reflect systemic immune responses or differences in blood-cell composition and activity, rather than exclusively tumor-derived miRNAs.

A larger retrospective study with diverse comparator groups was conducted by Fehlmann et al., who analyzed whole-blood profiles from 3046 participants [166]. The cohort included 606 patients with lung cancer, 593 with non-tumor lung diseases, 883 with non-pulmonary diseases, and 964 unaffected controls. A machine-learning signature distinguished lung cancer from all other groups with 82.8% sensitivity and 93.5% specificity. Against non-tumor lung disease, sensitivity was 96.4% and specificity 88.6%. Early-stage sensitivity was lower at 76.3%, despite a specificity of 97.5%. The lower early-stage sensitivity is particularly relevant to the proposed detection setting. The study used diverse disease controls and was substantially larger than earlier investigations. However, its retrospective design and internal split-sample validation limit conclusions about transferability to independent clinical populations. Healthy-control comparisons and comparisons with non-tumor pulmonary disease should also be interpreted separately; neither establishes LC–PTB discrimination without explicit inclusion and analysis of PTB. Histology-specific signals have also been reported. Singh et al. found different expression patterns for adenocarcinoma and SCC, including increased miR-2114 and miR-449c in adenocarcinoma and increased miR-2115 in SCC [167]. Formal ROC analysis was not performed, so these findings remain exploratory expression associations rather than validated evidence of subtype classification.

Evidence from other blood-derived sources is more preliminary. A pooled analysis found reduced let-7c-5p expression across six public datasets, with a standardized mean difference of −0.425 [168]. However, heterogeneity was substantial (I^2^ = 70.3%); combining peripheral blood, blood-cell, serum and plasma datasets may have contributed to this variability. The result therefore cannot be assigned specifically to whole blood or interpreted as a validated diagnostic test. Hücker et al. demonstrated the feasibility of single-cell miRNA sequencing in 67 circulating tumor cells from seven patients with SCLC [169]. A median of 68 miRNAs was detected per cell, but no cancer–control classifier or diagnostic accuracy estimates were evaluated. This study supports analytical feasibility rather than clinical diagnosis. Overall, whole-blood profiles, mixed-matrix expression analyses and circulating-tumor-cell sequencing provide different types of evidence and should not be treated as interchangeable diagnostic approaches. Whole-blood signatures remain potential adjunctive biomarkers under evaluation, and their measured signals may depend on circulating blood-cell composition and systemic responses. Independent validation in clinically relevant disease–control populations is required to establish their diagnostic contribution, including direct LC–PTB discrimination where PTB is explicitly represented.

## 5. Circulating miRNAs Associated with PTB

### 5.1. Clinical Evidence for Circulating miRNAs in PTB Diagnosis and Disease Monitoring

Circulating miRNAs have been investigated as potential adjunctive biomarkers across several clinical contexts in PTB. These include detection of active disease, differentiation from LTBI, and discrimination from pneumonia or lung cancer. Studies have also examined smear-negative PTB and potential associations with drug resistance [170,171,172,173,174] (Table 6 and Table 7). These applications represent distinct diagnostic and association questions. Miotto et al. used pooled discovery samples and developed diagnostic models from only 28 PTB cases and 28 healthy controls. Model performance was assessed by leave-one-out cross-validation (LOOCV), an internal validation procedure, rather than independent validation [170]. Hu et al. enrolled 879 participants from seven centres. Their plasma signature was externally tested in a cohort containing microbiologically confirmed and clinically diagnosed PTB cases, with pneumonia, lung cancer, and healthy participants as comparator groups [174]. Four studies examined serum, while only Hu et al. evaluated plasma [170,171,172,173,174]. The studies therefore differed in recruitment, comparator selection, biological matrix, and validation design, but no reproducible PTB-specific miRNA profile has emerged.

Comparator selection is important when interpreting the reported diagnostic performance. Miotto et al. recruited participants with LTBI, extrapulmonary TB, and other pulmonary infections, but their main classifier was developed using PTB cases and healthy controls [170]. Kalayci Yuksek et al. and Wu et al. compared PTB exclusively with healthy controls [171,172]. Such comparisons evaluate PTB–healthy-control discrimination but provide limited evidence for clinical differential diagnosis. For Wu et al., incomplete reporting of participant-level culture or NAAT confirmation limits assessment of the reference standard; it does not itself demonstrate that the standard was inadequate [172]. Chu et al. reported different performance according to the comparator. Serum miR-206 distinguished PTB from healthy controls with an AUC of 0.9647 and a specificity of 91.25%. Against LTBI, the reported AUC and specificity were 0.8168 and 56.82%, respectively [173]. Hu et al. reported an external-testing AUC of 0.86 against mixed controls comprising pneumonia, lung cancer, and healthy participants [174]. This overall estimate should not be interpreted as a separate PTB–lung cancer AUC. Including symptomatic disease controls addresses a clinically relevant differential-diagnosis question, but differences in populations and reference standards prevent direct ranking against healthy-control studies.

The studies also differed in biomarker format and panel composition. Miotto et al. developed a 15-miRNA signature and separate population-specific models [170]. Expression patterns differed between European and African cohorts, and the original designation miR-223 should be retained without an unverified strand reassignment. Wu et al. evaluated a five-miRNA RT-qPCR signature and a three-miRNA Simoa panel [172]. They reported opposite expression directions for miR-99b across these platforms. Chu et al. examined miR-206 as an individual serum marker [173], whereas Hu et al. developed a nine-miRNA plasma classifier [174]. The latter contained components with different expression directions. No stable combination of miRNAs was reproduced across these models. Multivariable signatures may improve classification by integrating complementary signals. However, their performance does not establish a universal biological signature of PTB. Panels containing mixed components should not be described collectively as either upregulated or downregulated.

The strength of evidence varied substantially across validation designs. Miotto et al. assessed their 15-miRNA RVM model using LOOCV after model development. The model achieved an accuracy of 82.14%, with 85.71% sensitivity and 78.57% specificity. However, only 56 individual samples were included [170]. Kalayci Yuksek et al. did not report independent validation [171]. Wu et al. used separate RT-qPCR and Simoa phases, but both relied on comparisons with healthy controls. Separate analytical phases do not establish independent external validation. Their Simoa panel achieved an AUC of 0.818, with 83.9% sensitivity and 79.7% specificity [172]. Chu et al. recruited participants prospectively but did not independently validate the marker; prospective recruitment and external validation are distinct design features [173]. Hu et al. used fivefold cross-validation followed by independent multicentre testing. Their nine-miRNA model had a training AUC of 0.84 against pneumonia and lung cancer controls and an external-testing AUC of 0.86 against mixed pneumonia, lung cancer, and healthy controls [174]. The external cohort also included microbiologically confirmed and clinically diagnosed PTB. For the latter, diagnosis incorporated treatment response, introducing reference-standard uncertainty. These estimates cannot be compared without considering comparator groups, PTB confirmation criteria, assays, and validation design.

Analytical heterogeneity further limits comparison across studies. Miotto et al. used TaqMan arrays, preamplification, quantile normalization, and multivariable classification [170]. Kalayci Yuksek et al. normalized the same RT-qPCR data separately to U6 and miR-93. The AUC of miR-144 was 0.72 with U6 and 0.79 with miR-93. miR-125b and miR-146a were significant only when miR-93 was used [171]. Wu et al. compared conventional RT-qPCR with direct Simoa detection. miR-99b was downregulated by RT-qPCR but showed a higher concentration in PTB when measured by Simoa [172]. The reported Simoa detection limits of 0.449–1.889 fM describe analytical sensitivity, not clinical diagnostic sensitivity. Chu et al. used U6 and the comparative 2^−ΔΔCt^ method [173]. Hu et al. used direct S-Poly(T) Plus RT-qPCR and three plate-specific internal references [174]. These differences can affect signal magnitude, statistical significance, and expression direction. Assay specificity, calibration, sample handling, and differences between analytical-phase populations may contribute to discordant results. Assay-dependent findings should therefore not be interpreted as reproducible biological changes without analytical standardization.

Biological interpretation remains largely inferential. Predicted targets of the reported miRNAs involved NF-κB and NOD-like receptor signaling [171]. These bioinformatic predictions do not establish functional effects of the circulating markers. Serum miR-206 correlated with IP-10, TNF-α, IFN-γ, IL-4, and IgG. Its strongest correlation was with IP-10, with an r value of 0.7596. These associations may be consistent with systemic immune activation, but they do not establish a causal role or specificity for PTB. The reported association between miR-206 and drug resistance was also imprecise. Bacterial burden, cavitation, lesion extent, and previous treatment could have influenced the result [173]. Predictive utility cannot be inferred without establishing that miRNA measurement preceded resistance determination and independently predicted that outcome. Predicted pathways associated with the plasma panel included PI3K–AKT, MAPK, and AMPK, but these were not experimentally established mechanisms [174]. Reliable monitoring requires paired serial samples, predefined collection times, and comparison with microbiological conversion, treatment failure, or relapse. The reviewed evidence did not establish validated longitudinal monitoring performance. The current evidence therefore supports further evaluation of circulating miRNAs as potential adjunctive diagnostic or triage biomarkers, alongside microbiological testing, but does not establish clinical readiness or validated prediction of drug resistance, treatment response, or disease progression.

### 5.2. Diagnostic Performance and Clinical Stratification of Circulating miRNAs in PTB

PTB encompasses clinically distinct states rather than a single uniform phenotype. Circulating miRNAs have therefore been evaluated across active and latent infection, pulmonary and extrapulmonary disease, cavitary and non-cavitary PTB, and drug-resistant and drug-susceptible disease (Table 8 and Table 9). They have also been studied before and after treatment and in the differential diagnosis of PTB and lung cancer. Cui et al. examined cavitation and drug resistance [175]. Yareta et al. investigated expression differences between active TB, latent infection, and healthy status [176]. Adhikary et al. evaluated pulmonary and extrapulmonary TB against noninfectious respiratory-disease controls [177]. These applications represent different biological and clinical questions [178]. Rafi et al. examined active PTB, latent infection, and separate treated and untreated groups [179], while Østergaard et al. evaluated TB disease versus TB infection in discovery and validation groups [180]. Liu et al. addressed the distinct question of PTB–lung cancer discrimination in patients with similar radiographic presentations [160]. A marker of systemic inflammation in active PTB may not identify lesion cavitation, anatomical disease site, or drug resistance. Results obtained for one PTB phenotype should therefore not be generalized to all forms of the disease. Diagnostic discrimination, resistance associations, and treatment-associated expression changes should also be interpreted as separate endpoints.

Differentiation of PTB from lung cancer represents a distinct and clinically important application. Liu et al. developed a plasma panel containing miR-342-3p, miR-199a-3p, and miR-199b-3p. All three miRNAs were higher in PTB than in lung cancer [160]. The model achieved an AUC of 0.911 in training and 0.886 in internal split-sample validation. Validation sensitivity was 87.9%, while specificity was 75.9%, with PTB designated as the positive class. This comparison directly addresses the review’s differential-diagnosis question, because PTB and lung cancer can produce similar symptoms and radiographic abnormalities. However, the reported specificity indicates incomplete separation within this validation sample; performance in an unselected clinical population remains uncertain. In addition, 42 of the initially recruited PTB patients lacked microbiological confirmation and were clinically diagnosed. LC diagnosis relied on histopathological or cytological confirmation, and patients with concurrent PTB and lung cancer were not evaluated. The panel should therefore be considered a potential adjunctive biomarker whose proposed triage application requires further clinical validation. It cannot replace microbiological testing, tissue examination, or histopathological confirmation.

Circulating-miRNA profiles may vary with anatomical disease distribution, tissue damage, and treatment history. Cui et al. reported that a plasma panel containing miR-769-5p, miR-320a, and miR-22-3p distinguished PTB from healthy controls with an AUC of 0.905. However, the panel did not differentiate cavitary from non-cavitary PTB [175]. The study included discovery, training, and validation phases, but independent external validation was not established. miR-320a was further reduced in drug-resistant disease and distinguished resistant from susceptible PTB with an AUC of 0.882. This association does not establish a resistance-specific mechanism or prospective prediction of drug resistance. The groups differed in previous treatment exposure, which may have influenced miR-320a expression. Adhikary et al. reported an AUC of 0.556 for serum miR-29a in PTB versus noninfectious respiratory-disease controls. Its AUC for extrapulmonary TB versus these controls was 0.861, with reported sensitivity of 70.3% and specificity of 68.1% [177]. However, the miRNA analysis included only seven pulmonary and eight extrapulmonary TB cases and nine controls, making these findings exploratory rather than validated evidence of disease-site classification. Cavitation, extrapulmonary involvement, and drug resistance represent distinct clinical features that may be associated with differences in bacterial burden, tissue destruction, immune compartmentalization, and treatment exposure. Current evidence therefore does not establish a phenotype-specific circulating-miRNA signature.

The clinical interpretation of a circulating miRNA depends on the disease context. Yareta et al. reported higher miR-155 in latent infection than in healthy controls, without significant active-TB-specific changes among the tested candidates [176]. Rafi et al. reported increased serum miR-144-3p in newly diagnosed PTB, with an AUC of 0.960 against healthy controls; this estimate does not establish PTB–LTBI discrimination. Lower expression in their separate treated group represents a cross-sectional treatment association rather than validated longitudinal monitoring [179]. Østergaard et al. reported a PTB–TB infection AUC of 0.87 in discovery and 0.57 in a separate validation group, and treatment-associated changes were not reproduced [180]. Their overall TB analysis included pulmonary and extrapulmonary disease and definite, probable, and possible diagnoses. These differences limit interpretation of reproducibility but do not establish the absence of a biological boundary between active and latent infection. These markers were not shown to identify PTB across all clinical categories. Their reported directions may reflect inflammation, anatomical involvement, treatment exposure, or the selected alternative diagnosis. A circulating miRNA should therefore be interpreted in relation to a predefined clinical question. Current findings support further evaluation of context-dependent associations and potential adjunctive diagnostic applications, but do not establish a universal marker or validated monitoring test.

### 5.3. Longitudinal Applications of Circulating miRNAs in PTB Progression and Treatment Response

Longitudinal studies address two distinct applications of circulating miRNAs (Table 10 and Table 11). The first is monitoring biological changes after the initiation of antimycobacterial treatment. The second is evaluating the risk of progression to active PTB before clinical diagnosis. Wagh et al. examined paired treatment-associated changes and multidrug-resistance associations [181]. Wang et al. compared groups at different treatment stages [182]. Duffy et al. evaluated prediagnostic profiles in household contacts [183]. Barry et al. investigated serial changes during treatment [184], whereas Xin et al. examined baseline profiles in participants who subsequently progressed to active TB [185]. These applications should not be treated as equivalent. Baseline diagnostic AUCs also do not establish monitoring performance or prospective prediction of resistance. A marker that returns toward a reference level after therapy may reflect reduced inflammation without predicting treatment success. Conversely, a prediagnostic marker may indicate incipient disease without providing information about bacterial clearance. Longitudinal value therefore depends on the relationship between miRNA trajectories, sampling time, and the specific clinical outcome.

Treatment-associated miRNA changes differed in timing and direction. Wagh et al. found that serum miR-16 and miR-155 moved toward control levels after six months, but the paired analysis included only 10 patients [181]. Wang et al. reported that miR-21-5p, miR-92a-3p, and miR-148b-3p were elevated before treatment and decreased in participants classified as cured. In contrast, miR-125a-5p increased after two months of therapy [182]. These treatment-stage comparisons were not entirely paired. Barry et al. collected plasma before treatment and after 1, 2, and 6 months. Treatment-responsive markers, reported using the shortened labels miR-29a, miR-99b, miR-26a, and miR-146a, changed within the first month; only miR-29a and miR-99b returned to healthy-control levels by treatment completion [184]. These markers should be distinguished from the five-miRNA diagnostic panel, and shortened names should not be assumed to specify a particular strand. These patterns indicate that circulating miRNAs do not follow a uniform recovery trajectory. Individual markers may reflect changes in immune activity, inflammation, drug exposure, or tissue repair, although these mechanisms were not established by the observed trajectories.

Movement toward healthy-control levels does not establish microbiological cure. Reduced miRNA expression may reflect declining bacterial burden, but it may also result from changes in immune-cell composition, systemic inflammation, tissue injury, or drug effects. Wagh et al. assessed 10 paired samples and defined treatment success using clinical improvement and a negative AFB smear, which is not equivalent to culture-confirmed clearance [181]. In Wang et al., untreated, two-month-treated, and cured groups were not fully composed of paired participants [182]. Barry et al. included serial sampling, but only 11 treatment failures compared with 89 successes, limiting the precision and robustness of treatment-failure assessment [184]. The baseline diagnostic AUC should not be interpreted as failure-detection accuracy. These designs provide treatment-associated expression evidence, including within-patient observations in [181,184], but do not establish validated monitoring performance. Clinical utility requires evaluation against predefined outcomes such as microbiological conversion, treatment failure, or relapse, with serial culture and other clinical assessments collected at appropriate time points. Without validated relationships to these outcomes, miRNA normalization should be interpreted as evidence of treatment-associated host-response changes rather than proof of cure.

Prediagnostic studies reported performance that depended on the interval between sampling and diagnosis. Duffy et al. developed a 47-miRNA signature in household contacts who were followed for disease progression [183]. In the reported blinded validation, the signature achieved an AUC of 0.74 for samples collected within six months before diagnosis. Its AUC declined to 0.66 when samples collected up to 24 months before diagnosis were included. A reduced three-miRNA model achieved an AUC of 0.67. These findings support a prediction-window-dependent association but do not establish the underlying mechanism or stable long-term susceptibility. Interpretation of validation independence also requires participant-level separation and appropriate handling of repeated samples. Xin et al. studied a prospective cohort of 7505 participants with LTBI, of whom 117 developed active TB over five years [185]. RT-qPCR verification used participants from the same parent cohort rather than an external population. MiR-451a alone had 38.4% sensitivity and 90.9% specificity, while a model incorporating BMI and previous TB history had 80.8% sensitivity and 79.2% specificity. These combined-model results cannot be attributed solely to miR-451a or used to quantify its incremental contribution without comparison against a clinical-factor-only model.

Clinical translation requires evidence that prediagnostic measurements or within-person trajectories predict their intended clinical outcomes. Future studies need paired serial samples collected at predefined intervals before diagnosis and throughout treatment. Progression should be microbiologically confirmed, and monitoring studies should include sufficient numbers of treatment failures, relapses, and successfully treated patients. Models should report lead time, positive predictive value, and improvement over clinical risk assessment. These measures are more informative than group-level differences at isolated time points. External validation should evaluate the predefined sampling schedule and intended prediction window. A model designed to identify disease within six months should not be presented as a general long-term susceptibility test. Current evidence supports treatment-associated expression changes and time-dependent associations with subsequent TB development [181,182,183,184,185]. However, circulating miRNAs remain potential adjunctive risk or monitoring biomarkers under evaluation, alongside standard microbiological assessment, rather than validated surrogates of cure or established standalone progression tests. These studies provide context for TB progression and treatment response; they do not establish LC–PTB discrimination.

### 5.4. Age-, Population-, and Immune-Context Dependence of Circulating miRNA Profiles in PTB

The biological meaning of a PTB-associated miRNA depends on the compartment in which it is measured (Table 12 and Table 13). Zhou et al. analyzed miRNAs in peripheral blood leukocytes collected after erythrocyte lysis [186]. These measurements may reflect intracellular regulation and the composition of circulating immune cells. They do not represent cell-free circulating miRNA concentrations. This study therefore provides cell-associated context rather than direct evidence about plasma biomarkers. In contrast, Xin et al., Kathirvel et al., and Ulger et al. examined plasma [187,188,189]. Plasma miRNAs may originate from leukocytes, erythrocytes, platelets, or damaged tissues and may circulate within extracellular vesicles or other protective complexes. Their concentrations are also influenced by cellular release and systemic clearance. Consequently, leukocyte and plasma findings cannot be pooled as equivalent evidence. Opposite expression directions could reflect differences in cellular composition, release, processing, or intracellular regulation; redistribution between compartments remains a possible explanation rather than an established mechanism. They do not necessarily represent a true biological contradiction.

Differences between biological compartments were reported in separate paediatric TB studies. Zhou et al. included 28 children with TB and 24 healthy controls. However, microarray discovery was based on only three culture-confirmed cases and three controls [186]. The overall TB group included 14 culture-positive and 14 culture-negative, clinically diagnosed cases; culture negativity alone does not establish misdiagnosis. The separate RT-qPCR phase did not establish independent external classifier validation. Kathirvel et al. analyzed plasma from 30 children with active TB and 30 healthy controls [188]. The TB group included 25 pulmonary and five extrapulmonary cases. Plasma miR-21, miR-31, and miR-155 were upregulated, whereas miR-146a was downregulated. The AUC was 0.978 for miR-31, 0.953 for miR-155, and 0.903 for miR-146a. These estimates concern mixed childhood TB versus healthy controls and were not independently validated; controls were not assessed by TST or IGRA. Plasma miR-29a was not significantly increased in the detailed results (*p* = 0.16). Thus, miR-146a showed concordant downregulation in the two studies. In contrast, miR-31 and miR-155 were reduced in leukocytes but increased in plasma. This pattern could reflect cellular release, altered intracellular retention, or differences in leukocyte composition. However, these were different participant cohorts, not paired measurements of both compartments. Reported discrimination in small case–control samples does not establish matrix-independent reproducibility.

Age and population differences may also affect the transferability of candidate markers. Xin et al. examined only 34 adults, including 10 patients with smear-positive PTB, 13 individuals with LTBI, and 11 uninfected controls [187]. Microarray profiling identified 119 of 1887 miRNAs as differentially expressed between at least two groups. However, RT-qPCR confirmed only let-7b-5p and miR-30b-5p as elevated in active PTB. No selected marker distinguished LTBI from uninfected status. Confirmation occurred within the discovery population, and no ROC-based diagnostic estimates or predefined classifier were reported. These findings therefore represent expression associations rather than validated diagnostic performance. These adult candidates differed from the principal pediatric markers reported by Zhou et al. and Kathirvel et al. [186,188]. The limited overlap may reflect age-dependent immunity, ethnicity, BCG vaccination, exposure history, disease severity, or confirmation criteria. It may also result from small, single-centre designs and unstable candidate selection. The studies did not isolate the contributions of these possible factors. Therefore, markers identified in one age group or population should not be assumed to retain the same expression pattern or clinical value in another setting.

Ulger et al. examined plasma miRNAs together with inflammatory cytokines in 44 culture-confirmed PTB patients and 44 healthy controls [189]. Among 84 candidate miRNAs, 15 were upregulated and 3 were downregulated in PTB. Eleven miRNAs were associated with TNF-α, IFN-γ, IL-1β, IL-8, or IL-10. The analysis reported associations between circulating miRNA expression and inflammatory cytokines. However, correlation does not demonstrate biological regulation. The study could not determine whether the miRNAs altered cytokine production or changed in response to inflammation. Formal ROC analysis, diagnostic cutoffs, and a multivariable miRNA–cytokine model were not reported. The clinical value of combining the two biomarker classes therefore remains unknown. In addition, testing 84 candidates without clearly reported correction for multiple comparisons raises concern about false-positive associations.

Recurrence of a miRNA name across studies did not consistently indicate reproducibility. The agreement and opposing directions described above concern separate cohorts and matrices, rather than replication of the same predefined assay or classifier. Zhou et al. reported increased miR-29/miR-29b, whereas plasma miR-29a did not reach statistical significance in Kathirvel et al. [186,188]. The ambiguous miR-29/miR-29b designation should not be interpreted as equivalent to miR-29a; exact mature-miRNA identity remains necessary for comparison. Moreover, miR-29, miR-29a, and miR-29b are not interchangeable annotations. Reproducibility requires agreement in mature strand, sample compartment, expression direction, age group, and comparator population.

These findings highlight the need to consider biological and clinical context; they do not establish validated context-specific signatures or a universal PTB panel. Pediatric and adult immune responses may produce different miRNA patterns. Leukocyte measurements may reflect intracellular regulation and cell composition, whereas plasma concentrations may reflect contributions from several cellular and tissue sources. Culture-confirmed and clinically diagnosed PTB populations may also differ in bacterial burden and disease certainty. Similarly, active PTB versus LTBI and PTB versus healthy-control comparisons address different clinical questions, neither of which establishes LC–PTB discrimination. Future studies should therefore validate pediatric and adult signatures separately. They should include age-matched pulmonary-disease controls, consistent microbiological definitions, standardized plasma processing, haemolysis assessment, and exact 3p/5p annotation. Parallel measurement of cell-associated and cell-free miRNAs could clarify apparent directional conflicts. Age, population, biological compartment, and immune state should be evaluated as potential influences on performance, rather than assumed to explain observed differences. Plasma miRNAs remain potential adjunctive biomarkers under evaluation, with clinical utility requiring independent validation.

## 6. Differential miRNA Signatures Distinguishing Lung Cancer from PTB

### 6.1. Heterogeneity and Reproducibility of Circulating miRNA Signatures in Lung Cancer

**Figure 2:**
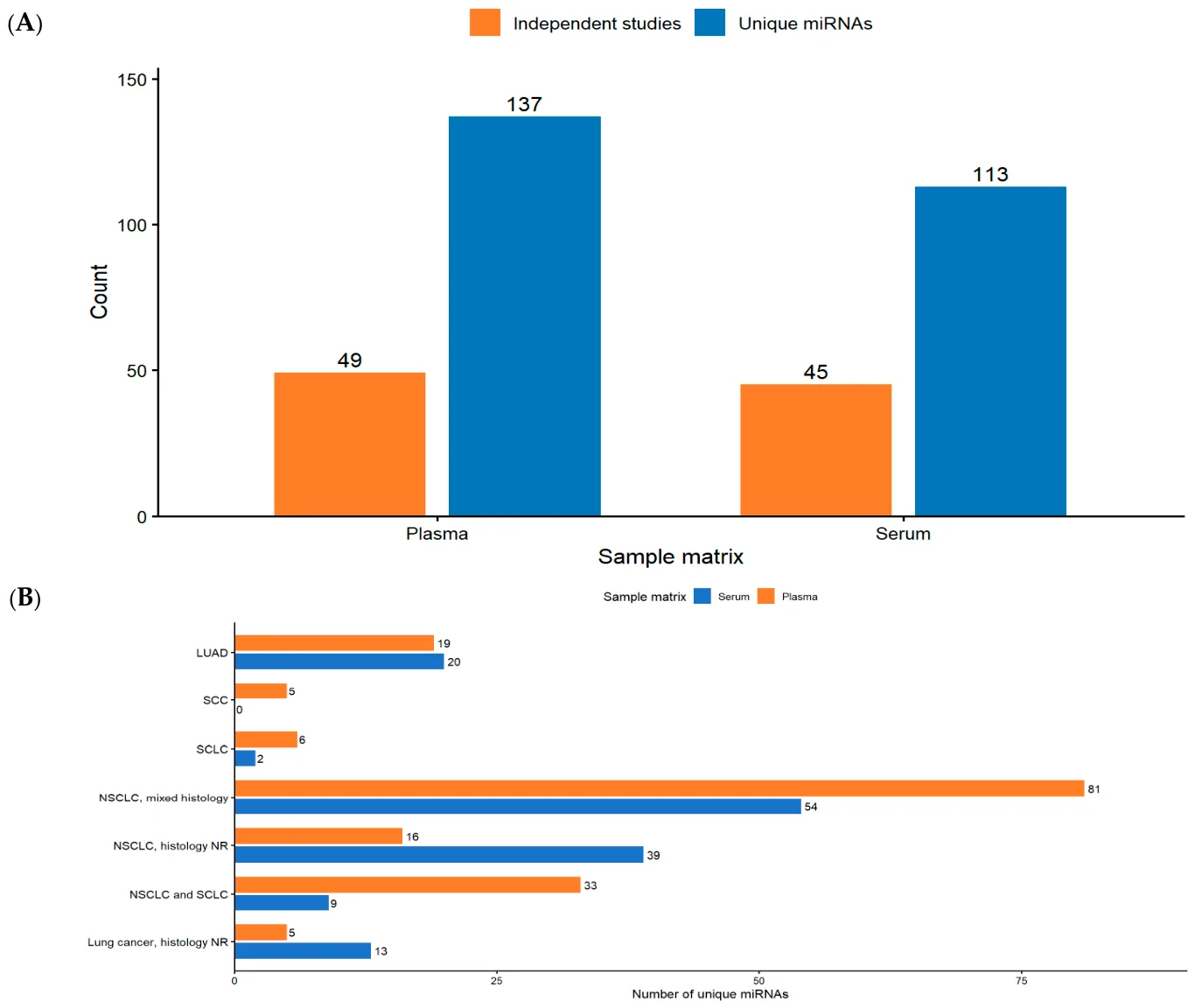
Distribution of circulating miRNA evidence across sample matrices and lung cancer categories. (**A**) Numbers of independent studies and unique miRNAs reported in plasma and serum. (**B**) Numbers of unique miRNAs reported in serum and plasma according to lung cancer type. NR, not reported; NSCLC, non-small-cell lung cancer; SCC, squamous cell carcinoma; SCLC, small-cell lung cancer; LUAD, lung adenocarcinoma.

The evidence base was distributed relatively evenly between plasma and serum. Plasma was evaluated in 49 publications, compared with 45 studies using serum. Nevertheless, plasma studies reported 137 unique miRNAs, whereas serum studies reported 113 (Figure 2A). Thus, plasma studies produced a broader candidate repertoire despite a similar volume of research [60,61,62,63,64,65,66,67,68,69,70,71,72,73,74,75,76,77,78,79,80,81,82,83,84,85,86,87,88,89,90,91,92,93,94,95,96,97,98,99,100,101,102,103,104,105,106,107,108,109,110,111,112,113,114,115,116,117,118,119,120,121,122,123,124,125,126,127,128,129,130,131,132,133,134,135,136,137,138,139,140,141,142,143,144,145,146,147,148,149,150,151,152,153,154,155,156,157,158,159,160,161,162,163]. This difference should not be interpreted as evidence that plasma provides superior diagnostic performance. The number of reported miRNAs is influenced by profiling breadth, platform selection, candidate-panel composition, normalization and reporting practices. Discovery studies using large arrays or sequencing panels also contribute more unique candidates than targeted RT-qPCR studies. These counts therefore describe the diversity of the literature, rather than the accuracy or clinical value of either matrix.

The apparent miRNA repertoire also differed markedly by lung cancer category and sample matrix. Mixed-histology NSCLC accounted for the largest number of candidates, with 81 unique plasma miRNAs and 54 serum miRNAs; the direction-specific inventory in Table 14 contains 48 and 46 distinct reported designations, respectively. NSCLC studies without reported histological classification identified 16 plasma and 39 serum miRNAs. In contrast, LUAD showed a nearly balanced distribution of 19 plasma and 20 serum miRNAs. Only five plasma miRNAs and no serum miRNAs were assigned specifically to SCC, while SCLC was represented by six plasma and two serum miRNAs (Figure 2B). The direction-specific candidates and their supporting references are presented in Table 14. These differences do not necessarily indicate greater molecular complexity in mixed NSCLC. Broad or incompletely classified cohorts permit more candidates to accumulate than narrowly defined histological groups. Pooling adenocarcinoma, SCC and other NSCLC types may also conceal subtype-specific signals. Likewise, the NSCLC–NR category cannot support histology-specific interpretation because tumor composition was not reported.

**Figure 3:**
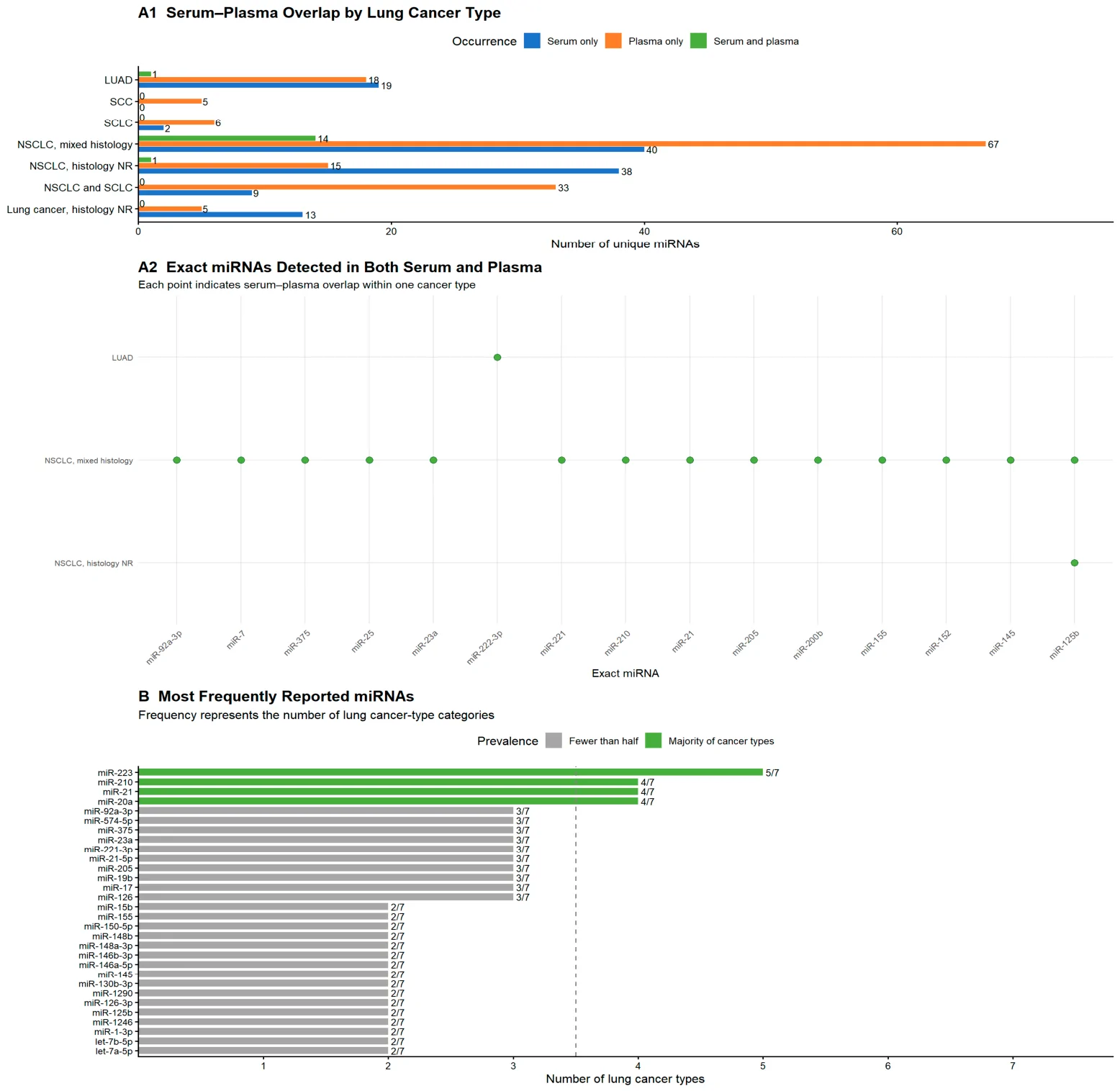
Serum–plasma overlap and recurrence of circulating miRNAs across lung cancer categories. (**A1**) Numbers of miRNAs reported exclusively in serum, exclusively in plasma, or in both matrices for each lung cancer category. (**A2**) Exact miRNAs detected in both serum and plasma within the same cancer category. (**B**) Most frequently reported miRNAs according to the number of lung cancer categories in which they were identified. The dashed line indicates miRNAs reported in at least half of the seven categories. LC, lung cancer; LUAD, lung adenocarcinoma; NR, not reported; NSCLC, non-small-cell lung cancer; SCC, squamous cell carcinoma; SCLC, small-cell lung cancer.

Overlap between serum and plasma was limited and depended on the cancer category. Only one miRNA overlapped between the two matrices in LUAD. Fourteen overlapping miRNAs were identified in mixed NSCLC, while only one was shared in NSCLC–NR. No serum–plasma overlap was observed for SCC, SCLC, combined NSCLC/SCLC or lung cancer with unreported histology (Figure 3(A1,A2)). These findings indicate that candidate miRNAs cannot be assumed to transfer directly between serum and plasma. Coagulation, platelet activation, residual cellular material and differences in RNA recovery may alter the detectable miRNA profile. However, zero observed overlap does not establish true biological matrix specificity. It may instead reflect unequal study numbers, different candidate-selection strategies and limited testing of the same miRNAs in both matrices. The larger overlap in mixed NSCLC may partly result from the much broader candidate pool available in that category rather than stronger biological concordance.

Only a small subset of miRNAs recurred across several lung cancer categories. miR-223 was reported in five of the seven categories, while miR-21, miR-210 and miR-20a each occurred in four (Figure 3B). These candidates were evaluated across multiple histological and matrix settings [64,72,77,81,88,93,107,110,111,122,125,126,127,130,133,136,145]. Several additional miRNAs occurred in three categories, but none was reported across all seven. Recurrence may indicate involvement in common tumor-associated processes, including inflammation, hypoxia, immune regulation and tissue injury. It does not, however, establish lung-cancer specificity. Frequently studied miRNAs are more likely to recur because they are repeatedly included in targeted panels. Conversely, less frequently reported candidates may simply have received less investigation. Recurrence should therefore be interpreted as evidence of repeated observation, not as proof of diagnostic validity or universal applicability.

**Figure 4:**
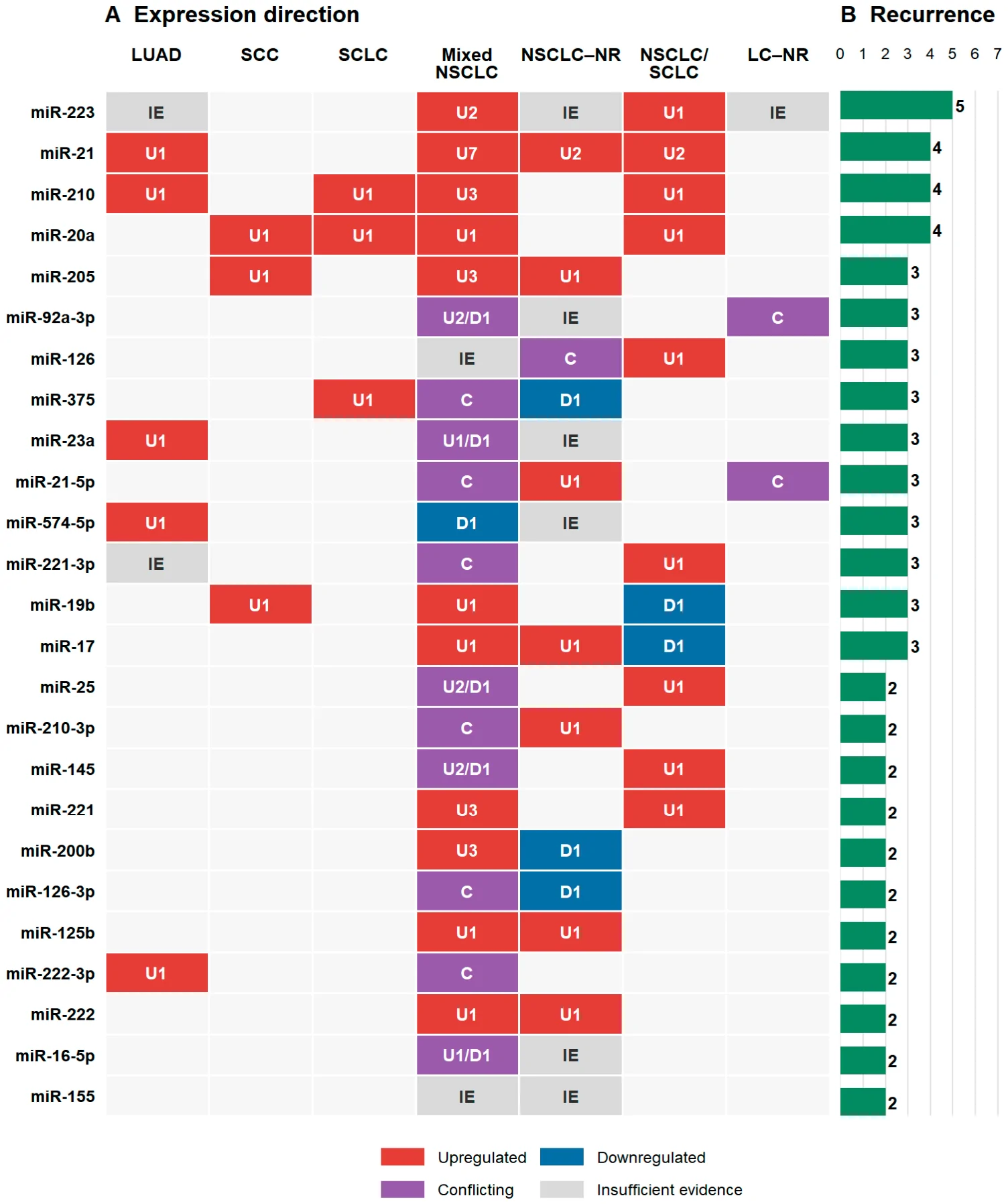
Expression-direction patterns and recurrence of circulating miRNAs across lung cancer categories. (**A**) Heatmap showing the reported expression direction of recurrent miRNAs within each lung cancer category. U*n* indicates upregulation reported in *n* independent studies; D*n* indicates downregulation reported in *n* independent studies; U*n*/D*n* indicates conflicting findings, with the respective numbers of studies reporting upregulation and downregulation; C indicates conflicting or context-dependent findings for which separate directional study counts could not be assigned; and IE indicates insufficient directional evidence. Near-white cells indicate that the corresponding miRNA was not reported in that category. (**B**) Number of lung cancer categories in which each miRNA was identified, irrespective of expression direction. Mixed NSCLC includes multiple NSCLC histological types; NSCLC–NR denotes NSCLC with histology not reported; NSCLC/SCLC denotes studies including both NSCLC and SCLC; and LC–NR denotes lung cancer with histology not reported. LC, lung cancer; LUAD, lung adenocarcinoma; NR, not reported; NSCLC, non-small-cell lung cancer; SCC, squamous cell carcinoma; SCLC, small-cell lung cancer.

Expression direction further limited cross-study reproducibility. miR-21 was predominantly upregulated across LUAD, mixed NSCLC, NSCLC–NR and combined NSCLC/SCLC studies [72,77,88,93,107,110,122,126,127,130,133,136]. miR-210 and miR-205 also showed broadly concordant increases in several categories [69,80,81,107,111,125,127,133,145]. In contrast, miR-375, miR-19b, miR-17 and miR-200b displayed category-dependent or conflicting directions. For example, miR-19b was increased in SCC and mixed NSCLC but decreased in combined NSCLC/SCLC [111,122,129]. miR-17 was increased in mixed or unclassified NSCLC but decreased in a cohort containing both NSCLC and SCLC [127,129,137]. miR-200b was increased in some mixed-NSCLC studies but decreased in NSCLC–NR [69,80,127,138]. Many heatmap cells were supported by only one study or had insufficient directional evidence (Figure 4; Table 15). Accordingly, repetition of a miRNA name does not establish a reproducible biomarker unless the mature sequence, 3p/5p strand, matrix, cancer type, comparator and direction of change are concordant.

### 6.2. Shared miRNAs Between Lung Cancer and PTB

The cross-disease comparison identified 23 exact miRNA designations shared by the lung cancer and PTB literature (Table 15). This number represents an intersection of published candidate lists. It does not measure biological similarity between the diseases. Most markers were evaluated in separate cohorts. Comparator groups also differed. Lung cancer studies commonly used healthy controls or benign pulmonary nodules. PTB studies used healthy controls, LTBI, other respiratory diseases or treated patients. The analysis additionally included serum, plasma and leukocyte measurements. These compartments are not interchangeable. Exact annotation was therefore retained. miR-21 was separated from miR-21-5p, and miR-146a from miR-146a-5p. This distinction is essential because family-level overlap may combine different mature molecules and create artificial concordance.

Several shared miRNAs changed in the same direction in both diseases. let-7b-5p, miR-133a-3p and miR-210-3p were increased in lung cancer and PTB [94,128,148,150,162,187,189]. The evidence for miR-210-3p was relatively consistent in plasma. However, concordant elevation alone does not establish differential diagnostic value. miR-21 and miR-21-5p were also frequently increased in lung cancer [72,77,88,93,107,110,122,126,127,130,133,136,143,145]. miR-21 was elevated in pediatric PTB plasma [188], while miR-21-5p increased in untreated PTB and declined after cure [182]. These findings suggest sensitivity to active pathological processes rather than disease identity. Similarly, miR-22-3p and miR-320a were reduced in both diseases [84,150,175]. Such markers may reflect shared hypoxia, inflammation or tissue injury. Their ability to distinguish PTB from lung cancer requires direct evaluation.

Opposite directions generate hypotheses for differential classification. Serum miR-206 was decreased in cohorts containing NSCLC and SCLC but increased in active PTB [109,173]. The PTB study included 88 active cases, 85 individuals with LTBI and 80 healthy controls [173]. Plasma miR-15b-5p was increased in lung cancer but decreased in 44 culture-confirmed PTB cases relative to 44 controls [159,189]. miR-10b-5p, miR-146a-5p and miR-320b also showed opposite patterns [84,109,153,172,184,189]. miR-146a was increased in lung cancer plasma but decreased in several PTB settings [127,138,171,186,188]. Nevertheless, these contrasts were inferred across independent studies. They were not estimated within the same diagnostic population. Differences in age, matrix, assay and normalization may explain part of the apparent separation. Opposite direction alone therefore does not establish discriminatory performance.

Several overlapping miRNAs were too context-dependent for simple classification. Lung cancer results for miR-16-5p, miR-92a-3p, miR-25-3p and miR-205-5p differed among studies or matrices [84,119,122,125,128,142,154,159,162]. PTB direction also changed according to disease state and sampling time. miR-16-5p was reduced before progression from LTBI and in established PTB [185,189]. However, lung cancer studies reported both increased and decreased plasma levels [142,162]. miR-31 was increased in pediatric PTB plasma but decreased in leukocytes [186,188]. This is not necessarily a biological contradiction. Intracellular retention, extracellular release and leukocyte composition can produce different directions. miR-92a-3p increased in untreated PTB and declined after cure [182]. It was increased in some lung cancer plasma studies but decreased in serum [84,142,162]. These examples show that direction must be interpreted with the matrix, disease stage and comparator. Directional summaries also do not capture effect size or uncertainty.

A panel containing miR-342-3p has been evaluated directly for PTB–lung cancer discrimination. It was decreased in one mixed-NSCLC plasma study [162]. Liu et al. then evaluated it directly in patients with radiographically similar PTB and lung cancer [160]. Their initially recruited cohort included 128 PTB cases, 109 lung cancer cases and 71 healthy controls. The panel combined miR-342-3p, miR-199a-3p and miR-199b-3p. Its AUC was 0.911 in training and 0.886 in internal split-sample validation. Validation sensitivity was 87.9%, and specificity was 75.9% [160]. Thus, approximately one quarter of lung cancer cases in the validation group could be classified incorrectly as PTB at the reported threshold. The case–control design may also overestimate performance in routine practice. Forty-two initially recruited PTB cases lacked microbiological confirmation, and concurrent PTB with lung cancer was not evaluated. The panel should therefore be considered a potential triage tool. It cannot replace microbiological or histological confirmation. Overall, the shared-miRNA analysis generates differential hypotheses, but only prospective head-to-head studies can establish clinical utility.

Direct comparative studies provide a more informative test of shared candidates than separate disease–healthy-control analyses. Abolfathi et al. measured plasma miR-155 and miR-146a in 30 patients with NSCLC, 30 with TB and 30 healthy controls [190]. Although both markers differed between each disease group and healthy controls, their AUCs for TB–NSCLC discrimination were only 0.551 and 0.513, respectively. Thus, disease-associated expression did not translate into useful differential classification in this cohort. Sun et al. examined serum miR-29a in patients with NSCLC, active PTB and concomitant NSCLC–PTB [191]. Expression was highest in PTB, intermediate in concomitant disease and lowest in NSCLC. These findings highlight the importance of including coexisting disease, but they did not establish a validated diagnostic classifier. Alimardanian et al. also evaluated plasma miR-182 and miR-34a in TB, NSCLC and healthy participants [192]. Both showed numerically lower expression in the disease groups, although the reported miR-34a difference was not statistically significant. Their disease-associated expression findings should therefore be distinguished from evidence of LC–PTB discrimination. More recently, Mohammad and Raziq reported increased serum miR-21 in active PTB compared with healthy controls [193]. Because no LC comparator was included, this provides supporting evidence of overlapping expression rather than a direct estimate of differential accuracy.

Pleural-fluid studies address a related but separate diagnostic setting: malignant versus tuberculous pleural effusion. Their results cannot be transferred directly to blood-based LC–PTB testing. Lin et al. compared extracellular-vesicle miRNA profiles in effusions associated with lung cancer, PTB and pneumonia [194]. miR-378i was elevated in the PTB group, whereas miR-205-5p and miR-200b were increased in the lung cancer group; these were exploratory expression findings requiring further diagnostic evaluation. Wang et al. subsequently identified pleural-exosomal candidates preferentially represented in lung adenocarcinoma compared with tuberculous and other benign effusions [195]. Sequencing and RT-qPCR verification supported candidate selection, but did not establish an independently validated clinical classifier. Bao et al. reported an AUC of 0.93 for a pleural-fluid panel containing miR-195-5p, miR-182-5p and miR-34a-5p when distinguishing malignant from tuberculous effusions [196]. This estimate applies to the investigated pleural-fluid comparison and requires independent testing. Zhang et al. likewise compared lung-adenocarcinoma and tuberculous pleural-exosomal profiles, with partial agreement between sequencing and RT-qPCR results [197]. Together, these studies identify candidates for a defined pleural differential diagnosis rather than demonstrate general circulating-miRNA specificity for LC or PTB.

Comparator composition and assay design also affect the interpretation of pleural diagnostic performance. Zhu et al. evaluated miR-21, miR-29c and miR-182 in serum and pleural fluid [198]. Against infection-associated effusions, which combined tuberculous and parapneumonic cases, the panel achieved AUCs of 0.832 in serum and 0.890 in pleural fluid. These are mixed-comparator estimates, not separate LC–PTB AUCs. Zhao et al. assessed pleural-exosomal miR-182-5p against a mixed benign-effusion population that included tuberculous cases [199]. The miRNA-only AUC varied with normalization, from 0.78 using cel-miR-39 to 0.80 using U6; the combined miRNA–CEA model achieved an AUC of 0.91. The combined result cannot be attributed solely to miR-182-5p, and exclusion of participants with undetectable measurements limits its applicability to an unselected diagnostic population. In contrast, Allam et al. found no significant difference in pleural-fluid miR-182 between malignant and tuberculous effusions [200]. This negative finding cautions against treating recurrence of a candidate across publications as evidence of reproducible discrimination. Moreover, miR-182 and miR-182-5p should retain their original annotations rather than be assumed to represent analytically identical measurements.

The critical review by Sheikhpour et al. summarized shared TB–NSCLC miRNAs and associated pathways, providing biological context rather than an additional primary diagnostic cohort [201]. Across the evidence considered here, LC–healthy-control and PTB–healthy-control comparisons support discovery; comparisons against benign pulmonary or other respiratory diseases address broader differential diagnosis; and direct LC–PTB comparisons address the specific question of this review. Expression associations, fitted diagnostic models and independently tested classifiers should remain distinct within each category. The additional evidence strengthens the rationale for comparative evaluation but does not establish a clinically validated LC–PTB test. Blood-based and pleural-fluid candidates remain potential adjunctive biomarkers whose utility requires prospective evaluation in the intended diagnostic population, including patients with concomitant disease, alongside microbiological and tissue-based confirmation.

## 7. Analytical and Methodological Considerations in Circulating miRNA Testing

Circulating miRNA measurements are highly sensitive to pre-analytical conditions. Blood-collection tubes, clotting time, processing delay, centrifugation and storage can alter the measured profile [202]. For EV-based assays, sample handling must also be coordinated with the isolation and characterization workflow [203,204]. Freeze–thaw cycles may cause cellular disruption or RNA degradation. Unequal sample-storage conditions were identified as a limitation in an early lung cancer study [60]. Kim et al. demonstrated that whole-blood holding time before plasma processing altered miRNA profiles in samples from healthy pregnant women [204]. Although this was not an LC or PTB population, it provides direct evidence of pre-analytical vulnerability. These variables should be recorded and standardized before patient recruitment. Serum and plasma must also be analysed separately because their miRNA compositions are not directly interchangeable.

Haemolysis and residual blood-cell contamination are major sources of measurement bias. Erythrocytes contain abundant miRNAs that can enter serum or plasma during collection and processing. Erythrocyte-associated miRNAs can therefore influence both candidate-marker measurements and normalization; haemolysis assessment is particularly important when miR-16 is used as a reference [112,187]. An apparent disease-associated change may therefore reflect sample damage rather than disease biology. Visual inspection alone is insufficient to exclude low-level haemolysis. Studies should report an objective haemolysis index and predefined sample-exclusion criteria. Residual platelets and cells should be minimized using a standardized centrifugation protocol, with the processing conditions and any assessment of residual contamination reported.

The biological fraction being analysed must be defined precisely. Unfractionated serum or plasma, EV-enriched fractions and peripheral-blood leukocytes represent different analytical preparations. EV-associated miRNAs are themselves extracellular/cell-free components, whereas leukocyte miRNAs are intracellular. Their miRNA profiles should not be treated as equivalent. For example, total serum and exosomal miR-126 produced different findings in NSCLC [90]. Precipitation-based methods produce EV-enriched preparations that may also contain non-vesicular material and do not selectively recover tumour-derived vesicles [111,203]. Leukocyte miRNAs are cellular markers and should not be classified as circulating cell-free miRNAs. Fraction-specific results require independent replication with validated isolation and characterization procedures.

RNA extraction and low target abundance introduce further variability. Extraction efficiency depends on the starting volume, carrier RNA, purification method and recovery of short RNA molecules. Synthetic spike-ins can monitor technical recovery but cannot correct for differences arising before their addition. Preamplification can improve analytical detectability, yet it may produce sequence-dependent amplification bias [170,189]. Direct-detection methods avoid some extraction and amplification steps, but they create a different analytical scale. In one PTB study, only 69.4–74.4% of measurements obtained by a single-molecule array were consistently above the detection limit [172]. These proportions describe analytical detectability, not clinical diagnostic sensitivity. Values below detection limits should therefore be reported and handled using a predefined rule rather than being silently removed. For example, excluding participants with undetectable pleural-exosomal miR-182-5p changed the population retained for diagnostic analysis [199]. The frequency and clinical distribution of such exclusions should be reported.

Normalization remains one of the most important unresolved issues. U6, miR-16, miR-93, miR-1228, global means and synthetic spike-ins have all been used as reference strategies [61,110,171,172,173,174,187]. No reference is universally stable across serum, plasma and disease states. U6 may be unsuitable for cell-free samples, while miR-16 is vulnerable to haemolysis. Reference selection can materially change statistical significance. In one PTB study, miR-125b and miR-146a were significant only after normalization to miR-93, but not to U6 [171]. Gu et al. evaluated RNU44 as an endogenous reference for plasma cell-free RNA in TB [205]. This does not establish its suitability for every circulating-miRNA assay: reference stability demonstrated in one assay and population should not be assumed to apply to other matrices, targets or disease groups. Pairwise normalization, as used in plasma-EV miRNA models, offers an alternative strategy, but ratios remain sensitive to changes in either component and require assessment of transferability [206]. Candidate reference miRNAs should therefore be selected empirically and validated in the intended matrix and population.

Analytical platforms are another source of non-comparability. Microarrays, sequencing, conventional RT-qPCR and single-molecule assays differ in sensitivity, specificity and dynamic range. Pooled discovery samples may also conceal interindividual variation [170]. Small exploratory datasets likewise provide limited information about population variability: Yang et al. performed plasma sequencing in three TB patients and three controls [207]. Ma et al. separately analysed PBMC mRNA and plasma miRNA, illustrating the need to distinguish cellular and extracellular measurements and to address normalization limitations before interpreting candidate profiles [208]. Platform dependence can even reverse the observed expression direction. miR-99b was reduced by RT-qPCR but increased when measured by direct single-molecule detection [172]. This discrepancy may involve assay specificity, calibration, sample handling or differences between analytical phases; it should not be attributed solely to disease biology. Exact sequence annotation is equally important. Mature 3p and 5p strands, precursor-level names and isomiRs should not be combined without sequence-level justification. For miR-1246, the canonical sequence and a shorter 5′-isomiR showed different expression patterns [99]. Analytical verification and clinical model validation must also be distinguished. Public-data modelling, including analyses involving repeated samples, requires separation at the participant level to avoid information leakage [209]. All samples from one participant should remain within the same training or testing partition. Feature selection, data-derived normalization parameters and cutoff optimization should be confined to training data and repeated within resampling procedures where applicable. Kim et al. separated exosomal-miRNA discovery, RT-qPCR verification and panel optimization, while their bioinformatic confirmation addressed predicted biological relationships rather than independent clinical performance [210]. Similarly, candidate verification and internal cross-validation in the plasma-EV study by Liu et al. should not be equated with external testing of the final classifier [211]. Sequencing followed by ddPCR verification, as used by Wu et al., confirms selected measurements but does not by itself establish model generalizability [212]. Chee et al. used Nanostring profiling of pleural-fluid EVs, providing a further example of platform- and matrix-specific exploratory evidence [213]. Discovery, fitted performance, internal validation and independent external testing therefore represent distinct stages of evidence.

Clinical interpretation additionally depends on patient selection, comparator spectrum, reference standards, blinding and sampling relative to diagnosis or treatment. Incomplete reporting of a reference standard should be distinguished from demonstrated inadequacy. Analytical reproducibility alone does not establish LC–PTB discrimination; clinical performance must be evaluated against explicitly defined comparators and reference standards using a prespecified assay, model and diagnostic threshold.

## 8. Prognostic and Predictive Relevance of Circulating miRNAs in Lung Cancer and Tuberculosis

Prognostic, predictive and monitoring biomarkers address different clinical questions. A prognostic marker estimates the expected disease course under defined clinical and treatment conditions. A predictive marker identifies patients more likely to benefit from a particular intervention. A monitoring marker changes in response to disease activity or treatment. These functions should not be inferred from a diagnostic case–control comparison. Prognostic and predictive evaluation requires outcome follow-up; monitoring requires repeated measurements at predefined time points. All three require clearly defined clinical outcomes. The available literature provides preliminary evidence for all three applications. However, most associations were derived from small cohorts and require independent confirmation.

Several circulating miRNAs have been associated with survival in lung cancer. Higher plasma miR-31 was associated with shorter median survival [114]. High miR-21 and miR-31 also indicated poorer survival in a heterogeneous lung cancer cohort, whereas higher let-7 was associated with longer survival [136]. Reduced serum miR-4429 was linked to poorer survival, particularly in EGFR-mutated NSCLC [96]. Serum miR-199-3p and plasma miR-126-3p were also reported as independent survival-associated markers [108,143]. Here, an independent association in multivariable analysis does not imply independent validation of a prognostic test. These findings suggest that circulating miRNAs may capture tumour burden, aggressive biology or host response. Nevertheless, many cutoffs were derived and tested in the same cohorts. Adjustment for stage, treatment and performance status was also inconsistent. The evidence therefore supports prognostic associations rather than validated prognostic tests.

Recent studies provide additional survival-associated evidence. Nan et al. reported that lower serum miR-148a-3p was associated with poorer overall survival in patients with malignant pulmonary nodules [214]. This prognostic association should be distinguished from the diagnostic performance of their combined CT–miRNA model, which was developed in a retrospective cohort without independent validation. Chen et al. reported that higher baseline serum miR-500a-3p was associated with longer progression-free survival, whereas lower expression and larger nodule diameter were adverse prognostic factors [215]. These findings concern subsequent outcomes, rather than differential benefit from a particular treatment. Neither the diagnostic model nor the accompanying cell experiments establish a clinically validated prognostic or treatment-selection test.

Serial measurements have also been investigated for postoperative surveillance. A 24-miRNA plasma classifier separated lung cancer patients into low-, intermediate- and high-risk groups with five-year survival rates of 88.9%, 79.5% and 40.1%, respectively [113]. After surgery, the risk category decreased in 76% of disease-free patients who initially had intermediate or high scores. It increased in patients who developed recurrence or progression [113]. Another four-miRNA plasma panel decreased after NSCLC resection, and larger reductions were associated with better disease-free survival [145]. These observations support postoperative surveillance as a possible application. However, the longitudinal subgroups were small. A postoperative decline may reflect removal of tumour tissue, surgical inflammation or changes in blood-cell composition. It cannot be considered a validated recurrence marker without scheduled sampling, imaging-confirmed outcomes and independent testing of a prespecified model and threshold.

Evidence for prediction of systemic treatment response remains limited. Plasma miR-21 was higher in patients with stable or progressive NSCLC than in patients who achieved a partial response to platinum-based chemotherapy [110]. This result suggests a possible association with treatment resistance. However, the response analysis included only 35 patients and was dominated by advanced disease. Other plasma miRNAs have been linked to anti-PD-1 response, but the supporting analyses combined local cohorts with public datasets [158]. A plasma and extracellular-vesicle panel containing let-7b-5p, miR-184 and miR-22-3p was also linked experimentally to osimertinib-resistance pathways [150]. Experimental involvement in resistance pathways does not establish patient-level prediction of treatment benefit. These findings remain hypothesis-generating. Predictive utility requires a treatment-specific interaction analysis. It must show that the biomarker predicts benefit from one therapy relative to an appropriate alternative.

In PTB, circulating miRNAs have mainly been evaluated as markers of treatment-associated change. Serum miR-16 and miR-155 moved toward control levels after six months of therapy, but paired samples were available from only 10 patients [181]. Wang et al. reported lower serum miR-21-5p, miR-92a-3p and miR-148b-3p in patients classified as cured under the study criteria, whereas miR-125a-5p was higher after two months of treatment; these treatment-stage groups were not fully paired [182]. Barry et al. reported paired plasma changes during therapy, with some markers returning toward healthy-control levels by treatment completion [184]. These treatment-responsive findings should be distinguished from the components and diagnostic performance of the baseline five-miRNA panel. These different trajectories may reflect bacterial clearance, immune recovery, drug exposure or tissue repair. Movement toward a healthy-control value does not itself demonstrate microbiological cure. Serial miRNA changes must be compared with culture conversion, treatment failure and relapse.

Drug-resistance studies address a separate question from longitudinal monitoring or prediction of future resistance. Zhang et al. identified plasma-exosomal miR-122-5p_R-1, miR-23b-3p_R+1 and miR-15a-5p_R-1 as candidates associated with established multidrug-resistant TB [216]. Their sequence-variant annotations should be retained, and computational target predictions should not be treated as experimentally demonstrated mechanisms. Yao and Zhang reported higher plasma-exosomal miR-122-5p in drug-resistant than drug-susceptible TB and investigated models incorporating miRNA and protein measurements [217]. They also examined targeting relationships involving KNG1 and C3 using reporter assays. Clinical expression differences, combined-model performance and experimental target evidence remain distinct findings. Neither study establishes that miRNA measurements taken before resistance develops predict its emergence or guide selection of an effective regimen.

Prospective prognostic evaluation is also being planned. The LUCEx protocol describes assessment of EV cargo, including miRNAs, in patients undergoing surgery for suspected lung cancer, with preoperative sampling and subsequent follow-up [218]. It provides a framework for investigating postoperative outcomes but reports no completed prognostic-performance or clinical-benefit results. Its planned analyses therefore should not be presented as evidence that EV-miRNA testing already detects recurrence or improves patient management.

## 9. Translational Challenges and Future Perspectives in Respiratory Molecular Diagnostics

The next phase of circulating miRNA research should move from biomarker discovery to clinical problem solving. Each test must be developed for a predefined clinical decision. Possible applications include prioritizing suspicious nodules for further investigation, distinguishing PTB from lung cancer, or directing patients toward microbiological or histological confirmation. Misdiagnosis of lung cancer as PTB can delay cancer diagnosis and expose patients to inappropriate treatment, providing a clinical rationale for direct differential-diagnostic evaluation [219]. These applications should not be combined into a single universal classifier without separate evaluation of each intended use. Their acceptable false-positive and false-negative rates are different. Future studies should therefore specify the intended user, clinical setting, target population and action resulting from the test.

Clinical development requires a phased evidence framework. Candidate selection should be followed by model development, independent validation and prospective evaluation in consecutively recruited patients. The assay and prediction model must be locked before external testing. Validation should preserve the original marker composition, coefficients and decision threshold. Performance of the original model should be reported before any recalibration; a recalibrated model requires separate evaluation. Studies should also separate technical failure from biological misclassification. Reviews of pleural-miRNA testing, integrated liquid-biopsy strategies and EV laboratory implementation identify unresolved issues concerning assay standardization, validation and clinical workflow [220,221,222]. These reviews provide supporting synthesis rather than additional independent clinical cohorts. Porcel and Sorolla similarly discuss pleural-miRNA testing within a diagnostic pathway that retains cytological assessment and pleural biopsy when indicated [223]. Pleural-fluid performance should not be extrapolated to serum or plasma testing.

Future research should evaluate clinical utility rather than discrimination alone. A statistically accurate classifier is useful only if it improves decisions or patient outcomes within the intended pathway. Studies should determine whether testing reduces avoidable biopsies, inappropriate antimicrobial treatment or delays in cancer diagnosis. Net benefit, testing burden and consequences of incorrect classification should be reported alongside conventional accuracy measures. Prospective impact trials should compare miRNA-assisted pathways with current care. Patient-centred outcomes should include time to diagnosis, procedure-related complications, treatment delay and diagnostic uncertainty. Case–control accuracy, prognostic association and treatment-associated expression changes do not themselves establish these benefits.

Integration with existing respiratory assessment is a practical strategy for further evaluation. miRNA results may be combined with imaging characteristics, smoking exposure, symptoms and microbiological findings. Such models can use different data types to represent complementary aspects of disease. However, the contribution of the molecular component must remain measurable. A combined model should be compared with the same clinical model without miRNAs [125,138,174]. Otherwise, improved performance may be driven entirely by imaging or demographic variables. Poh et al. evaluated serum-miRNA models incorporating nodule size in a case–control population [224]. Chen et al. investigated serum miR-760 combined with LDCT for malignant–benign nodule classification [225]. Their combined-strategy performance should not be attributed solely to miRNA measurements or interpreted as demonstrated screening benefit. Future models should also produce interpretable risk estimates rather than opaque binary classifications and report calibration alongside discrimination.

Recent nodule studies illustrate the distinction between model development and clinical readiness. Ji et al. reported plasma-EV miRNA classification of malignant and benign ground-glass nodules in a relatively small retrospective population [226]. Very high fitted performance in such a setting requires independent confirmation and does not itself prove either overfitting or clinical utility. Tao et al. evaluated plasma-exosomal panels through sequencing discovery, candidate identification and a separate blinded validation set [227]. These stages should remain distinct when interpreting performance. Inclusion of TB among benign lesions does not establish an LC–PTB-specific endpoint unless that comparison is separately analysed. Future differential-diagnostic studies should explicitly include PTB, other benign pulmonary diseases and concomitant LC–PTB, with appropriate microbiological and pathological reference standards.

Alternative respiratory matrices provide additional research directions but require separate assessment. Jaya et al. investigated miRNAs in exhaled breath condensate from patients with TB and healthy controls [228]. Hua et al. evaluated sputum miR-155 against a non-PTB group that included pneumonia, lung cancer and unexplained pulmonary nodules [229]. These findings concern non-blood preparations; their analytical requirements and comparator-specific performance cannot be transferred to circulating-miRNA assays. Tulab et al. examined a three-miRNA signature across healthy controls, LTBI and active TB in a cross-sectional study [230]. Such disease-state classification does not demonstrate prospective prediction of progression from LTBI or treatment response.

Greater biological resolution may help clarify candidate-marker origin and context dependence, but does not automatically improve diagnostic specificity. Cell-free miRNAs represent a mixture of signals from tumours, immune cells, blood cells and injured tissues. Future studies should determine which cells or extracellular compartments generate the selected markers. Parallel analysis of tumour tissue, granulomatous lesions, immune-cell populations and extracellular vesicles may help investigate malignant signalling and infection-related host responses [111,150,169]. Integration with proteins, microbial markers or other RNA classes may provide additional specificity. Nevertheless, multimarker models should remain biologically justified and sufficiently simple for clinical implementation, with their incremental clinical contribution tested directly.

Mechanistic studies should be interpreted separately from patient-level biomarker evidence. Sheikhpour et al. investigated nanoparticle-mediated delivery of miR-146a in lung-cancer and TB-related cell models [231], while Alimardanian et al. examined miR-182 and miR-34a in apoptosis and autophagy experiments [232]. Park et al. studied gene-expression changes following exposure of lung-cancer xenografts to tuberculous pleural-effusion-derived exosomes [233]. Kang et al. combined exploratory miRNA–mRNA network analysis with functional experiments to prioritize candidate regulatory relationships [234]. These studies provide experimental hypotheses concerning LC–TB interactions. They do not establish circulating diagnostic accuracy, prospective treatment-benefit prediction or clinical efficacy of miRNA delivery. Computational target predictions, experimentally examined relationships and human clinical outcomes therefore remain distinct levels of evidence.

Implementation must be considered during assay development rather than after validation. A clinically useful test requires an acceptable turnaround time, manageable sample volume and a format compatible with routine laboratories. Its cost should be evaluated against the procedures it is expected to prevent or prioritize. Requirements will differ between centralized cancer-screening programmes and resource-limited settings with a high PTB burden. Future studies should therefore include workflow analysis and formal health-economic evaluation of the complete testing pathway, including unsuccessful assays and downstream investigations. Access, laboratory capacity and affordability will determine whether an accurate assay produces meaningful population-level benefit.

Progress will ultimately require coordinated prospective consortia rather than additional isolated case–control studies. Shared protocols, common data elements and reference materials would allow results to be compared across centres. Individual-level data and complete model specifications should be made available for independent evaluation where consent and data-governance requirements permit. Regulatory development will also require predefined quality criteria, transparent software and post-deployment performance monitoring. Clinicians, laboratory scientists, radiologists, microbiologists and patient representatives should participate in study design. Until clinical utility is demonstrated, circulating miRNAs should remain potential adjunctive biomarkers under evaluation alongside imaging, microbiological testing and tissue-based diagnosis.

## 10. Conclusions

Circulating miRNAs are potential adjunctive biomarkers for differentiating lung cancer from PTB, but direct evidence for this application remains limited. Most published findings derive from separate disease–control comparisons, which cannot establish LC–PTB discrimination. Shared miRNA designations and opposite expression directions generate hypotheses for direct evaluation rather than demonstrate disease specificity. Recent comparative studies expand the evidence base but do not establish a clinically validated differential-diagnostic test or superiority over imaging, microbiological testing or tissue examination.

Prognostic associations, treatment-response associations and monitoring findings address different clinical questions. In lung cancer, circulating miRNAs have been associated with survival and postoperative or treatment-related changes, but these findings do not establish treatment-selection utility or validated detection of recurrence. In PTB, treatment-associated expression changes and drug-resistance associations do not demonstrate prospective prediction of resistance, treatment failure or relapse. Movement toward healthy-control levels should not be interpreted as microbiological cure.

Despite these preliminary findings, significant translational challenges must be addressed before circulating miRNA biomarkers can be widely implemented in clinical practice. The evidence is heterogeneous and predominantly based on selected case–control populations, with substantial variation in biological matrix, sample processing, normalization, analytical platform, comparator selection and reference standards. Discovery, analytical verification and internal validation must be distinguished from independent external testing. Possible cohort overlap and inconsistent miRNA annotation further limit comparisons across publications.

Future development should prioritize prospective multicentre studies in clinically relevant diagnostic populations, including patients with concomitant lung cancer and PTB. Prespecified assays, models and thresholds should be evaluated against appropriate microbiological and pathological reference standards. Combined miRNA–clinical models must demonstrate added value beyond the same clinical assessment without miRNAs. Clinical impact, affordability and workflow feasibility should be assessed before implementation. Until these requirements are met, circulating miRNAs remain exploratory adjunctive biomarkers whose clinical utility requires confirmation.

## Figures and Tables

**Table 1:** Clinical, radiological, and pathophysiological overlap between lung cancer and PTB and approaches to differential diagnosis.

Overlapping Presentation	Lung Cancer Presentation	PTB Presentation	Shared Clinical and Imaging Findings	Findings Favoring Lung Cancer	Findings Favoring PTB	Ref.
Solitary pulmonary nodule	Peripheral adenocarcinoma	Tuberculoma	Often asymptomatic; cough; smooth, lobulated, or spiculated nodule; possible FDG uptake.	Subsolid component; pleural retraction; progressive growth; older age and smoking history.	Central or laminated calcification; satellite nodules; tree-in-bud pattern; stability or regression.	[6,28,29,30]
Hilar or mediastinal lymphadenopathy	Nodal metastases	Tuberculous intrathoracic lymphadenopathy	Enlarged, potentially necrotic or rim-enhancing lymph nodes; FDG uptake; compression symptoms.	Identifiable primary lung mass; nodal distribution compatible with metastatic spread.	Necrotic lymph nodes without an identifiable primary lung mass; accompanying PTB-compatible findings.	[6,7,31,32]
Persistent consolidation	Pneumonic-type or lepidic-predominant adenocarcinoma	Consolidative or pseudotumoral PTB	Cough, dyspnea, hemoptysis; persistent consolidation with air bronchograms or ground-glass margins.	Progressive consolidation; absence of tree-in-bud nodules.	Prolonged fever and night sweats; upper-lobe predominance; peripheral tree-in-bud nodules.	[33,34]
Diffuse pulmonary micronodules	Miliary or multifocal hematogenous metastases	Miliary PTB	Dyspnea, weight loss, systemic illness; randomly distributed bilateral micronodules.	Variable nodule size; dominant mass; extrapulmonary masses or lytic bone lesions.	Relatively uniform 1–3 mm nodules; necrotic lymphadenopathy; choroidal tubercles.	[5,6,34,35]
Cavitary lesion	Cavitating SCC	Cavitary post-primary PTB	Productive cough, hemoptysis, weight loss; thick-walled cavity with surrounding consolidation or nodularity.	Nodular inner wall; adjacent solid mass; progressive enlargement; pleural invasion.	Tree-in-bud pattern; bilateral satellite nodules; previous PTB or recent exposure.	[6,7,36,37,38,39]
Central airway obstruction	Central lung cancer, including SCLC	Endobronchial tuberculosis	Cough, hemoptysis, wheezing; bronchial narrowing, atelectasis, or post-obstructive consolidation.	Discrete endobronchial mass; progressive mediastinal lymphadenopathy.	Long-segment bronchial stenosis; ulcerative mucosa; necrotic lymph nodes.	[6,7,40,41,42,43]

FDG, fluorodeoxyglucose; PTB, pulmonary tuberculosis; SCC, squamous cell carcinoma; SCLC, small cell lung cancer.

**Table 2:** Diagnostic sampling approaches and confirmatory testing for differentiating lung cancer from pulmonary tuberculosis.

Overlapping Presentation	Diagnostic Sampling Approach	Evaluation for Lung Cancer	Evaluation for PTB	Ref.
Solitary pulmonary nodule	CT comparison; CT-guided or surgical biopsy if malignancy is suspected.	Lesion histopathology.	Microbiological testing; tissue assessment when indicated.	[6,28,29,30]
Cavitary lesion	Sputum sampling; BAL if inconclusive; biopsy of solid or nodular cavity wall.	Cytology and histopathology.	AFB smear, culture, and Xpert MTB/RIF.	[6,7,31,32]
Central airway obstruction	Bronchoscopy with biopsy and BAL or washings; EBUS-TBNA for accessible nodes.	Bronchial or nodal cytology and histopathology.	AFB smear, culture, and Xpert MTB/RIF on respiratory or nodal samples.	[33,34]
Hilar or mediastinal lymphadenopathy	EBUS-TBNA; surgical biopsy if inconclusive.	Nodal cytology and histopathology.	Mycobacterial culture, Xpert MTB/RIF, and histopathology.	[5,6,34,35]
Persistent consolidation	Sputum and BAL sampling; transbronchial or CT-guided biopsy.	BAL cytology and tissue histopathology.	AFB smear, culture, and Xpert MTB/RIF.	[6,7,36,37,38,39]
Diffuse pulmonary micronodules	BAL; transbronchial biopsy or accessible extrapulmonary lesion sampling.	Cytology and histopathology of sampled lesions.	AFB smear, culture, Xpert MTB/RIF, and site-specific testing.	[6,7,31,32,40,41,42,43]

AFB, acid-fast bacilli; BAL, bronchoalveolar lavage; CT, computed tomography; EBUS-TBNA, endobronchial ultrasound-guided transbronchial needle aspiration; PTB, pulmonary tuberculosis; Xpert MTB/RIF, *Mycobacterium tuberculosis*/rifampicin resistance assay.

**Table 3:** Secondary literature and a review protocol providing context for circulating miRNA biomarkers in lung cancer.

Reference	Publication Type	Evidence Scope	Matrix/Signature	Key Findings and Limitations
Lin and Yang, 2011 [51]	Editorial	NSCLC screening studies	Serum: 34-miRNA; plasma: 13-miRNA classifiers	~80% accuracy; serum classifier independently validated. Secondary evidence only.
Qiao et al., 2020 [52]	Review protocol	Lung cancer diagnosis	Serum miR-21	Protocol only; no diagnostic outcomes.
Sestini et al., 2016 [53]	Narrative review	LDCT screening and risk stratification	Blood-based miRNA classifier + LDCT	False positives: 19.4% to 3.7%; sensitivity 87%, specificity 81%, NPV 99%. Secondary evidence.
Zhao et al., 2016 [54]	Narrative review	Diagnosis, prognosis, monitoring	Serum, plasma, EV- and HDL-associated miRNAs	Potential clinical applications; no pooled diagnostic estimates.
Hou et al., 2016 [55]	Narrative review	NSCLC detection and classification	Plasma miRNAs and panels	Individual and combined classifiers; no pooled diagnostic estimates.
Yu et al., 2018 [56]	Systematic review	Western populations; 17 studies	35 miRNAs; 19 panels	Median sensitivity/specificity: individual 78.4%/78.7%; panels 83.0%/84.9%.
Yu et al., 2019 [57]	Systematic review	East Asian populations; 46 studies	88 miRNAs; panels of 2–20 miRNAs	Three panels ≥ 90% sensitivity/specificity; two externally validated.
Kan et al., 2021 [58]	Narrative review	Early NSCLC detection	Circulating miRNAs ± LDCT	Adjunctive diagnostic potential; no new validation cohort.
Raczkowska et al., 2023 [59]	Narrative review	NSCLC diagnosis and monitoring	Serum, plasma, whole blood, EVs	Matrix-dependent findings; no pooled diagnostic estimates.

EV, extracellular vesicle; HDL, high-density lipoprotein; LDCT, low-dose computed tomography; miRNA, microRNA; NPV, negative predictive value; NSCLC, non-small cell lung cancer.

**Table 4:** Serum-based circulating miRNAs in lung cancer.

Study	Population/Comparator	miRNA/Model	Assay	Validation	Diagnostic Findings	Main Limitation
Keller et al., 2011 [60]	8 LC patients providing 23 serial samples; 6 healthy controls	Global serum miRNA profiling	Microarray	Exploratory longitudinal analysis; no classifier validation	Differences between prediagnostic, postdiagnostic and control samples; diagnostic accuracy NR	Very small cohort; unmatched controls and unequal sample storage
Lee et al., 2011 [61]	NSCLC versus healthy controls and COPD; discovery, training and validation cohorts	Serum miR-23a	Microarray → RT-qPCR	Separate training/validation cohorts; external independence not established	Adenocarcinoma versus COPD: AUC 0.896; sensitivity 75%; specificity 92.7%	Small cohorts; diagnostic analysis restricted to adenocarcinoma versus COPD
Bianchi et al., 2011 [62]	NSCLC versus cancer-free high-risk smokers; additional benign nodules and hamartomas	Serum 34-miRNA weighted classifier	RT-qPCR	Training and separate test set within COSMOS	Test AUC 0.890; sensitivity 71%; specificity 90%	Restricted screening population; no broad prospective evaluation
Hennessey et al., 2012 [63]	Training: 30 NSCLC/20 healthy controls; testing: 55/75	Serum miR-15b/miR-27b expression-difference classifier	RT-qPCR	Training and separate test cohort; external independence not established	Test sensitivity 100%; specificity 84%; AUC NR	Healthy-control comparison; possible training overfitting
Chen et al., 2012 [64]	400 NSCLC and 220 controls; stages I–IV	Serum 10-miRNA risk score	RT-qPCR	Multicenter training/validation; external independence not established	Validation AUC 0.972; sensitivity 92.5%; specificity 90%	No benign pulmonary disease comparator
Rani et al., 2013 [65]	40 adenocarcinoma cases versus 40 matched healthy controls	Eight candidates, including miR-30c-1*, miR-616* and miR-146b-3p	TLDA → RT-qPCR	Discovery and individual-marker confirmation; no independent validation	Individual AUCs approximately 0.600–0.820; sensitivity/specificity NR	Small cohort; healthy controls; inconsistent stage-associated changes
Huang et al., 2014 [66]	Male LC patients, smokers and nonsmokers; final groups 84/100/65	Serum let-7i-3p and miR-154-5p	Microarray → RT-qPCR	Discovery and sequential verification; no external validation	LC versus nonsmokers: AUC 0.892 and 0.957, respectively	Smoking-related confounding; male-only population
Wang et al., 2015 [67]	221 NSCLC, 161 healthy controls and 56 benign nodules	Serum 5-miRNA risk score	TLDA → RT-qPCR	Chinese confirmation cohorts; blinded independent American validation	American validation: sensitivity 95%, specificity 84%; benign-nodule comparison AUC 0.979	Performance varied between cohorts; no prospective screening evaluation
Zhou et al., 2015 [68]	NSCLC versus healthy controls; test cohort 87/61	Serum miR-652/miR-660; separate model adds CYFRA21-1	RT-qPCR + protein assay	Discovery, training and same-center testing	Test AUC: miRNAs 0.858; combined model 0.943, sensitivity 85.1%, specificity 93.4%	Combined-model accuracy cannot be attributed solely to miRNAs
Halvorsen et al., 2016 [69]	Discovery: NSCLC versus COPD/healthy controls; external cohort: 100 LC/58 high-risk cancer-free controls	Serum 6-miRNA panel	TLDA → RT-qPCR	Independent external validation	External AUC 0.890 overall and 0.880 for stages I–II	Adenocarcinoma-only discovery; sensitivity/specificity NR
Peng et al., 2016 [70]	NSCLC versus healthy controls; validation cohort 120/71	Serum miR-1254/miR-485-5p/miR-574-5p + lncRNA MALAT1	RT-qPCR	Training and separate validation; no external validation	Combined-RNA validation AUC 0.844; sensitivity 93.3%; specificity 73.2%	Composite RNA model; healthy-control comparison
Chu et al., 2016 [71]	87 NSCLC versus 122 controls; additional paired tissue samples	Serum miR-147	RT-qPCR	Association/prognostic analysis	Lower expression associated with disease characteristics and survival; no diagnostic performance reported	Control status incompletely characterized; no diagnostic cutoff or validation
Chen et al., 2016 [72]	30 NSCLC versus 30 healthy controls	miR-21 in serum and exhaled breath condensate	RT-qPCR	Single case–control cohort; no independent validation	Serum AUC 0.858; sensitivity 86.7%; specificity 73.3%	Very small cohort; healthy controls; biofluid-specific results
Shi et al., 2017 [73]	120 NSCLC, 45 benign lung disease and 45 healthy controls	Serum miR-22, miR-125b and miR-15b	RT-qPCR	Separate validation NR	Individual AUCs 0.725, 0.704 and 0.619, respectively	Comparator-specific estimates, cutoffs and validation details incompletely specified
Zhu et al., 2017 [74]	50 LC, 40 benign pulmonary disease and 50 healthy controls across study phases	Serum 4-miRNA signature; 10-miRNA model also assessed	PCR array → RT-qPCR	Discovery and separate validation; no external validation	Four-miRNA AUC 0.885; sensitivity 80%; specificity approximately 82.5%	Small discovery cohort; predominantly advanced disease; mixed histologies
Grimolizzi et al., 2017 [75]	45 NSCLC versus 31 healthy controls	miR-126 in total serum, serum EVs and EV-depleted serum	RT-qPCR; EV characterization	Exploratory compartment and functional analyses	Compartment-associated differences; exact diagnostic estimates NR	Age/smoking differences; uncertain cellular origin; no external validation
Fan et al., 2018 [76]	Early NSCLC versus benign nodules or pulmonary inflammation; 323 participants	Serum 5-miRNA-ratio model	RT-qPCR	Training and separate validation; no external validation	Nodules: AUC 0.87, sensitivity 70%, specificity 90%; inflammation: 0.79, 81%, 71%	Single-region recruitment; heterogeneous inflammatory controls
Wang et al., 2018 [77]	167 NSCLC versus 128 cancer-free controls; preceding pilot screen	Serum miR-21/let-7a model	RT-qPCR	Pilot screening and larger evaluation; no independent validation	AUC 0.879; sensitivity 79%; specificity 75%	Pulmonary status of controls incompletely specified; U6 normalization
Yang et al., 2019 [78]	128 NSCLC and 47 controls, including healthy participants and COPD	Serum miR-146b/miR-205/miR-29c/miR-30b panel	RT-qPCR	Training and separate testing; external independence not established	Test AUC 0.93; sensitivity 95.38%; specificity 78.13%	Small control groups; no prospective clinical evaluation
D’Antona et al., 2019 [79]	75 early NSCLC versus 111 tumor-free controls	Serum miR-223; miR-29c assessed separately	ddPCR	Training and independent blinded validation set; external setting not established	Validation AUC 0.808; sensitivity 74.3%; specificity 78.2%	Small validation cohort; no benign-nodule comparator
Zou et al., 2019 [80]	50 untreated NSCLC versus 30 healthy controls	Serum miR-182/miR-200b/miR-205 panel	RT-qPCR	Single case–control cohort; no independent validation	Early-stage AUC 0.883; sensitivity 66.2%; specificity 71.6%; overall AUC 0.781	Small cohort; healthy controls; U6 normalization
Zhao et al., 2019 [81]	60 early SCLC versus 30 healthy controls	Serum miR-20a/miR-210 + CEA/NSE	RT-qPCR + protein assays	Validation cohort NR	Combined model reportedly improved classification; exact estimates NR	Protein–miRNA composite; individual miRNA contribution unresolved
Zhang et al., 2020 [82]	Adenocarcinoma versus healthy controls; validation: 60 cases/30 controls	Serum EV-associated miR-1290	RT-qPCR	Discovery and separate validation; no external validation	AUC 0.937; sensitivity 80%; specificity 96.7%	Small single-center cohorts; EV-isolation variability
Liu et al., 2020 [83]	64 NSCLC versus 15 healthy controls	Serum EV-associated 5-miRNA biochip panel	Lipoplex biochip; RT-qPCR comparison	Platform proof of concept; no blinded external validation	Overall AUC 0.970; sensitivity 96.9%; specificity 93.3%; early-stage AUC 1.000	Very small control group; overfitting risk; platform-specific performance
Kumar et al., 2020 [84]	75 stage III–IV NSCLC versus 40 cancer-free controls	Ten serum miRNA candidates	Small-RNA sequencing → RT-qPCR	Prospective recruitment and marker verification; no external validation	Expression and stage associations; no independently validated diagnostic panel	Very small sequencing discovery set; advanced disease only
Zhang et al., 2020 [85]	276 NSCLC versus 282 healthy controls; 104 stage 0–I cases	Serum EV miR-20b-5p/miR-3187-5p; separate models add CEA or CYFRA21-1	Microarray → RT-qPCR	Four development/validation phases; external independence not established	Stage 0–I AUC: miRNAs 0.838; with CEA 0.930; with CYFRA21-1 0.928	Pooled discovery; healthy controls; combined models require separate interpretation
Zhang et al., 2022 [86]	Discovery: 20 NSCLC/5 healthy controls; validation: 78 NSCLC/44 healthy controls	Serum miR-3149 and miR-4769-3p	Microarray → RT-qPCR	Separate validation cohort; no independent external validation	AUCs: 0.830 and 0.735, respectively; two-miRNA model AUC 0.879	Small discovery cohort; healthy controls; U6 normalization; no external validation
Ying et al., 2020 [87]	744 NSCLC versus 944 healthy controls; Asian/Caucasian cohorts	Serum 5-miRNA panel	RT-qPCR	Sequential development and validation; external independence of subsets not established	Validation AUCs 0.973/0.916/0.917; sensitivity 81.3%; specificity 90.7%	Healthy-control design; possible overlapping publication
Rai et al., 2020 [88]	30 NSCLC versus 30 age-, sex- and smoking-matched controls	Serum miR-21	RT-qPCR	Single case–control cohort; no independent validation	AUC 0.84; specificity 97%; sensitivity NR	Small cohort; predominantly advanced disease; U6 normalization
Zhou et al., 2020 [89]	125 NSCLC, 40 benign lung disease and 60 healthy controls	Serum miR-942/miR-601	RT-qPCR	Public-data discovery and clinical evaluation; no external validation	Early-stage AUCs: miR-942 0.813; miR-601 0.762; combination 0.882	Small early-stage subgroup; prognostic cutoff derived in the same cohort
Asakura et al., 2020 [90]	Validation: 1358 LC versus 1970 noncancer controls	Serum miR-1268b/miR-6075 diagnostic index	Microarray	Discovery and large retrospective validation cohort	Validation AUC 0.996; sensitivity 95%; specificity 99%	Different sample-source populations; no prospective screening evaluation
Bédard et al., 2021 [91]	Evaluable serum subset: 28 early NSCLC/17 controls; additional urine samples	Serum miR-21/miR-223; separate model adds urinary metabolite	RT-qPCR + NMR	Exploratory pilot modeling; no independent validation	Serum model AUC 0.912; sensitivity 96.4%; specificity 88.2%; composite AUC 1.000	Very small modeling subsets; incomplete sample overlap; overfitting risk
Kryczka et al., 2021 [92]	31 NSCLC versus 21 healthy controls	Serum EV miR-23a/let-7i; additional candidates assessed	RT-qPCR	Single exploratory cohort; no external validation	Individual AUCs 0.744/0.733; combined AUC 0.705	Small cohort; healthy controls; heterogeneous EV fractions
Zhang et al., 2021 [93]	65 NSCLC versus 60 healthy controls	Serum miR-145/miR-21/miR-197 panel	RT-qPCR	Single retrospective cohort; no external validation	AUC 0.939; sensitivity 91.58%; specificity 79.86%	Healthy-control comparison; early-stage performance not separately reported
Ramírez-Salazar et al., 2021 [94]	71 female adenocarcinoma cases versus 91 healthy women; smoking-stratified groups	Serum miR-532-5p/miR-25-3p/miR-133a-3p	PCR array → RT-qPCR	Discovery/verification within smoking strata; no external validation	miR-532-5p overall AUC 0.745; smokers: miR-25-3p 0.779, miR-133a-3p 0.935	Small subgroups; smoking confounding; female-only population
Tulinsky et al., 2022 [95]	60 stage I–II LC versus 60 healthy controls	Thirteen serum candidates; five strongest individual markers	RT-qPCR	Prospective case–control recruitment; no independent classifier validation	Individual AUCs approximately 0.85–0.93; sensitivity/specificity generally 75–90%	Multiple candidate comparisons; no validated multivariable classifier
Ruan et al., 2022 [96]	122 NSCLC versus 72 healthy controls; EGFR-mutant versus wild-type cases	Serum miR-4429	RT-qPCR; functional assays	Single case–control cohort; no external diagnostic validation	LC versus controls: AUC 0.918, sensitivity 89.34%, specificity 84.72%; EGFR comparison AUC 0.817	Same-cohort diagnostic/prognostic analyses; healthy controls
Abu-Halima et al., 2023 [97]	240 serial serum samples involving lung, colon and breast cancer	21-miRNA profile; static and longitudinal markers	RT-qPCR; machine learning	Longitudinal sampling and internal cross-validation	Overall cancer classification: accuracy 79.9%, sensitivity 87.8%, specificity 67.3%	Estimates are not LC-specific; no external validation
Han et al., 2023 [98]	NSCLC versus healthy controls; training 45/26, validation 32/24	Serum miR-23a/miR-26b/miR-4488/novel-130 panel	Small-RNA sequencing → RT-qPCR	Discovery, training and separate validation; no external validation	Panel AUC approximately 0.901–0.910	Small regional cohort; healthy controls; limited early-stage cases
Aiso and Ueda, 2023 [99]	20 NSCLC versus 20 healthy controls	Total/canonical miR-1246 and shortened 5′-isomiR	Fragment analysis; isomiR-specific RT-qPCR	Analytical characterization; no diagnostic validation	Increased total miR-1246 mainly attributable to the isomiR; diagnostic accuracy NR	Small analytical study; isoform-specific findings
Inagaki et al., 2023 [100]	LC versus healthy controls; discovery 57/57, validation 74/74	Serum miRNA-profile machine-learning model	NGS	Discovery and separate validation; external independence not established	Validation AUC 0.98; sensitivity 89.2%; specificity 95.9%	Healthy controls; complex model; overfitting/model-selection risk
Charkiewicz et al., 2023 [101]	71 NSCLC versus 47 nonmalignant pulmonary disease controls	Serum 15-miRNA classifier	Small-RNA NGS	Internal cross-validation only	AUC 0.96 ± 0.04; sensitivity 88.4%; specificity 90.3%	Small single-center machine-learning cohort; no external validation
Raut et al., 2024 [102]	88 subsequent LC cases versus 181 cancer-free participants; prediagnostic serum	Seven-miRNA score originally developed for colorectal cancer	RT-qPCR	Prospective population-based nested study	LC risk association: OR 0.64 per SD increase; no diagnostic accuracy reported	Risk association rather than contemporaneous diagnosis; non-LC-derived score
Yang et al., 2020 [103]	744 NSCLC versus 944 controls across study phases	Serum 5-miRNA classifier	RT-qPCR	Reported validation cohorts; possible overlap with [87]	Validation AUCs 0.973/0.916/0.917; sensitivity 81.3%; specificity 90.7%	Possible overlapping report; should not count as independent corroboration
Li et al., 2024 [104]	115 LC versus 115 benign pulmonary disease controls	Serum miR-155-5p as an adjunct to LDCT	RT-qPCR + LDCT	Single clinical cohort; no external validation	Combined strategy reportedly improved classification; exact estimates NR	Combined-strategy results do not establish standalone miRNA accuracy
Salih et al., 2025 [105]	37 NSCLC versus 37 healthy controls	let-7a-5p/miR-21-3p in total serum and serum EVs	RT-qPCR; EV characterization	Exploratory compartment comparison; no external validation	Compartment-specific associations with stage/histology; no diagnostic ROC model	Small cohort; underpowered subgroup analyses
Zhao et al., 2025 [106]	Synthetic/spiked serum; no diagnostic patient cohort	Multiplex miR-21/miR-486-5p/miR-155/miR-205 sensor	Transistor-array biosensor	Analytical evaluation only	Limit of detection 2.36 fM; no clinical sensitivity/specificity	Analytical sensitivity does not establish diagnostic accuracy
Li et al., 2025 [107]	130 early adenocarcinoma versus 80 healthy controls; suspected-nodule recruitment	Serum miR-21/miR-210/miR-942 model	RT-qPCR	Internal training/test split	Test AUC 0.890; solid-nodule analysis: AUC 0.903, sensitivity 90%, specificity 80%	Incomplete benign-nodule comparator spectrum; no external validation
Serilmez et al., 2026 [108]	58 NSCLC versus 20 healthy controls	Serum miR-107; other candidates assessed separately	RT-qPCR	Single retrospective cohort; no independent validation	miR-107 AUC 0.952; sensitivity 100%; specificity 87.9%	Small unmatched archived cohort; U6 normalization
Wani et al., 2026 [109]	50 LC versus 50 matched healthy controls; predominantly advanced disease	Serum miR-221-3p; miR-146a-5p/miR-206 assessed separately	RT-qPCR	Prospective case–control recruitment; no external validation	miR-221-3p AUC 0.825; sensitivity 75%; specificity 86.7%	Advanced-disease spectrum; no established early-screening performance

* indicates the passenger strand of a miRNA duplex according to the historical miRNA nomenclature. AUC, area under the receiver operating characteristic curve; CEA, carcinoembryonic antigen; COPD, chronic obstructive pulmonary disease; CYFRA21-1, cytokeratin 19 fragment; ddPCR, droplet digital PCR; EGFR, epidermal growth factor receptor; EV, extracellular vesicle; LC, lung cancer; LDCT, low-dose computed tomography; lncRNA, long noncoding RNA; miRNA, microRNA; NGS, next-generation sequencing; NMR, nuclear magnetic resonance; NR, not specified in the available extraction; NSCLC, non-small cell lung cancer; NSE, neuron-specific enolase; OR, odds ratio; ROC, receiver operating characteristic; RT-qPCR, reverse-transcription quantitative polymerase chain reaction; SD, standard deviation; SCLC, small cell lung cancer; TLDA, TaqMan low-density array.

**Table 5:** Diagnostic performance, validation approaches, and methodological limitations of plasma-based circulating miRNA studies in lung cancer.

Study	Population/Comparator	miRNA/Model	Assay	Validation	Key Findings	Main Limitation
Wei et al., 2011 [110]	63 NSCLC versus 30 HC	Plasma miR-21	RT-qPCR	Single case–control cohort; no independent validation	AUC 0.775; sensitivity 76.2%; specificity 70.0%. Chemotherapy-response association assessed separately.	Healthy-control spectrum; small advanced-disease response subgroup; association does not establish predictive utility.
Aushev et al., 2013 [111]	32 SCC with paired surgical samples; additional 18 SCC and 6 non-NSCLC participants	Plasma and EV fraction: miR-205, miR-19a, miR-19b, miR-30b, miR-20a	LNA RT-qPCR	Discovery plus additional expression-validation set	Postoperative reductions; no diagnostic ROC performance reported.	Surgical effects and non-tumour sources remain possible; EV enrichment does not establish tumour origin.
Fortunato et al., 2014 [112]	Initial 67 participants; subsequent 69 LC and 870 disease-free MILD participants	Plasma 24-miRNA ratio classifiers; separate haemolysis classifier	RT-qPCR	Method-development and screening-cohort assessment	Sensitivity 88% and specificity 93% concern haemolysis detection, not LC diagnosis.	Cancer-detection accuracy not quantified in supplied extraction; preanalytical effects.
Sestini et al., 2015 [113]	84 LC among 3411 screened smokers; 31 patients with serial samples	Plasma 24-miRNA signature classifier (MSC)	RT-qPCR	Longitudinal screening-cohort evaluation	Five-year survival differed by MSC risk group; postoperative and progression-associated changes.	Prognosis/monitoring evidence; does not establish recurrence or residual-disease detection accuracy.
Yan et al., 2015 [114]	300 LC versus 300 HC	Plasma miR-31	RT-qPCR	Single case–control cohort	AUC 0.785; sensitivity 76.9%; specificity 74.5%.	Reported CI 0.486–0.763 does not include AUC; unresolved reporting inconsistency; no independent validation.
Powrózek et al., 2015 [115]	90 LC versus 85 HC	Plasma miR-944 and miR-3662	RT-qPCR	Case–control; no independent validation	Stage I–II NSCLC panel: AUC 0.881; sensitivity 91.7%; specificity 85.7%.	Healthy-control comparison; potential cohort overlap and risk of overfitting.
Powrózek et al., 2016 [116]	90 LC versus 85 HC	Plasma miR-448/miR-4478 model	RT-qPCR	Case–control; no independent validation	Overall AUC 0.895; sensitivity 89.4%; specificity 79.0%.	Healthy-control comparison; potential cohort overlap and risk of overfitting.
Sun et al., 2016 [117]	87 NSCLC versus 20 benign pulmonary disease and 76 HC	Plasma miR-30a	Microarray; RT-qPCR	Discovery and same-study clinical assessment	Overall AUC 0.727; sensitivity 61.0%; specificity 84.3%; benign-disease comparison assessed separately.	Control-age imbalance; no independent validation; strand not specified.
Powrózek et al., 2016 [118]	80 NSCLC versus 80 HC	Plasma miR-448, miR-944, miR-3662, miR-4478 panel	RT-qPCR	Conference abstract; no independent validation established	Operable NSCLC: AUC 0.930; sensitivity 84.8%; specificity 96.6%.	Limited methods; possible cohort overlap with [115,116].
Huang, 2016 [119]	20 NSCLC versus 10 HC	Plasma miR-148a, miR-148b, miR-152	RT-qPCR	Small expression-association cohort	Reduced expression; no diagnostic performance reported.	Exploratory association evidence; small cohort; no independent validation.
Wang et al., 2016 [120]	201 early-stage adenocarcinoma versus 178 HC; additional disease controls	Plasma miR-532, miR-628-3p, miR-425-3p risk score	TLDA; RT-qPCR	Multiphase discovery/training and blinded test sets; external independence not established here	ADC versus HC: AUC 0.974; sensitivity 91.5%; specificity 97.8%.	Reported headline accuracy does not establish discrimination from all disease controls; possible sample reuse.
Pu et al., 2016 [121]	40 NSCLC versus 5 PTB and 10 HC	Plasma histology-associated candidate signatures; components in Table S5	Microarray	Pilot discovery; no RT-qPCR or independent diagnostic validation	PTB comparator included; differential expression reported; no ROC analysis.	Very small PTB group; no validated direct LC–PTB classifier; smoking imbalance.
Zaporozhchenko et al., 2016 [122]	75 LC versus 50 HC	Plasma miR-19b/miR-183 classifier	RT-qPCR; logistic regression	Fitted case–control performance	AUC 0.990; sensitivity 94.7%; specificity 95.2%.	No stage I cases; younger HC; no independent validation; overfitting risk.
Yu et al., 2017 [123]	50 SCLC versus 30 HC	Plasma miR-92a-2-5p	RT-qPCR	Discovery and clinical assessment; no independent validation	AUC 0.761; sensitivity 56.0%; specificity 100%.	Small healthy-control cohort; smoking-related confounding.
Leng et al., 2017 [124]	Development: 92 LC/88 smoker controls; test: 34 LC/30 controls	Plasma miR-126, miR-145, miR-210, miR-205-5p panel	NGS; RT-qPCR	Separate test set; external recruitment independence requires confirmation	Development AUC 0.960; test sensitivity 91.2%; specificity 96.7%.	No benign-nodule comparator; prospective clinical utility not established.
Lin et al., 2017 [125]	Development: 69 malignant/66 benign nodules; additional cohorts n = 126 and n = 98	Plasma miRNAs plus nodule diameter	Microarray; ddPCR	Independent cohorts at additional institutions	Development AUC 0.950; external AUC approximately 0.940.	Combined imaging–miRNA model; retrospective design; clinical utility not established prospectively.
Arab et al., 2017 [126]	Training: 34 NSCLC/20 HC; validation: 72 NSCLC/50 HC	Plasma miR-141; additional candidates in Table S5	LNA profiling; RT-qPCR	Separate validation set; external independence not established here	Reported early-stage AUC approximately 0.918; sensitivity 82.7%; specificity 98.0%; metric inconsistencies retained in S5.	Healthy controls; inconsistent performance values require reconciliation with the primary report.
Xi et al., 2018 [127]	42 malignant versus 15 benign nodules	Plasma candidate miRNAs; no validated combined model	RT-qPCR	Single surgical cohort	Individual sensitivities 54.8–83.3%; specificities 60.0–86.7%.	Small selected surgical population; no independent validation.
Leng et al., 2018 [128]	56 NSCLC versus 28 smokers with benign disease	Plasma miR-205-5p/miR-210-3p classifier	FirePlex; RT-qPCR comparison	Model fitted and assessed within study cohort	AUC 0.850; sensitivity 78.6%; specificity 89.3%.	No independent clinical validation; platform-dependent measurement.
Lu et al., 2018 [129]	Five-centre cohorts: discovery 106; training 565; validation 461	Plasma six-miRNA LC classifier; separate three-miRNA histology classifier	RT-qPCR	Multiphase study; separate validation; cross-centre independence requires confirmation	LC validation AUC 0.868; sensitivity/specificity 80%/80%; histology validation AUC 0.869.	Asymptomatic risk controls; no prospective diagnostic-pathway evaluation.
Abu-Duhier et al., 2018 [130]	80 LC versus 80 HC	Plasma miR-21	RT-qPCR	Single case–control cohort	AUC 0.8913; sensitivity 80%; specificity 80%.	Predominantly advanced LC; healthy controls; no independent validation.
Cheng et al., 2018 [131]	110 LC in treatment-defined groups; 35 benign nodules and 64 HC	Plasma miR-544a	RT-qPCR	Single-centre diagnostic and treatment-associated assessment	AUC 0.841 versus HC; 0.638 versus benign nodules.	Treatment-group differences; no independent validation; weaker benign-nodule discrimination.
Zaporozhchenko et al., 2018 [132]	50 NSCLC versus 30 HC and 10 endobronchitis controls	Plasma ten-ratio classifier derived from 14 miRNAs	LNA RT-qPCR; LASSO	Fitted performance and bootstrap validation	Fitted AUC 0.979; mean bootstrap AUC 0.931.	Bootstrap is internal validation; no independent population; haemolysis and model complexity.
Wang et al., 2019 [133]	148 LC versus 148 controls	Plasma ten-miRNA panel; separate panel plus symptoms/smoking	RT-qPCR; SVM	Internal training/test split	Test AUC 0.841 for miRNAs alone and 0.976 for combined model.	Combined accuracy cannot be attributed solely to miRNAs; internal split; advanced-disease spectrum.
Liang et al., 2019 [134]	LC, benign tumours and HC; group counts NR in supplied extraction	Plasma miR-30a-5p	RT-qPCR	Validation design not specified in supplied extraction	Association/prognostic findings; exact diagnostic estimates not supplied.	Population and diagnostic evaluation insufficiently specified in supplied extraction.
Cui et al., 2019 [135]	50 NSCLC versus 50 HC	Plasma miR-23a/miR-451 panel	RT-qPCR	Single case–control cohort	AUC 0.900; sensitivity 78%; specificity 86%.	Healthy controls; no independent validation; possible blood-cell effects.
Wang et al., 2019 [136]	50 LC versus 24 HC	Plasma miR-21, miR-31 and let-7 panel	RT-qPCR	Single case–control cohort	AUC 0.798; sensitivity 72%; specificity 75%.	Mid/late-stage spectrum; no independent validation; prognostic associations separate.
Hetta et al., 2019 [137]	40 NSCLC versus 20 HC	Plasma miR-17 and miR-222	RT-qPCR	Single case–control cohort	miR-17 sensitivity/specificity 77.78%/87.5%; miR-222 50%/88.89%.	Small HC comparison; no independent validation.
Xi et al., 2019 [138]	Training: 28 NSCLC/12 benign nodules; validation: 39/13	Plasma miR-146a/miR-200b plus CT features	RT-qPCR; combined model	Same-centre temporal validation	Training AUC 0.929; temporal validation AUC 0.781.	Temporal validation is not independent external validation; small surgical cohorts.
Han et al., 2020 [139]	Discovery: 7 ADC/7 HC; validation: 56 ADC/40 HC; 51 paired surgical samples	Plasma EV miR-342-5p and miR-574-5p	NGS; RT-qPCR; ultracentrifugation	Discovery plus same-study expression/diagnostic validation	AUC 0.813; sensitivity 80%; specificity 73.2%; postoperative changes.	No independent external validation; postoperative reduction does not prove exclusively tumour origin.
Abdel Ghany et al., 2020 [140]	70 NSCLC versus 34 HC	Plasma miR-30a and miR-221	RT-qPCR	Single case–control cohort	AUCs 0.625 and 0.668; corresponding specificities 60% and 40%.	Stage II–III population; no independent validation; low specificity.
Khandelwal et al., 2020 [141]	80 NSCLC versus 80 HC	Plasma miR-590-5p	RT-qPCR; functional assays	Prospective enrolment; no independent diagnostic validation	Expression and biological/prognostic associations; no diagnostic performance supplied.	Prospective collection does not establish diagnostic validation or treatment-predictive utility.
Reis et al., 2020 [142]	Discovery: 38 NSCLC/21 HC; validation: 40/40	Plasma miR-16-5p, miR-92a-3p, miR-451a	NanoString; RT-qPCR	Separate validation cohort; external independence not established here	Sensitivity 84%; specificity 100%.	Blood-cell/haemolysis effects; healthy controls; independence requires confirmation.
Soliman et al., 2021 [143]	60 NSCLC versus 40 HC	Plasma miR-21-5p/miR-126-3p panel; CEA separately assessed	RT-qPCR; ELISA for CEA	Single cohort; fitted diagnostic assessment	Two-miRNA panel AUC 0.990; sensitivity 98.3%; specificity 95%.	Same-cohort threshold estimation; no independent validation; healthy-control spectrum.
Faversani et al., 2021 [144]	14 ADC versus 9 HC; additional mesothelioma/asbestos groups	Plasma miR-518f-3p, miR-597-5p, miR-1260a; EBC assessed separately	RT-qPCR	Prospective pilot; public-data analysis distinct from plasma clinical validation	Candidate expression findings; complete diagnostic performance not supplied.	Small pilot; public-data matrix/independence must be specified; other compartments separate.
Jiang et al., 2021 [145]	128 NSCLC versus 70 benign disease and 60 HC	Plasma miR-210, miR-1290, miR-150, miR-21-5p	RT-qPCR	Same-centre training/test cohorts	Test AUC 0.930 versus HC and 0.940 versus benign disease.	No independent external validation; postoperative/DFS association not established monitoring accuracy.
Robinson et al., 2022 [146]	18 ADC versus 18 HC; A549 experiments separately	Plasma miR-15b-3p, miR-148a-3p, miR-193b-3p, miR-195-5p	RT-qPCR	Exploratory expression and functional study	Differential expression; no diagnostic ROC performance supplied.	Small exploratory cohort; no independent diagnostic validation.
Pastorino et al., 2022 [147]	4119 high-risk participants; 119 incident LC	Plasma 24-miRNA MSC plus LDCT	RT-qPCR	Prospective single-arm BioMILD screening study	Combined strategy evaluated LC incidence/risk and screening intervals.	No randomised mortality-benefit evidence; combined pathway results are not miRNA-only accuracy.
Gargiuli et al., 2022 [148]	10 stage IV NSCLC versus 10 matched HC	Plasma 24-miRNA MSC and individual markers	Two NGS and three RT-qPCR platforms	Analytical cross-platform assessment	Variable inter-platform agreement; not independent clinical diagnostic validation.	Small advanced-disease cohort; platform-dependent expression and classification.
Zheng et al., 2022 [149]	109 indeterminate nodules; additional external cohort of 99	Plasma small-EV five-miRNA CirsEV score	NGS; RT-qPCR; LASSO	Training, internal test and external cohort	AUCs 0.920, 0.882 and 0.781, respectively.	Surgically selected nodules; generalisability and prospective utility limited.
Vadla et al., 2022 [150]	Lung-RADS 2/4 participants; approximately 20 per group	Plasma EV let-7b-5p/miR-184 and circulating miR-22-3p	NGS; RT-qPCR; logistic regression	Fitted assessment and leave-one-out cross-validation	AUC 0.924; cross-validation accuracy approximately 80%.	Small cohort; no independent validation; mixed extracellular fractions/workflows.
Jiang et al., 2022 [151]	Discovery: 10 SCLC/10 NSCLC/15 HC; validation: 15/15/9	Plasma small-EV miR-483-3p, miR-152-3p, miR-1277-5p	NGS; RT-qPCR; WGCNA	Discovery plus small same-study validation	Candidate histological discrimination; ROC estimates not supplied.	Small groups; no independent external validation.
Konoshenko et al., 2023 [152]	17 NSCLC versus 17 HC	Plasma EV/supernatant eight-miRNA, six-ratio algorithm	Stem-loop RT-PCR, as described in supplied extraction	Fitted small-cohort assessment; no independent validation	Reported sensitivity/specificity 100%/100%.	Small cohort and limited validation create substantial overfitting risk; not proof of overfitting.
Zaporozhchenko et al., 2023 [153]	Training 111 LC; validation 55 LC; SCLC versus NSCLC	Cell-free plasma miR-375-3p/miR-320b plus clinical variables; EV candidates examined separately	NanoString; RT-qPCR	Separate histological validation cohort	Validation AUC 0.833 for SCLC versus SCC and 0.793 versus NSCLC; sensitivity 37.5% at specificity 100%.	Histological classification endpoint; cohort/batch effects; limited early-stage representation.
Geng et al., 2023 [154]	NSCLC versus cancer-free smokers; counts NR in supplied extraction	Plasma miR-215-5p, miR-1299, miR-205-5p, miR-1246	NGS; LASSO	Workflow development; independent validation not established here	Overall AUC 0.801; sensitivity 66.7%; specificity 68.2%.	Platform feasibility does not establish clinical utility; cohort details incomplete here.
Zyla et al., 2024 [155]	Three LDCT cohorts: 393, 395 and 120 participants	Plasma 24-miRNA and reduced seven-miRNA classifiers	LNA RT-qPCR	Independent cross-cohort evaluation	Reduced-model AUCs 0.750, 0.530 and 0.710.	Variable transferability across cohorts; limited standalone accuracy.
Hassanin and Ramos, 2024 [156]	LUAD, SCC and HC: training 24/group; validation 12/group	Plasma EV eight-miRNA candidates	RT-qPCR	Expression-association training/validation groups	Histology/sex-associated expression; no optimised diagnostic ROC model supplied.	Commercially sourced samples; no confirmed independent diagnostic validation.
Dhilipkannah et al., 2024 [157]	Development: 58 NSCLC/62 smoker controls; validation: 56/59	Plasma miR-126-3p/miR-210-3p plus sputum bacterial DNA	ddPCR	Separate validation set; external recruitment independence requires confirmation	Combined sensitivity 87%; specificity 89%, as reported.	Performance belongs to combined plasma–sputum model; microbiome confounding.
Abdipourbozorgbaghi et al., 2024 [158]	78 NSCLC (41 LUAD, 37 LUSC) versus 44 HC	Plasma seven-miRNA LUAD and nine-miRNA LUSC panels	LNA RT-qPCR; bioinformatics	Clinical assessment plus public-data analyses; matrix-specific external validation requires confirmation	Diagnostic/prognostic and treatment-response associations; exact estimates in supplied extraction not specified.	Public tissue or other-matrix analyses must not be labelled external plasma validation; predictive utility unestablished.
Ashirbekov et al., 2024 [159]	68 LC versus 38 PTB and 41 HC	Plasma candidate miRNAs including miR-130b-3p, miR-1-3p, miR-423-5p, miR-200a-3p, miR-15b-5p, miR-18b-5p	RT-qPCR	Pooled discovery and individual candidate assessment	Direct LC–PTB expression comparisons; exact ROC estimates not supplied.	No independent validation; pooling and case–control spectrum limit translation.
Liu et al., 2025 [160]	308 recruited; 272 analysed: 110 PTB, 97 LC, 65 HC	Plasma miR-342-3p, miR-199a-3p, miR-199b-3p logistic classifier	RT-qPCR	Training 77 PTB/68 LC; internal validation 33 PTB/29 LC	Direct PTB–LC: training AUC 0.911; validation AUC 0.886, sensitivity 87.9%, specificity 75.9%.	42 recruited PTB cases lacked microbiological confirmation; mixed reference standard; no concomitant LC–PTB assessment.
Dama et al., 2025 [161]	Polish/Italian cohorts: 276 LC and 451 noncancer participants	Plasma nine-miRNA classifier	RT-qPCR; cross-cohort analyses	Correspondence; cross-cohort evaluation	AUC 0.780/0.750; stage I 0.760/0.690; benign comparator AUC 0.710.	Incomplete panel/method details in supplied extraction; no prospective clinical utility established.
Samejima et al., 2026 [162]	261 LC versus 264 controls; training 101/132, validation 160/132	Plasma 12-miRNA panel; separate miRNA plus CEA model	RT-qPCR; ridge regression	Prospective multicentre collection; training/validation cohorts	miRNA-only AUC 0.770/0.779; combined AUC 0.862/0.849 (training/validation).	Mostly self-reported healthy controls; Japanese population; early-stage utility limited; external independence requires confirmation.
Zhang et al., 2026 [163]	RT-qPCR pilot 20/20; biosensor 140 LC/80 HC	Plasma miR-203b, miR-450b, miR-642a multiplex model	RT-qPCR; CRISPR/Cas13a ECL biosensor; machine learning	Training and internal testing; no independent external validation	Training accuracy 100%; test accuracy 92.73%, sensitivity 91.83%, specificity 96.08%. Analytical limit reported separately.	Attomolar analytical detection is not clinical diagnostic accuracy; mostly advanced LC; HC comparator.

AUC, area under the receiver operating characteristic curve; ADC/LUAD, lung adenocarcinoma; CEA, carcinoembryonic antigen; CI, confidence interval; ddPCR, droplet digital PCR; EBC, exhaled breath condensate; ECL, electrochemiluminescence; EV, extracellular vesicle; HC, healthy controls; LC, lung cancer; LDCT, low-dose CT; LNA, locked nucleic acid; MSC, miRNA signature classifier; NGS, next-generation sequencing; NSCLC, non-small cell LC; PTB, pulmonary tuberculosis; RT-qPCR, reverse-transcription quantitative PCR; SCC/LUSC, squamous cell carcinoma; SCLC, small cell LC. NR in supplied extraction does not mean not reported in the primary article. EV-associated miRNAs are extracellular/cell-free components but are distinguished from unenriched plasma. Ratio/risk-score models have no single expression direction. LC–HC and LC–benign comparisons do not establish LC–PTB discrimination. Discovery, internal validation, temporal validation, external validation and prospective evaluation are distinct. Detailed extraction retained in Table S5; unresolved numerical discrepancies are explicitly flagged.

**Table 6:** Study populations, reference standards and miRNA models evaluated in PTB.

Study	Design and Population/Comparator	PTB Reference Standard	Matrix	miRNA/Model and Expression
Miotto et al., 2013 [170]	Multicentre exploratory study. Recruited cohorts included PTB, healthy controls, LTBI, other pulmonary infections, extrapulmonary TB and PTB/HIV. Diagnostic modelling: 28 PTB versus 28 healthy controls.	Positive sputum smear, *M. tuberculosis* culture or Xpert MTB/RIF.	Serum	Fifteen-miRNA signature; population-specific models also developed. Mixed, population-dependent directions; pooled and individual results were not always concordant.
Kalayci Yuksek et al., 2021 [171]	Single-centre case–control study: 20 PTB versus 20 healthy controls.	Compatible clinical presentation and positive *M. tuberculosis* culture.	Serum	Eleven candidates. miR-144 decreased with both U6 and miR-93 normalization; miR-125b and miR-146a decreased significantly only with miR-93.
Wu et al., 2024 [172]	Multiphase case–control study: 184 PTB versus 178 healthy controls across screening, RT-qPCR and Simoa phases.	Clinical presentation, chest radiography and sputum microscopy; complete participant-level culture/NAAT confirmation was not documented in the supplied extraction.	Serum	RT-qPCR: miR-101, miR-196b, miR-29a, miR-320b and miR-99b. Simoa: miR-29a, miR-99b and miR-320b. miR-99b direction differed between platforms.
Chu et al., 2025 [173]	Prospective single-centre observational study: 88 PTB, 85 LTBI and 80 healthy controls. Drug-resistance associations assessed within PTB.	Clinical/radiological assessment and positive *M. tuberculosis* culture; resistance assessed by culture-based susceptibility testing.	Serum	miR-206 increased in PTB versus LTBI/healthy controls and in resistant versus sensitive PTB. Resistance association does not establish predictive utility.
Hu et al., 2026 [174]	Prospective seven-centre study, 879 participants. Training: 185 confirmed PTB, 168 pneumonia, 57 LC. External testing: 109 confirmed PTB, 133 clinically diagnosed PTB, 60 pneumonia, 64 LC, 103 healthy controls.	Confirmed PTB: positive culture or NAAT in sputum/BALF. Unconfirmed PTB: compatible clinical/radiological findings, negative microbiology and improvement with treatment.	EDTA plasma	Nine-miRNA classifier: miR-197-3p, miR-143-3p, miR-193a-3p, miR-223-3p, miR-92b-3p, miR-320d, miR-1290, let-7d-3p and miR-16-2-3p. Mixed multivariable profile.

The [170] signature comprised let-7e, miR-146a, miR-148a, miR-16, miR-192, miR-193a-5p, miR-25, miR-365, miR-451, miR-532-5p, miR-590-5p, miR-660, miR-885-5p, miR-223* and miR-30e. Original names and historical asterisk notation are retained without assigning unverified strand identities. Mixed panels have no single expression direction. BALF, bronchoalveolar lavage fluid; EDTA, ethylenediaminetetraacetic acid; HIV, human immunodeficiency virus; LC, lung cancer; LTBI, latent tuberculosis infection; NAAT, nucleic acid amplification test; PTB, pulmonary tuberculosis; RT-qPCR, reverse-transcription quantitative polymerase chain reaction; Simoa, single-molecule array.

**Table 7:** Validation, reported performance and principal limitations of circulating-miRNA studies in PTB.

Study	Assay/Normalization	Validation	Key Findings	Main Limitations
Miotto et al., 2013 [170]	TaqMan arrays; quantile normalization; RVM and logistic models.	Pooled discovery followed by individual-sample modelling; LOOCV internal validation. No independent test cohort.	PTB–healthy controls: RVM sensitivity 85.71%, specificity 78.57%, accuracy 82.14%. Logistic model: 71.43%, 82.14% and 76.79%, respectively.	Only 56 modelling samples; pooled discovery; small population-specific groups; heterogeneous expression patterns. Broad recruitment does not establish validated discrimination against every recruited disease group.
Kalayci Yuksek et al., 2021 [171]	RT-qPCR; U6 or miR-93 normalization.	Single exploratory cohort; no independent validation.	PTB–healthy controls: miR-144 AUC 0.72 with U6 and 0.79 with miR-93. Sensitivity/specificity not supplied.	Small sample; healthy controls only; significance depended on normalization. Target-pathway predictions were not experimentally validated.
Wu et al., 2024 [172]	RT-qPCR with cel-miR-39; direct Simoa detection.	Separate screening, RT-qPCR and Simoa phases; independent external validation not established by phase separation alone.	Simoa panel, PTB–healthy controls: AUC 0.818, sensitivity 83.9%, specificity 79.7%. Analytical detection limits: 0.449–1.889 fM; distinct from clinical accuracy.	Healthy controls only; reference-standard reporting incomplete; platform-dependent miR-99b direction. Only 69.4–74.4% of Simoa samples consistently exceeded detection limits.
Chu et al., 2025 [173]	RT-qPCR; U6; 2^−ΔΔCt^ calculation.	Prospective observational recruitment; no independent diagnostic validation.	PTB–healthy controls: AUC 0.9647, sensitivity 86.36%, specificity 91.25%. PTB–LTBI: AUC 0.8168, reported sensitivity 94.12%, specificity 56.82%. Drug-resistance association was imprecise; cytokine correlations do not establish causality.	Low reported specificity against LTBI; U6 normalization concern; resistance analysis susceptible to confounding and multicollinearity. Measurement timing relative to resistance determination must be established before claiming prediction.
Hu et al., 2026 [174]	Direct S-Poly(T) Plus RT-qPCR; plate-specific references miR-572, miR-375 and miR-151a-5p.	Model development and fivefold internal cross-validation followed by independent multicentre external testing.	Training, PTB versus pneumonia/LC: AUC 0.84, sensitivity 80%, specificity 72%. External testing against mixed non-PTB comparators: AUC 0.86, sensitivity 76%, specificity 80%. Reported subgroup AUCs: confirmed PTB 0.89; unconfirmed PTB 0.83; smear-negative confirmed PTB 0.83.	Overall mixed-comparator AUC is not a separate PTB–LC estimate. Clinically diagnosed PTB uses a different reference standard. Children, pregnant individuals and patients with HIV/immunosuppression were excluded. Proposed adjunctive triage use remains subject to clinical implementation evaluation.

Performance estimates apply to the stated assay, cohort and comparator; they should not be interpreted as direct comparisons between studies. Prospective recruitment and external validation are distinct design features. Diagnostic findings, resistance associations, cytokine correlations and analytical detection limits address different questions. Bioinformatic pathway predictions do not establish experimentally demonstrated mechanisms. AUC, area under the receiver operating characteristic curve; fM, femtomolar; LOOCV, leave-one-out cross-validation; RVM, relevance vector machine; U6, U6 small nuclear RNA.

**Table 8:** Clinical characteristics and circulating-miRNA expression patterns in studies evaluating PTB diagnosis and clinical stratification.

Study	Design and Population/Comparator	TB Reference Standard	Matrix	miRNA/Model and Expression
Cui et al., 2017 [175]	Multiphase case–control study: 178 PTB and 95 healthy controls overall. RT-qPCR subset: 64 cavitary PTB, 64 non-cavitary PTB and 64 controls. Resistance comparison: 28 resistant versus 33 susceptible cases.	Sputum culture and radiography were reported; detailed participant-level bacteriological criteria require clarification.	Plasma	miR-769-5p, miR-320a and miR-22-3p: downregulated versus healthy controls. miR-320a was further reduced in resistant disease.
Yareta et al., 2020 [176]	Retrospective exploratory study: 9 active TB, 10 LTBI and 9 healthy controls.	Active TB: symptoms, abnormal radiography and positive smear and/or culture. LTBI: positive TST using PPD, without radiographic evidence of active disease.	Serum	miR-21, miR-29a, miR-99b and miR-155: no significant active-TB-specific changes. miR-155 was higher in LTBI than in healthy controls.
Adhikary et al., 2025 [177]	Prospective pilot: 17 PTB, 13 extrapulmonary TB and 16 noninfectious respiratory-disease controls. miRNA subset: 7 PTB, 8 extrapulmonary TB and 9 controls.	PTB assessment included radiography, AFB smear, Xpert MTB/RIF and line-probe assay. Extrapulmonary TB used clinical, histological, cytological and/or Xpert findings.	Serum	miR-29a, assessed alongside IL-2 and LAM: no significant PTB–control difference; higher expression in extrapulmonary TB. These were separate biomarker measurements, not a validated combined model.
Rafi et al., 2025 [179]	Cross-sectional study: 20 newly diagnosed PTB, 20 treated PTB, 20 LTBI, 20 healthy controls and 10 respiratory-disease controls. Treated and untreated participants were separate groups.	Participant-level microbiological confirmation was insufficiently described. LTBI classification used TST.	Serum	miR-144-3p: higher in newly diagnosed PTB and lower in treated PTB; no significant elevation in LTBI.
Østergaard et al., 2025 [180]	Discovery: 18 TB versus 18 TB infection. Separate validation group: 37 TB versus 22 TB infection. TB included pulmonary and extrapulmonary disease.	Definite TB: culture, TB-specific PCR or AFB microscopy. Probable/possible TB: clinical, radiological or histological findings and treatment response. TB infection: positive IGRA without active disease.	Plasma	miR-148a-3p and miR-204-5p increased; miR-584-5p decreased in discovery. These changes were not reproduced in validation; the panel has mixed expression directions.
Liu et al., 2025 [160]	LC–PTB case–control comparison. Diagnostic modelling included 110 PTB and 97 LC: training, 77/68; internal validation, 33/29.	PTB: microbiological confirmation or clinical diagnosis; 42 of the initially recruited PTB cases lacked microbiological confirmation. LC: histopathological/cytological diagnosis.	Plasma	miR-342-3p, miR-199a-3p and miR-199b-3p panel; all higher in PTB than in LC.

AFB, acid-fast bacilli; IGRA, interferon-gamma release assay; IL-2, interleukin 2; LAM, lipoarabinomannan; LC, lung cancer; LTBI, latent tuberculosis infection; miRNA, microRNA; PCR, polymerase chain reaction; PPD, purified protein derivative; PTB, pulmonary tuberculosis; RT-qPCR, reverse-transcription quantitative polymerase chain reaction; TB, tuberculosis; TST, tuberculin skin test. PPD is the reagent used for TST, not a separate testing method.

**Table 9:** Assays, validation, reported performance and principal limitations of circulating-miRNA studies in TB.

Study	Assay/Normalization	Validation	Comparator-Specific Findings	Main Limitations
Cui et al., 2017 [175]	Sequencing discovery followed by hydrolysis-probe RT-qPCR; let-7d-5p normalization.	Discovery, training and validation phases; independent external validation was not established.	PTB versus healthy controls: panel AUC 0.905. Cavitary versus non-cavitary PTB: no discrimination. Resistant versus susceptible TB: miR-320a AUC 0.882.	Pooled discovery; healthy-control diagnostic comparison; single-region recruitment. Previous treatment may confound resistance-associated expression. The resistance comparison does not establish prospective prediction.
Yareta et al., 2020 [176]	TaqMan RT-qPCR; RNU48 normalization.	Exploratory expression analysis; no diagnostic classifier or independent validation reported.	LTBI versus healthy controls: miR-155 increased, *p* = 0.032. No ROC-based diagnostic performance reported for active TB–LTBI discrimination.	Very small groups; archived serum; TST-defined LTBI; no non-TB disease controls. Expression association is not validated diagnostic accuracy.
Adhikary et al., 2025 [177]	TaqMan RT-qPCR; RNU6B normalization. IL-2 and LAM measured separately by ELISA.	Prospective recruitment; no independent model validation.	PTB versus respiratory-disease controls: miR-29a AUC 0.556. Extrapulmonary TB versus controls: AUC 0.861; reported sensitivity 70.3%, specificity 68.1%.	Exploratory findings from a very small miRNA subset; heterogeneous extrapulmonary disease; single centre; normalization uncertainty. These estimates do not establish direct PTB–extrapulmonary TB discrimination or a validated combined biomarker model.
Liu et al., 2025 [160]	RT-qPCR; three-miRNA classifier.	Training followed by internal split-sample validation, not independent external validation.	PTB versus LC, with PTB as the positive class: training AUC 0.911; validation AUC 0.886, sensitivity 87.9%, specificity 75.9%.	Case–control design; 42 initially recruited PTB cases lacked microbiological confirmation; concomitant LC–PTB was not evaluated. Potential adjunctive application requires further clinical validation.
Rafi et al., 2025 [179]	TaqMan Advanced RT-qPCR; miR-93-5p endogenous reference.	Single-study evaluation; no independent external validation. Treatment comparison was cross-sectional.	Active PTB versus healthy controls: AUC 0.960, sensitivity 90%, specificity 95%; cutoff 2.49. LTBI versus healthy controls: AUC 0.595. Treated versus untreated PTB: lower expression in treated participants, *p* = 0.001.	Small groups; incompletely described microbiological confirmation and treatment timing. The AUC 0.960 does not describe PTB–LTBI discrimination. Separate treatment groups do not establish longitudinal monitoring performance.
Østergaard et al., 2025 [180]	TaqMan profiling; exogenous controls and geNorm-selected endogenous references.	Discovery and separate validation groups; independence should be established before describing validation as external.	Mixed pulmonary/extrapulmonary TB versus TB infection: discovery AUC 0.81, sensitivity 67%, specificity 94%; validation AUC 0.55, sensitivity 19%, specificity 91%. PTB subgroup versus TB infection: AUC 0.87 in discovery and 0.57 in validation. Treatment-associated changes were not reproduced.	Small and compositionally different groups; mixed disease sites and reference standards; non-random discovery selection; retrospectively selected cutoffs; no non-TB inflammatory controls. Discovery performance was not reproduced in the validation group.

AUC, area under the receiver operating characteristic curve; ELISA, enzyme-linked immunosorbent assay; LC, lung cancer; LTBI, latent tuberculosis infection; PTB, pulmonary tuberculosis; ROC, receiver operating characteristic; RT-qPCR, reverse-transcription quantitative polymerase chain reaction; TB, tuberculosis.

**Table 10:** Study populations, reference standards and sampling schedules for circulating-miRNA studies of TB progression and treatment-associated changes.

Study	Design and Population/Comparator	TB Reference Standard/Outcome Definition	Matrix and Sampling	miRNA/Model and Expression
Wagh et al., 2017 [181]	Case–control study: 30 untreated PTB, 19 multidrug-resistant PTB and 30 healthy controls; paired treatment analysis in 10 successfully treated patients.	Untreated PTB: positive sputum AFB smear with radiological support. Resistance: line-probe assay. Treatment success: clinical improvement and negative AFB smear after six months; this does not establish culture-confirmed clearance.	Serum; paired samples before treatment and after six months in 10 patients.	miR-16, miR-29a, miR-125b and miR-155. Untreated PTB versus healthy controls: miR-16 increased, miR-155 decreased; miR-29a and miR-125b unchanged. Both miR-16 and miR-155 were lower in multidrug-resistant PTB versus healthy controls.
Wang et al., 2018 [182]	Prospectively sampled treatment-stage comparison: 131 untreated PTB, 91 two-month-treated PTB, 59 classified as cured and 131 healthy controls overall. RT-qPCR analysis: 53 participants per group.	Microbiological, pathological or clinical–radiological diagnostic criteria. Cure assessment incorporated treatment response, imaging and sputum findings; uniform culture-confirmed cure was not established by the supplied description.	Serum; untreated, two-month-treated and cured groups, not entirely paired.	miR-21-5p, miR-92a-3p, miR-148b-3p and miR-125a-5p model. The first three increased in untreated PTB versus controls and decreased in the cured group; miR-125a-5p increased after two months. The model has mixed treatment-associated directions.
Duffy et al., 2018 [183]	Prospective household-contact cohort in South Africa and Uganda: 43 and 11 progressors, respectively, matched to non-progressors. Discovery: 151 serum samples; validation: 120 samples, including repeated observations. Sample counts are not participant counts.	Incident intrathoracic TB supported by culture or AFB smear with additional radiographic/microbiological evidence.	Serum; enrolment and follow-up samples collected before diagnosis; prediction windows up to six and 24 months.	47-miRNA risk signature; dominant features included miR-148b-3p, miR-21-5p and miR-484. Mixed component directions; no single panel-wide up/down designation.
Barry et al., 2018 [184]	Exploratory set: 19 PTB/14 healthy controls. Confirmation set: 100 PTB/100 controls; repeated treatment sampling. Outcomes: 89 successes and 11 failures.	Microbiologically supported or clinically diagnosed PTB. Success incorporated microbiological conversion and/or clinical–radiological resolution; the exact failure definition requires clarification.	Plasma; before treatment and at months 1, 2 and 6.	Five-miRNA diagnostic panel: miR-146a-5p, miR-99b-5p, miR-28-5p, miR-221-3p and miR-29a-5p. Other baseline-associated and treatment-responsive markers were evaluated separately; their findings should not be attributed to every panel component.
Xin et al., 2022 [185]	Nested case–control study within a five-year cohort of 7505 participants with positive IGRA or TST; 117 developed TB. Discovery: 20 progressors/20 non-progressors. RT-qPCR verification: 73/77.	Incident PTB identified under the Chinese diagnostic standard; participant-level microbiological versus clinical confirmation requires specification. Non-progressors remained disease-free during follow-up.	Prediagnostic serum collected before disease development.	miR-16-5p and miR-451a: lower baseline expression in subsequent progressors. Combined models additionally included BMI and previous TB history.

AFB, acid-fast bacilli; BMI, body mass index; IGRA, interferon-gamma release assay; miRNA, microRNA; PTB, pulmonary tuberculosis; RT-qPCR, reverse-transcription quantitative polymerase chain reaction; TB, tuberculosis; TST, tuberculin skin test.

**Table 11:** Assays, validation, endpoint-specific findings and principal limitations of circulating-miRNA studies of TB progression and treatment-associated changes.

Study	Assay/Normalization	Validation	Endpoint-Specific Findings	Main Limitations
Wagh et al., 2017 [181]	EvaGreen RT-qPCR; pooled healthy-control RNA reference; haemolysed samples excluded.	Single-study diagnostic evaluation; paired treatment analysis in 10 patients. No independent testing described.	Baseline PTB versus healthy controls: miR-16 AUC 1.000; miR-155 AUC 0.967. Treatment association: both moved toward control levels. Resistance association: miR-16 lower in resistant than untreated susceptible PTB.	Small selected cohorts, healthy-control comparison and no independent testing create a risk of inflated performance; AUC 1.000 does not itself demonstrate overfitting. Pooled-reference normalization and different treatment histories further limit interpretation.
Wang et al., 2018 [182]	Small-RNA sequencing followed by SYBR Green RT-qPCR; normalization to starting RNA amount.	Pooled sequencing discovery followed by RT-qPCR evaluation; no independent external validation established.	Untreated versus cured-group classification: AUC 0.894, sensitivity 83.3%, specificity 84.6%, accuracy 84.0%. Untreated PTB versus healthy controls: AUC 0.823, sensitivity 64.8%, specificity 75.5%, accuracy 70.1%. These are group-classification estimates, not validated within-patient monitoring accuracy.	Not entirely paired treatment groups; heterogeneous diagnostic/cure criteria; pooled discovery. Treatment-associated expression differences do not establish microbiological cure or prediction of treatment success.
Duffy et al., 2018 [183]	miRCURY LNA RT-qPCR; elastic-net model with mean normalization across 47 miRNAs.	Model development and blinded validation sets. Participant separation and handling of repeated samples should be documented when assessing validation independence.	Prediagnostic validation: AUC 0.74 within six months before progression; 0.66 for samples up to 24 months before diagnosis. Reduced three-miRNA model: AUC 0.67. Adding miRNAs to a cellular RNA correlate: AUC 0.77 to 0.78.	Limited progressors; repeated observations; time-window-dependent performance; complex normalization and retrospectively reduced signature. Predictive discrimination does not establish a mechanism or general long-term susceptibility prediction.
Barry et al., 2018 [184]	Plasma miRNA profiling followed by RT-qPCR; miR-93 reference.	Exploratory profiling followed by a larger confirmation cohort and serial sampling; independent external classifier validation was not established.	Baseline PTB versus healthy controls: five-miRNA panel AUC 0.976, sensitivity 94%, specificity 88%. Treatment-associated analysis: changes within one month; only miR-29a and miR-99b were reported to return to control levels by completion. These shortened source labels require strand verification and are not a list of all diagnostic-panel components.	Healthy controls without LTBI testing; mixed microbiological/clinical confirmation. Only 11 failures limited the precision of failure assessment; failure-specific accuracy was not supplied. Returning to control levels does not establish cure.
Xin et al., 2022 [185]	Microarray discovery followed by TaqMan RT-qPCR; cel-miR-39 spike-in reference.	Discovery and RT-qPCR verification within the same parent cohort; no external population validation.	MiRNA-only progression discrimination: AUC 0.66 for miR-16-5p and 0.68 for miR-451a; miR-451a sensitivity 38.4%, specificity 90.9%. MiR-451a + BMI + previous TB history: sensitivity 80.8%, specificity 79.2%. Reported adjusted AUC 0.78 requires explicit identification of the model and covariates before attribution.	Clinical-factor imbalance; discovery associations did not survive false-discovery adjustment; haemolysis-sensitive markers; no external validation. Combined-model performance cannot be attributed solely to miRNAs, and incremental value beyond clinical factors was not established by the supplied estimates.

AUC, area under the receiver operating characteristic curve; BMI, body mass index; LNA, locked nucleic acid; LTBI, latent tuberculosis infection; miRNA, microRNA; PTB, pulmonary tuberculosis; RNA, ribonucleic acid; RT-qPCR, reverse-transcription quantitative polymerase chain reaction; TB, tuberculosis.

**Table 12:** Study populations, reference standards and miRNA expression profiles in leukocyte and plasma studies of TB.

Study	Design and Population/Comparator	TB Reference Standard	Matrix	miRNA/Model and Expression
Zhou et al., 2016 [186]	Paediatric case–control study: 28 TB/24 healthy controls. Microarray discovery: 3 culture-confirmed cases/3 controls; RT-qPCR phase: 25 cases/21 controls.	14 culture-positive and 14 culture-negative cases. Culture-negative TB used clinical, exposure, immunological, PCR and radiographic findings with exclusion of other infections.	Peripheral blood leukocytes after erythrocyte lysis; intracellular, not cell-free plasma miRNAs.	Eight-marker panel: miR-1, miR-155, miR-31, miR-146a, miR-10a, miR-125b and miR-150 decreased; the marker labelled miR-29/miR-29b increased. Its exact annotation requires source verification.
Xin et al., 2016 [187]	Exploratory adult case–control study: 10 smear-positive PTB, 13 LTBI and 11 uninfected controls.	PTB: positive sputum smear. LTBI: positive IGRA and TST ≥15 mm, normal radiography. Uninfected controls: negative IGRA/TST, normal radiography.	Plasma	let-7b-5p and miR-30b-5p: higher in PTB versus both comparator groups; no significant LTBI–uninfected difference.
Kathirvel et al., 2020 [188]	Paediatric case–control study: 30 active TB/30 healthy controls. TB included 25 pulmonary and 5 extrapulmonary cases.	At least one positive AFB smear, *M. tuberculosis* complex culture or Xpert MTB/RIF; pretreatment sampling.	Plasma	miR-21, miR-29a, miR-31, miR-146a and miR-155. miR-21, miR-31 and miR-155 increased; miR-146a decreased. miR-29a was not significant in detailed results (*p* = 0.16). Findings concern the mixed active-TB group.
Ulger et al., 2022 [189]	Cross-sectional expression/cytokine study: 44 PTB/44 healthy blood-donor controls.	All PTB cases culture-confirmed.	Plasma	18 of 84 candidate miRNAs differed: 15 increased and 3 decreased. This was an expression profile, not a validated diagnostic panel.

AFB, acid-fast bacilli; IGRA, interferon-gamma release assay; LTBI, latent tuberculosis infection; miRNA, microRNA; PCR, polymerase chain reaction; PTB, pulmonary tuberculosis; RT-qPCR, reverse-transcription quantitative polymerase chain reaction; TB, tuberculosis; TST, tuberculin skin test.

**Table 13:** Assays, validation, reported findings and principal limitations of leukocyte and plasma miRNA studies in TB.

Study	Assay/Normalization	Validation	Comparator-Specific Findings	Main Limitations
Zhou et al., 2016 [186]	Microarray followed by SYBR Green RT-qPCR; U6 normalization; 2^−ΔΔCq^ calculation.	Discovery and separate RT-qPCR phase; independent external classifier validation was not established.	Childhood TB versus healthy controls, leukocyte panel: AUC 0.996, sensitivity 95.8%, specificity 100%. These estimates concern intracellular leukocyte measurements, not plasma concentrations.	Very small discovery set; healthy controls only; model selection/evaluation within the study creates overfitting risk. Mixed culture-positive/clinically diagnosed cases and unresolved miR-29 annotation limit interpretation; culture negativity alone does not demonstrate misdiagnosis.
Xin et al., 2016 [187]	Microarray followed by TaqMan RT-qPCR; miR-1228 reference.	Candidate confirmation within the discovery population; no independent validation.	PTB versus LTBI and uninfected controls: let-7b-5p and miR-30b-5p differed (*p* < 0.01). No AUC, sensitivity, specificity, cutoff or validated classifier reported.	Very small, smear-positive-only cohort; no non-TB pulmonary-disease comparators. Expression associations do not establish diagnostic accuracy.
Kathirvel et al., 2020 [188]	SYBR Green miScript RT-qPCR; SNORD61 reference; comparative-Ct quantification.	Single-cohort candidate evaluation; no independent validation.	Mixed childhood TB versus healthy controls: miR-31 AUC 0.978, sensitivity 93%, specificity 97%; miR-155 AUC 0.953, sensitivity/specificity 90%/90%; miR-146a AUC 0.903, sensitivity 83.3%, specificity 86.7%. miR-29a expression difference was nonsignificant in detailed results (*p* = 0.16).	Small single-centre sample; pulmonary and extrapulmonary cases combined; controls not assessed by TST/IGRA. Reported abstract–results inconsistency for miR-29a requires source verification. Performance is not independently validated or exclusively PTB-specific.
Ulger et al., 2022 [189]	BioMark RT-qPCR after preamplification; global-mean normalization with RNU48 control. Cytokines measured by ELISA.	Cross-sectional association analysis; no independent diagnostic validation.	PTB versus healthy donors: 18/84 miRNAs differed; 11 had reported associations with inflammatory cytokines. No ROC-based diagnostic estimates, cutoff or combined miRNA–cytokine classifier reported.	Healthy-donor comparison only; no LTBI or respiratory-disease controls; multiple-testing correction insufficiently described. Cytokine associations do not establish causal regulation or TB specificity.

AUC, area under the receiver operating characteristic curve; ELISA, enzyme-linked immunosorbent assay; IGRA, interferon-gamma release assay; LTBI, latent tuberculosis infection; miRNA, microRNA; PTB, pulmonary tuberculosis; ROC, receiver operating characteristic; RT-qPCR, reverse-transcription quantitative polymerase chain reaction; SNORD61, small nucleolar RNA, C/D box 61; TB, tuberculosis; TST, tuberculin skin test.

**Table 14:** Direction-Specific Circulating miRNA Profiles in Serum and Plasma across Lung Cancer Histological Categories.

Lung Cancer Type	Matrix	Upregulated miRNAs, *n*	Upregulated miRNAs	Downregulated miRNAs, *n*	Downregulated miRNAs
LUAD	Serum	14	miR-1290 [82]; miR-133a-3p [94]; miR-146b-3p [65]; miR-21 [107]; miR-210 [107]; miR-23a [61]; miR-25-3p [94]; miR-30c-1* [65]; miR-532-5p [94]; miR-550 [65]; miR-566 [65]; miR-616* [65]; miR-939 [65]; miR-942 [107]	2	miR-339-5p [65]; miR-656 [65]
LUAD	Plasma	8	miR-1260a [144]; miR-222-3p [139]; miR-342-5p [139]; miR-425-3p [120]; miR-518f-3p [144]; miR-574-5p [139]; miR-597-5p [144]; miR-628-3p [120]	2	let-7f-5p [144]; miR-532 [120]
SCC	Plasma	5	miR-19a [111]; miR-19b [111]; miR-205 [111]; miR-20a [111]; miR-30b [111]	0	Not reported
SCLC	Serum	2	miR-20a [81]; miR-210 [81]	0	Not reported
SCLC	Plasma	4	miR-320b [153]; miR-375 [129]; miR-375-3p [153]; miR-92a-2-5p [123]	0	Not reported
NSCLC, mixed histology	Serum	28	miR-125b [69]; miR-145 [64]; miR-146b [78]; miR-152 [64]; miR-193a-3p [67]; miR-197 [93]; miR-199a-5p [64]; miR-200b [69]; miR-203 [69]; miR-205 [69,78]; miR-20a [64]; miR-21 [88,93]; miR-21-3p [105]; miR-214 [67]; miR-221 [64]; miR-222 [64]; miR-223 [64,79]; miR-24 [64]; miR-25 [64,67]; miR-29c [78]; miR-30b [78]; miR-320 [64]; miR-34b [69]; miR-429 [69]; miR-483-5p [67]; miR-652 [68]; miR-660 [68]; miR-7 [67]	19	let-7d-5p [84]; let-7e-5p [84]; let-7i [92]; let-7i-3p [66]; miR-10b-5p [84]; miR-1254 [70]; miR-145 [93]; miR-147 [71]; miR-148a-3p [84]; miR-154-5p [66]; miR-15a-5p [84]; miR-192-5p [84]; miR-23a [92]; miR-25-3p [84]; miR-320a [84]; miR-375 [84]; miR-485-5p [70]; miR-574-5p [70]; miR-92a-3p [84]
NSCLC, mixed histology	Plasma	37	miR-1246 [154]; miR-126-3p [125]; miR-1290 [145]; miR-141 [126]; miR-145 [127]; miR-146a [127,138]; miR-150 [145]; miR-16-5p [142]; miR-17 [127]; miR-181c-5p [162]; miR-182 [127]; miR-199b-5p [162]; miR-19b [122]; miR-200b [127,138]; miR-203b [163]; miR-205 [127]; miR-205-5p [125,128,154]; miR-21 [110,122,126,127,130]; miR-21-5p [145]; miR-210 [125,127,145]; miR-210-3p [128,162]; miR-221 [127,140]; miR-23a [135]; miR-23b-3p [162]; miR-30a [117,140]; miR-320a-3p [162]; miR-3662 [118]; miR-4478 [118]; miR-448 [118]; miR-450b [163]; miR-451a [142]; miR-487b-3p [162]; miR-642a [163]; miR-7 [127,138]; miR-877-5p [162]; miR-92a-3p [142,162]; miR-944 [118]	13	miR-1280 [162]; miR-1299 [154]; miR-148a [119]; miR-148b [119]; miR-152 [119]; miR-16-5p [162]; miR-183 [122]; miR-205-5p [162]; miR-215-5p [154]; miR-25 [122]; miR-342-3p [162]; miR-451 [135]; miR-590-5p [141]
NSCLC, histology NR	Serum	12	miR-1-3p [87]; miR-1246 (total) [99]; miR-1246 5′-isomiR (−2 nt) [99]; miR-125b [73]; miR-1291 [87]; miR-182 [80]; miR-205 [80]; miR-21 [72,77]; miR-214-3p [87]; miR-22 [73]; miR-601 [89]; miR-942 [89]	12	let-7a [77]; let-7a-5p [87]; miR-107 [108]; miR-1246 [99]; miR-15b [73]; miR-199-3p [108]; miR-200b [80]; miR-20b-5p [85]; miR-3187-5p [85]; miR-375 [87]; miR-4429 [96]; miR-485-5p [108]
NSCLC, histology NR	Plasma	5	let-7b-5p [150]; miR-17 [137]; miR-21-5p [143]; miR-210-3p [148]; miR-222 [137]	4	miR-126-3p [143]; miR-150-5p [148]; miR-184 [150]; miR-22-3p [150]
NSCLC and SCLC	Serum	6	miR-146a-5p [109]; miR-148b [74]; miR-221 [74]; miR-221-3p [109]; miR-23b [74]; miR-423-3p [74]	1	miR-206 [109]
NSCLC and SCLC	Plasma	23	let-7 [136]; miR-1-3p [159]; miR-126 [133]; miR-130b-3p [159]; miR-145 [133]; miR-15b-5p [159]; miR-18b-5p [159]; miR-197 [133]; miR-200a-3p [159]; miR-20a [133]; miR-21 [133,136]; miR-210 [133]; miR-223 [133]; miR-25 [133]; miR-30a [133]; miR-30d [133]; miR-31 [136]; miR-3662 [115]; miR-423-5p [159]; miR-4478 [116]; miR-448 [116]; miR-544a [131]; miR-944 [115]	6	miR-17 [129]; miR-190b [129]; miR-19a [129]; miR-19b [129]; miR-26b [129]; miR-506 [116]
Lung cancer, histology NR	Serum	0	Not reported	1	miR-155-5p [104]
Lung cancer, histology NR	Plasma	1	miR-31 [114]	0	Not reported

* indicates the passenger strand of a miRNA duplex according to the historical miRNA nomenclature. LUAD, lung adenocarcinoma; NR, not reported; NSCLC, non-small-cell lung cancer; SCC, squamous cell carcinoma; SCLC, small-cell lung cancer. Only miRNAs with a clearly reported upregulated or downregulated direction were included. Reference numbers are shown in square brackets. “Not reported” indicates that no miRNA with the corresponding expression direction was identified within that cancer-type and sample-matrix category.

**Table 15:** Shared Circulating miRNAs Reported in LC and PTB.

Exact	Lung Cancer Evidence	PTB Evidence
let-7b-5p	Upregulated in plasma from NSCLC with histology NR [150]	Upregulated in plasma from active PTB compared with LTBI and uninfected controls [187]
miR-10b-5p	Downregulated in serum from mixed-histology NSCLC [84]	Upregulated in plasma from culture-confirmed PTB [189]
miR-15b-5p	Upregulated in plasma from studies including NSCLC and SCLC [159]	Downregulated in plasma from culture-confirmed PTB [189]
miR-16-5p	Upregulated in one mixed-NSCLC plasma study [142] but downregulated in another [162]	Downregulated in future PTB progressors [185] and in active PTB [189]
miR-21	Predominantly upregulated in serum and plasma across LUAD, mixed NSCLC, NSCLC–NR and combined NSCLC/SCLC cohorts [72,77,88,93,107,110,122,126,127,130,133,136]	No significant active-PTB-specific change in serum [176], but upregulated in pediatric PTB plasma [188]
miR-21-5p	Upregulated in plasma from mixed or unclassified NSCLC [143,145]	Upregulated in untreated PTB and reduced after cure [182]; also included in a time-dependent progression signature [183]
miR-22-3p	Downregulated in plasma from NSCLC with histology NR [150]	Downregulated in plasma from active PTB [175]
miR-25-3p	Upregulated in LUAD serum [94] but downregulated in mixed-NSCLC serum [84]	Downregulated in plasma from culture-confirmed PTB [189]
miR-31	Upregulated in plasma from lung cancer and combined NSCLC/SCLC cohorts [114,136]	Downregulated in pediatric leukocytes [186] but upregulated in pediatric PTB plasma [188]
miR-92a-3p	Downregulated in mixed-NSCLC serum [84] but upregulated in mixed-NSCLC plasma [142,162]	Upregulated in untreated PTB serum and reduced after cure [182]
miR-125b	Upregulated in serum from mixed or unclassified NSCLC [69,73]	Downregulated in serum only with miR-93 normalization [171], unchanged in untreated PTB [181], and downregulated in pediatric leukocytes [186]
miR-133a-3p	Upregulated in LUAD serum [94]	Upregulated in plasma from culture-confirmed PTB [189]
miR-146a	Upregulated in plasma from mixed NSCLC [127,138]	Downregulated in serum under miR-93 normalization [171], in pediatric leukocytes [186] and in pediatric PTB plasma [188]
miR-146a-5p	Upregulated in serum from studies including NSCLC and SCLC [109]	Downregulated in baseline plasma from untreated PTB [184]
miR-148a-3p	Downregulated in mixed-NSCLC serum [84]	Upregulated in plasma during discovery, but not reproduced in a separate validation group [180]
miR-150	Upregulated in plasma from mixed NSCLC [145]	Downregulated in peripheral blood leukocytes from childhood TB [186]
miR-206	Downregulated in serum from cohorts including NSCLC and SCLC [109]	Upregulated in active-PTB serum relative to LTBI and healthy controls [173]
miR-205-5p	Upregulated in several mixed-NSCLC plasma studies [125,128,154] but downregulated in another [162]	Upregulated in plasma from culture-confirmed PTB [189]
miR-210-3p	Upregulated in plasma from mixed or unclassified NSCLC [128,148,162]	Upregulated in plasma from culture-confirmed PTB [189]
miR-320a	Downregulated in mixed-NSCLC serum [84]	Downregulated in active-PTB plasma and further reduced in drug-resistant PTB [175]
miR-320b	Upregulated in SCLC plasma [153]	Downregulated in active-PTB serum by RT-qPCR and Simoa [172]
miR-342-3p	Downregulated in plasma from mixed NSCLC [162]	Higher in PTB than lung cancer in a direct plasma comparison [160]
miR-451a	Upregulated in mixed-NSCLC plasma [142]	Downregulated at baseline in serum from individuals who subsequently progressed from LTBI to active PTB [185]

LTBI, latent tuberculosis infection; LUAD, lung adenocarcinoma; NR, not reported; NSCLC, non-small-cell lung cancer; PTB, pulmonary tuberculosis; SCLC, small-cell lung cancer. Only exact miRNA designations reported in both disease literature were included. Family-level names and mature strands were not merged; for example, miR-21 and miR-21-5p were treated separately. “Concordant” and “opposite” refer to reported expression directions and do not establish diagnostic specificity. Except for miR-342-3p [160], comparisons were inferred across separate studies with different populations, matrices, platforms and comparator groups.

## Data Availability

No new experimental data were generated in this study. All information analyzed and summarized in this review was obtained from previously published studies. The supporting literature search results and evidence extraction tables are provided in the Supplementary Materials. Further inquiries can be directed to the corresponding authors.

## Supplementary Materials

The following supporting information can be downloaded at: https://www.mdpi.com/article/10.3390/diagnostics16213619/s1, Table S1: SANRA assessment of the present narrative review; Table S2: Database-specific search strategies used to identify studies; Tables S2.1.1–S2.1.12: Database-specific screening results from PubMed, Scopus, and Web of Science; Table S2.1: Recent publications and their relevance to microRNA-based diagnosis of pulmonary tuberculosis and differentiation from lung cancer (Google Scholar); Table S2.2: Selected publications on microRNAs in pulmonary tuberculosis and lung cancer, including diagnostic relevance, methodological limitations, and mechanistic evidence; Table S3: Terminology and classification of lung cancer and pulmonary tuberculosis presentations relevant to differential diagnosis; Table S4: Detailed evidence on serum-based circulating miRNAs in lung cancer; Table S5: Detailed evidence on plasma-based circulating miRNAs; Supplementary File S1: Completed PRISMA 2020 checklist [235].
