# Supplementary material for: Circulating MicroRNAs in Lung Cancer and Pulmonary Tuberculosis: Diagnostic Potential, Shared Signatures, and Challenges in Differential Diagnosis
Source: Diagnostics (Basel). 2026 Nov 2;16(21):3619. doi: 10.3390/diagnostics16213619 (PMC13762005; doi:10.3390/diagnostics16213619)
Supplement: Supplementary file 1 [file diagnostics-16-03619-s001.zip › PRISMA_2020_checklist_completed.pdf]

PRISMA 2020 Checklist

Section and Topic	Item #	Checklist item	Location where item is reported
**TITLE**			
Title	1	Identify the report as a systematic review.	Not reported as a systematic review; manuscript identifies itself as a narrative review (pp. 1, 4–5).
**ABSTRACT**			
Abstract	2	See the PRISMA 2020 for Abstracts checklist.	p. 1, Abstract (unstructured; requires revision to PRISMA structured format).
**INTRODUCTION**			
Rationale	3	Describe the rationale for the review in the context of existing knowledge.	pp. 2–4, Section 1 (Introduction).
Objectives	4	Provide an explicit statement of the objective(s) or question(s) the review addresses.	p. 4, Section 1 (final paragraph).
**METHODS**			
Eligibility criteria	5	Specify the inclusion and exclusion criteria for the review and how studies were grouped for the syntheses.	pp. 5–6, Section 2 (eligibility and exclusion criteria; evidence categories).
Information sources	6	Specify all databases, registers, websites, organisations, reference lists and other sources searched or consulted to identify studies. Specify the date when each source was last searched or consulted.	p. 5, Section 2 (databases and last search date, 3 October 2026); Supplementary Table S2.
Search strategy	7	Present the full search strategies for all databases, registers and websites, including any filters and limits used.	p. 5, Section 2; Supplementary Table S2 (database-specific search strategies).
Selection process	8	Specify the methods used to decide whether a study met the inclusion criteria of the review, including how many reviewers screened each record and each report retrieved, whether they worked independently, and if applicable, details of automation tools used in the process.	pp. 5–6, Section 2 (collaborative screening, deduplication, full-text eligibility).
Data collection process	9	Specify the methods used to collect data from reports, including how many reviewers collected data from each report, whether they worked independently, any processes for obtaining or confirming data from study investigators, and if applicable, details of automation tools used in the process.	p. 6, Section 2 (data extraction into evidence tables and collaborative checking; reviewer count per report not specified).
Data items	10a	List and define all outcomes for which data were sought. Specify whether all results that were compatible with each outcome domain in each study were sought (e.g. for all measures, time points, analyses), and if not, the methods used to decide which results to collect.	p. 6, Section 2 (diagnostic/prognostic performance and expression data; exhaustive outcome definition not reported).
	10b	List and define all other variables for which data were sought (e.g. participant and intervention characteristics, funding sources). Describe	p. 6, Section 2 (population,

Section and Topic	Item #	Checklist item	Location where item is reported
		any assumptions made about any missing or unclear information.	biological matrix, miRNA panel, assay, comparator, limitations).
Study risk of bias assessment	11	Specify the methods used to assess risk of bias in the included studies, including details of the tool(s) used, how many reviewers assessed each study and whether they worked independently, and if applicable, details of automation tools used in the process.	Not performed/reported for individual primary studies; SANRA evaluates narrative-review reporting, not study risk of bias (p. 5, Section 2).
Effect measures	12	Specify for each outcome the effect measure(s) (e.g. risk ratio, mean difference) used in the synthesis or presentation of results.	pp. 1, 6, 12–52 (AUC, sensitivity, specificity and other study-reported measures; no prespecified effect-measure framework).
Synthesis methods	13a	Describe the processes used to decide which studies were eligible for each synthesis (e.g. tabulating the study intervention characteristics and comparing against the planned groups for each synthesis (item #5)).	pp. 5–6, Section 2 (evidence grouping by disease, comparator and matrix).
	13b	Describe any methods required to prepare the data for presentation or synthesis, such as handling of missing summary statistics, or data conversions.	Not explicitly reported (p. 6, Section 2 describes narrative extraction but not data transformations).
	13c	Describe any methods used to tabulate or visually display results of individual studies and syntheses.	p. 6, Section 2; evidence tables in Sections 4–6 and Supplementary Materials.
	13d	Describe any methods used to synthesize results and provide a rationale for the choice(s). If meta-analysis was performed, describe the model(s), method(s) to identify the presence and extent of statistical heterogeneity, and software package(s) used.	p. 6, Section 2 (narrative synthesis; meta-analysis not conducted owing to heterogeneity).
	13e	Describe any methods used to explore possible causes of heterogeneity among study results (e.g. subgroup analysis, meta-regression).	pp. 6, 43–54, Sections 2, 6–7 (qualitative discussion of heterogeneity; no formal subgroup/meta-regression analysis reported).
	13f	Describe any sensitivity analyses conducted to assess robustness of the synthesized results.	Not performed/reported (no sensitivity analyses described).
Reporting bias	14	Describe any methods used to assess risk of bias due to missing results in a synthesis (arising from reporting biases).	Not performed/reported

PRISMA 2020 Checklist

Section and Topic	Item #	Checklist item	Location where item is reported
assessment			(no formal reporting-bias assessment described).
Certainty assessment	15	Describe any methods used to assess certainty (or confidence) in the body of evidence for an outcome.	Not performed/reported (no formal certainty-of-evidence assessment described).
**RESULTS**			
Study selection	16a	Describe the results of the search and selection process, from the number of records identified in the search to the number of studies included in the review, ideally using a flow diagram.	pp. 5–7, Section 2 and Figure 1 (study identification and selection counts).
	16b	Cite studies that might appear to meet the inclusion criteria, but which were excluded, and explain why they were excluded.	pp. 5–7, Section 2 and Figure 1 (exclusion categories; specific excluded near-eligible studies not individually cited).
Study characteristics	17	Cite each included study and present its characteristics.	Sections 4–6 (pp. 12–52), study descriptions and evidence tables; Supplementary Tables.
Risk of bias in studies	18	Present assessments of risk of bias for each included study.	Not reported; no individual-study risk-of-bias assessment (p. 5, Section 2).
Results of individual studies	19	For all outcomes, present, for each study: (a) summary statistics for each group (where appropriate) and (b) an effect estimate and its precision (e.g. confidence/credible interval), ideally using structured tables or plots.	Sections 4–6 (pp. 12–52), study-level diagnostic results where available; reporting is not uniform for every outcome/study.
Results of syntheses	20a	For each synthesis, briefly summarise the characteristics and risk of bias among contributing studies.	Sections 4–7 (pp. 12–54), narrative synthesis and methodological limitations; no formal study-level risk-of-bias synthesis.
	20b	Present results of all statistical syntheses conducted. If meta-analysis was done, present for each the summary estimate and its precision (e.g. confidence/credible interval) and measures of statistical heterogeneity. If comparing groups, describe the direction of the effect.	Not applicable: no statistical meta-analysis conducted (p. 6, Section 2).
	20c	Present results of all investigations of possible causes of heterogeneity among study results.	Not applicable: no formal statistical heterogeneity investigations reported (p. 6, Section

PRISMA 2020 Checklist

Section and Topic	Item #	Checklist item	Location where item is reported
			2).
	20d	Present results of all sensitivity analyses conducted to assess the robustness of the synthesized results.	Not applicable: no sensitivity analyses reported.
Reporting biases	21	Present assessments of risk of bias due to missing results (arising from reporting biases) for each synthesis assessed.	Not reported (no formal reporting-bias assessment).
Certainty of evidence	22	Present assessments of certainty (or confidence) in the body of evidence for each outcome assessed.	Not reported (no formal certainty-of-evidence assessment).
**DISCUSSION**			
Discussion	23a	Provide a general interpretation of the results in the context of other evidence.	Sections 6–9 (pp. 43–58), interpretation and translational context.
	23b	Discuss any limitations of the evidence included in the review.	Sections 6–9 (pp. 43–58), limitations of evidence, heterogeneity and validation.
	23c	Discuss any limitations of the review processes used.	pp. 5–6, Section 2 (collaborative rather than independent screening; incomplete Google Scholar exclusion logs); limitations of review process not separately synthesized.
	23d	Discuss implications of the results for practice, policy, and future research.	Sections 7–9 (pp. 52–58), implications for clinical implementation and future research.
**OTHER INFORMATION**			
Registration and protocol	24a	Provide registration information for the review, including register name and registration number, or state that the review was not registered.	Not reported; registration status must be confirmed by authors.
	24b	Indicate where the review protocol can be accessed, or state that a protocol was not prepared.	Not reported; protocol availability/status must be confirmed by authors.
	24c	Describe and explain any amendments to information provided at registration or in the protocol.	Not reported; protocol amendments, if any, must be confirmed by authors.
Support	25	Describe sources of financial or non-financial support for the review, and the role of the funders or sponsors in the review.	p. 59, Funding statement (funder role not specified).
Competing interests	26	Declare any competing interests of review authors.	p. 59, Conflicts of Interest statement.

PRISMA 2020 Checklist

Section and Topic	Item #	Checklist item	Location where item is reported
Availability of data, code and other materials	27	Report which of the following are publicly available and where they can be found: template data collection forms; data extracted from included studies; data used for all analyses; analytic code; any other materials used in the review.	p. 59, Data Availability Statement (“Not applicable”); supplementary search/screening tables described on pp. 5–6.

From: Page MJ, McKenzie JE, Bossuyt PM, Boutron I, Hoffmann TC, Mulrow CD, et al. The PRISMA 2020 statement: an updated guideline for reporting systematic reviews. BMJ 2021;372:n71. doi: 10.1136/bmj.n71. This work is licensed under CC BY 4.0. To view a copy of this license, visit https://creativecommons.org/licenses/by/4.0/
